# Mass Spectrometry-Based Untargeted Metabolomics Reveals the Importance of Glycosylated Flavones in Patterned Lentil Seed Coats

**DOI:** 10.1021/acs.jafc.2c07844

**Published:** 2023-02-08

**Authors:** Fatma M. Elessawy, Derek Wright, Albert Vandenberg, Anas El-Aneed, Randy W. Purves

**Affiliations:** † College of Pharmacy and Nutrition, University of Saskatchewan, Saskatoon S7N 5E5, Saskatchewan, Canada; ‡ Department of Plant Sciences, University of Saskatchewan, Saskatoon S7N 5A8, Saskatchewan, Canada; § Centre for Veterinary Drug Residues, Canadian Food Inspection Agency, Saskatoon S7N 2R3, Saskatchewan, Canada

**Keywords:** lentil, polyphenols, flavones, untargeted metabolomics, dotted seed coat, marbled seed coat, green and black seed coats

## Abstract

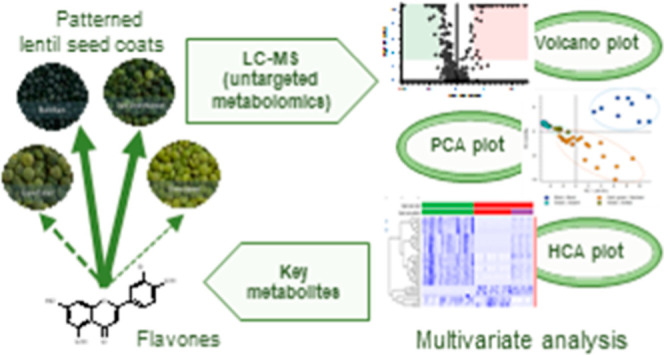

Lentil seed coats are rich in antioxidant polyphenols that are important for plant defense and have potential as valorized byproducts. Although biochemical differences among lentil seed coat colors have been previously studied, differences among seed coat patterns remain largely unexplored. This study used mass spectrometry-based untargeted metabolomics to investigate polyphenol differences among lentil seed coat patterns to search for biochemical pathways potentially responsible for seed coat pattern differences. Comparing patterned with non-patterned green lentil seed coats, 28 significantly upregulated metabolites were found in patterned seed coats; 19 of them were identified as flavones. Flavones were virtually absent in non-patterned seed coats, thereby strongly suggesting a blockage in their flavone biosynthetic pathway. Although the black pattern is not readily discernible on black seed coats, many of the same flavones found in green marbled seed coats were also found in black seed coats, indicating that black seed coats likely have a marbled pattern.

## Introduction

Lentil (*Lens culinaris* Medik.) is a widely grown pulse crop in Canada. It contains many highly nutritive compounds, including proteins, micronutrients, and polyphenols. Being a diverse group of secondary plant metabolites, polyphenols help plants defend themselves against pathogens and environmental stresses.^1,2^ In addition, the antioxidant activity of polyphenols is linked to several health benefits, and they have been incorporated in various medicinal applications.^3−7^ Despite being rich in polyphenols, lentil seed coats have typically been discarded or used as animal feed after the seeds are dehulled. To help better utilize these polyphenol-rich seed coats, greater insights into the relationship between polyphenols and biochemical pathways are needed. For lentils, previous studies have shown that different seed coat colors were primarily correlated to the presence of different amounts of flavan-3-ols and proanthocyanidins.^8,9^ In particular, green seed coats had higher levels of flavan-3-ols and proanthocyanidins compared with brown ones,^10^ whose flavan-3-ol and proanthocyanidin levels were also similar to black seed coats.^8^

Previous studies of seed coat patterns have suggested that polyphenols, mainly flavonoids, are responsible for different seed coat patterns,^11,12^ but changes in the biochemical profiles and pathways remain unclear. Some lentils have no seed coat pattern (absent), whereas others show a dotted or a more complex pattern.^13^ In addition, since the patterns are dark in color, it is difficult to determine whether the black lentil seed coat has a pattern. Vandenberg and Slinkard studied the inheritance of seed coat color and pattern.^13^ The seed coat color is controlled by two genes, each having a dominant and a recessive allele, whereas seed coat pattern is determined by a single gene with five alleles, *Scp*^*m1*^ (marbled-1), *Scp*^*m2*^ (marbled-2), *Scp*^*s*^ (spotted), *Scp*^*d*^ (dotted), and *scp* (absent).^13^ However, correlations between lentil seed coat patterns and their polyphenolic profiles have not been reported.

Determining metabolite profiles from gene expression is critical in understanding plant phenotypes. Untargeted metabolomics using liquid chromatography–high-resolution mass spectrometry (LC-HRMS) has developed into a powerful technique that is used to detect and identify important metabolites and their roles in biochemical pathways.^14^ LC-HRMS untargeted metabolomics has been used to explore differentially expressed metabolites in studies of different colors of wheat seed coats^15^ and *Brassica juncea* seed coats.^16^ In this study, LC-HRMS untargeted metabolomics was integrated with multivariate statistical analysis to investigate differences in polyphenol profiles among different lentil seed coat patterns. Critical polyphenol differences in patterned lentil seed coats will be useful to provide insight into the biochemical pathways responsible for these patterns and also used to determine whether the black lentil seed coat has a pattern. Thus, the aim of this study was to investigate differences in polyphenol profiles among patterned seed coats of *L. culinaris* using LC-HRMS metabolomics to provide insight into the relationship between seed coat patterns and biochemical pathways.

## Materials and Methods

### Chemicals and Reagents

Acetone, optima LC–MS-grade acetonitrile and methanol, and Acros organics formic acid were obtained from Fisher Scientific (Nepean, ON, Canada). Names and supplier information for the chemical standards used in creating an in-house mzVault library for compound identification are given in Table S1. All standards and solvents were stored according to the recommendations of the suppliers.

### Plant Material

Lentil seeds from 18 genotypes having four different colors/patterns (green/absent, green/dotted, dark green/marbled, and black/black as shown in Table 1) were grown in Saskatchewan, Canada, and obtained from the Crop Development Centre at the University of Saskatchewan (Saskatoon, Canada). Each genotype had 3 biological replicates, with each replicate being collected in the same year from different fields (Pullman/2017, Rosthern/2017, and Sutherland/2017) to give a total number of 54 samples. The seed coats were obtained by dehulling the seeds using an abrasive grain testing mill (model TM05, Satake Engineering Co., Hiroshima, Japan) and the seed coats were separated from the dehulled product stream using a column blower (Seedburo Equipment Co., Des Plaines, IL, USA). The seed coats were stored at −80 °C until use.^8^

**Table 1:**
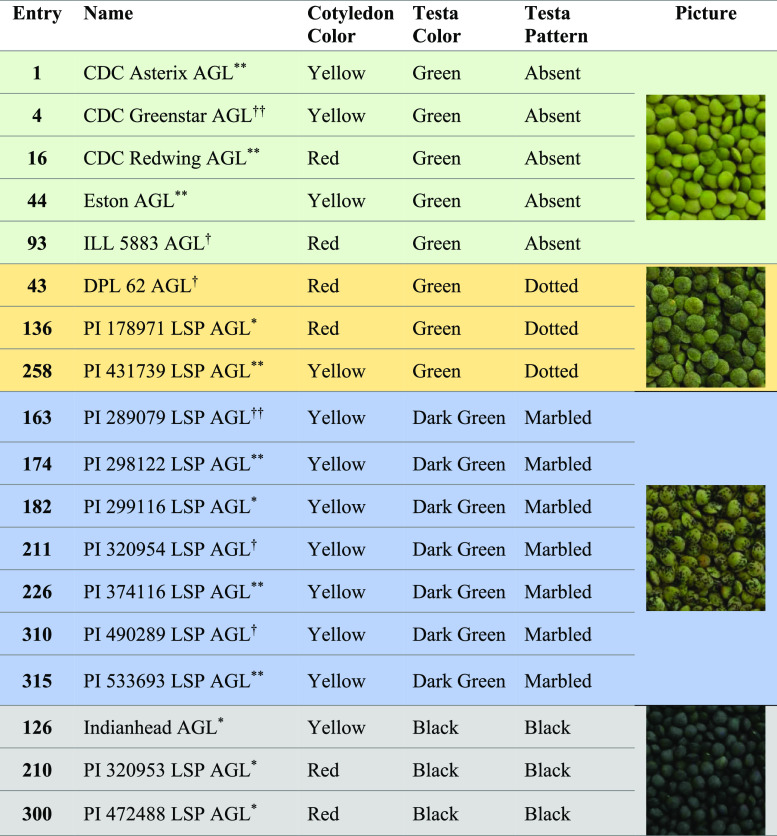
Detailed Description of Colored/Patterned Lentil Seed Coat Genotypes Used in This Study^a^

a Seed size: *small, **medium, ^†^large, ^††^extra large.

### Preparation of Seed Coat Extracts

Seed coat extracts were prepared using a procedure similar to that of Mirali et al.^17^ and modified by Elessawy et al.^8^ An important modification in this study was the use of 50 mg instead of 200 mg of lentil seed coats. This value was changed to reduce the number of adducts, dimers, and multimers formed in the mass spectra while still maintaining sufficient signal intensity for analysis. In brief, ∼50 mg of each sample was placed into microcentrifuge tubes and freeze-dried overnight at −80 °C. A volume of 1 mL of the extraction solvent [acetone/water (70:30 v/v)] was added to the pulverized seed coats, and the samples were mixed and shaken for 1 h at 23 °C at a speed of 1400 rpm. The samples were centrifuged, and the supernatant was transferred into new-labeled tubes. A 200 μL aliquot of each extract was transferred to a new Eppendorf tube, dried down, and reconstituted in 200 μL of MilliQ-water/methanol (90:10 v/v).

### Untargeted Analysis of the Extracts by LC-HRMS

The LC-HRMS instrumentation consists of a Dionex 3000 LC coupled with a Quadrupole-Orbitrap (Thermo Fisher Q-Exactive) mass spectrometer with a HESI (heated ESI) source. For LC separation, an Agilent poroshell 120 PFP column (2.1 × 100 mm, 2.7 μm) was used at a flow rate of 0.35 mL/min. A 30 min run time was used and the mobile phases were water/formic acid (99.9:0.1, v/v) as solvent A and water/acetonitrile/formic acid (9.9:90:0.1, v/v/v) as solvent B. After a 1 min hold at 1% B, gradient elution was performed according to the following conditions: from 1% B to 41% B in 20 min; 41 to 60% B in 4 min, 60 to 80% B in 0.1 min, hold at 80% B for 1.9 min, 80 to 1% B in 0.1 min, then hold at 1% B for 3.9 min. The quadrupole-Orbitrap (Thermo Fisher Q-Exactive) was used to acquire full scan data for the seed coat samples using a mass resolution (full width at half maximum, FWHM, @*m*/*z* 200) of 140,000 in negative mode with a mass range of 140–1800 *m*/*z*.

A QC (quality control) sample, which contains an equal amount of all 54 seed coat samples (18 seed coat genotypes × 3 biological replicates), was injected every 8–10 runs to account for any change in retention time and/or signal intensity, thereby enabling relative quantification. In addition, four ID (identification) samples, which contain an aliquot from all the samples within a color/pattern group (one ID each for green/absent, green/dotted, dark green/marbled, and black/black seed coats), were prepared for obtaining fragmentation data. The scan function “Full scan/DDMS2” was used to acquire data-dependent fragmentation data (i.e., DDMS2) on the most abundant ions detected in full-scan mode. Mass resolution of the full scan analysis was 70,000 (FWHM @*m*/*z* 200) and MS/MS was carried out on the 7 most abundant peaks at a resolution of 17,500 (FWHM @*m*/*z* 200) using a stepped collision energy fragmentation. ID samples were injected three times using collision energies of 10/20, 30/40, and 50/60 eV. MS/MS acquisitions used an exclusion list (*m*/*z* values) of the most intense ions detected from the blank sample.

### Data Analysis

A customized untargeted workflow was developed by adapting an existing workflow in the Compound Discoverer (CD) 3.2 software (Thermo Fisher) to process LC-HRMS raw data. The workflow is similar to one reported previously with some modifications.^18^

The CD software 3.2 parameters used in generating the analysis are shown by the software in a “Summaries” window with tabs including “Workflow,” “Study,” “Grouping & Ratio,” and “Filters.” These tabs are in text format and the outputs for this study are given in the Supporting Information (Summary S1). The Compound Discoverer workflow uses the full-scan accurate mass data to determine the possible molecular formula for each *m*/*z* value and MS/MS spectra from ID samples to help identify compounds. In addition to using Thermo’s mzCloud library, which contains fragmentation data of over 19,000 compounds analyzed with Thermo Orbitrap instrumentation (www.mzcloud.org), the MS/MS spectra were also compared (using the mzVault node) with those in an in-house library at the Core Mass Spectrometry Facility (University of Saskatchewan, Canada). Fragmentation spectra from several other libraries were also used offline, including libraries available in public databases, such as FoodB (foodB.ca), polyphenol-explorer (phenol-explorer.eu), and the human metabolome database (hmdb.ca). The identification levels follow those reported by Sumner et al.,^19^ which include confirmed (1), putative (2), class only (3) and unidentified (4), with the addition of level (2/3) indicating isomeric glycosylated compounds as was done in our previous work.^18^ To focus on polyphenol detection, the results were filtered using a retention time window between 2 and 20 min.^18^

For the volcano plots (differential analysis) when comparing a metabolite between two groups, for it to be considered significantly different, the relative peak areas (calculated for each replicate within a group and the median value was used) needed to be ≥8.0 times different and the *P*-value < 0.001 (>99.9% confidence). *P*-values per group ratio were calculated by ANOVA and TukeyHSD post hoc tests.

## Results and Discussion

**Figure 1:**
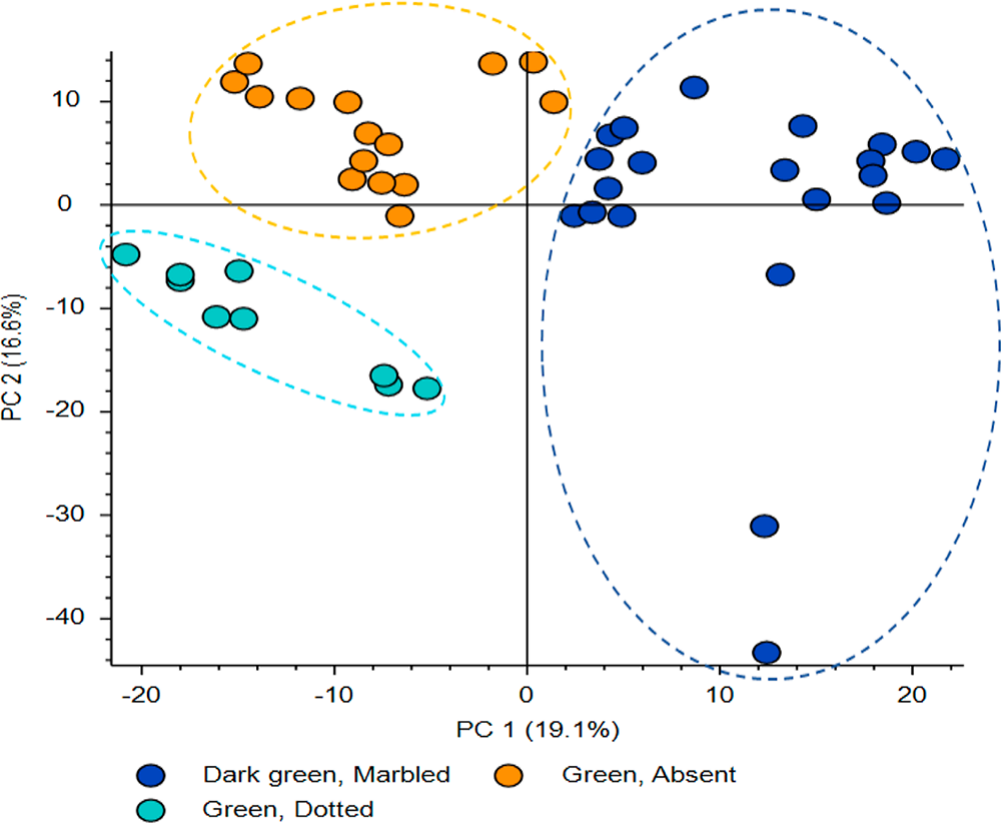
PCA plot (PC1 vs PC2) of green/absent, green/dotted, and dark green/marbled lentil seed coat groups.

LC-HRMS untargeted metabolomics was used to explore the polyphenolic variations among lentil seed coats with different colors/patterns. Initially, green lentil seed coat varieties (no pattern) were compared with two patterned green lentil seed coats (dark green/marbled and green/dotted) to focus on differences due to the seed coat pattern. A Compound Discoverer analysis of the raw data generated a list of metabolites that was used to create a principal component analysis (PCA) plot of the patterned seed coats (Figure 1). The figure shows separate clusters for each green patterned group, which suggests that important biochemical differences exist among these groups. Many abundant metabolites in the green seed coats were proanthocyanidins, which is consistent with our previous study where colored (green, brown, and black) lentil seed coats were found to contain a large number of proanthocyanidins (a mixture of prodelphinidins and procyanidins).^18^

**Figure 2:**
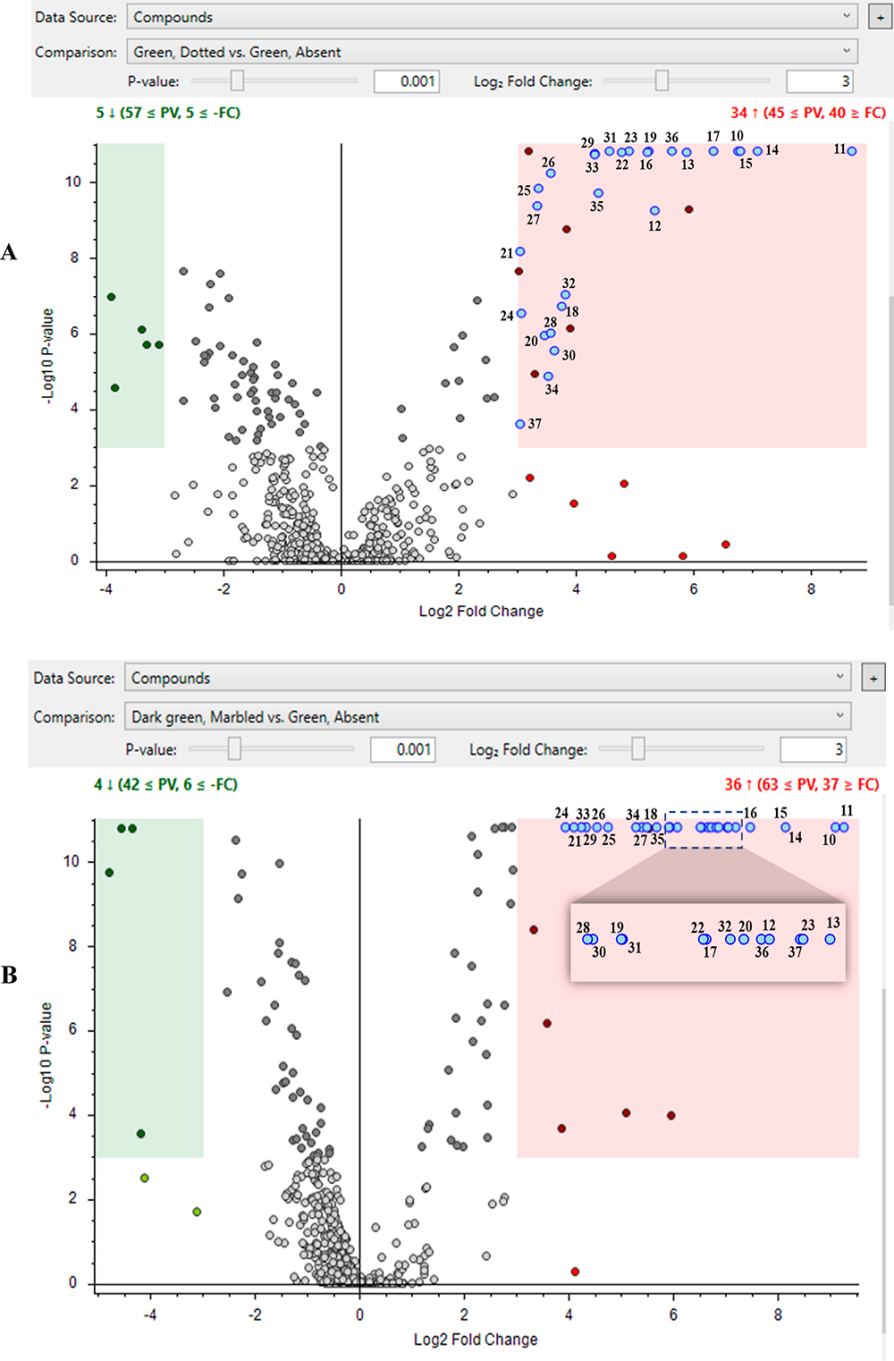
Volcano plots of green/dotted (A) and dark green/marbled (B) vs green absent lentil seed coats. Using a *p*-value 0.001 and a log_2_ fold change of 3 (eightfold change), the upregulated and downregulated metabolites are shown in the red and green highlighted areas, respectively. The blue dots are significantly higher in both dark green/marbled and green/dotted seed coats compared with green/absent seed coats. The metabolite numbers correspond to the same metabolite numbers in Table 2.

Differential analysis using volcano plots (*p*-value 0.001, log_2_ fold change of 3 (eightfold) change) in Figure 2 shows differences in metabolites for green/dotted (A) and dark green/marbled (B) versus the green/absent group. Each dot represents a metabolite detected in the samples; metabolites to the right of zero on the *x*-axis are more abundant in the patterned seed coat, whereas metabolites to the left of zero are more abundant in the absent seed coat. A majority of the metabolites are found in the region near the intersection of the *x-* and *y*-axes where there is no significant difference in abundance; many proanthocyanidins, flavan-3-ols, and flavonols, which are abundant in seed coats, are found in this region. Conversely, the metabolites of most interest are in the green and red highlighted areas, where the differences are considered significant between the two groups (i.e., they meet the criteria of both fold change and *p*-value). Note that dots that were determined to be representing fragment, adduct, or multimer peaks (i.e., tricetin dimer) of another compound already present in the plot were removed (the software attempts to group these types of peaks, but occasionally, some show up separately). Figure 2A,B shows that the number of metabolites that significantly increase in the green/patterned seed coats (red highlighted area) compared with the green/absent seed coats are about 7× more than the number of metabolites that significantly decrease (green-highlighted area). The blue-colored dots in the red highlighted areas in each volcano plot are metabolites that are significantly higher in both dark green/marbled and green/dotted compared with green/absent. Metabolites represented by the blue dots appear to be critical to the existence or absence of a seed coat pattern.

**Figure 3:**
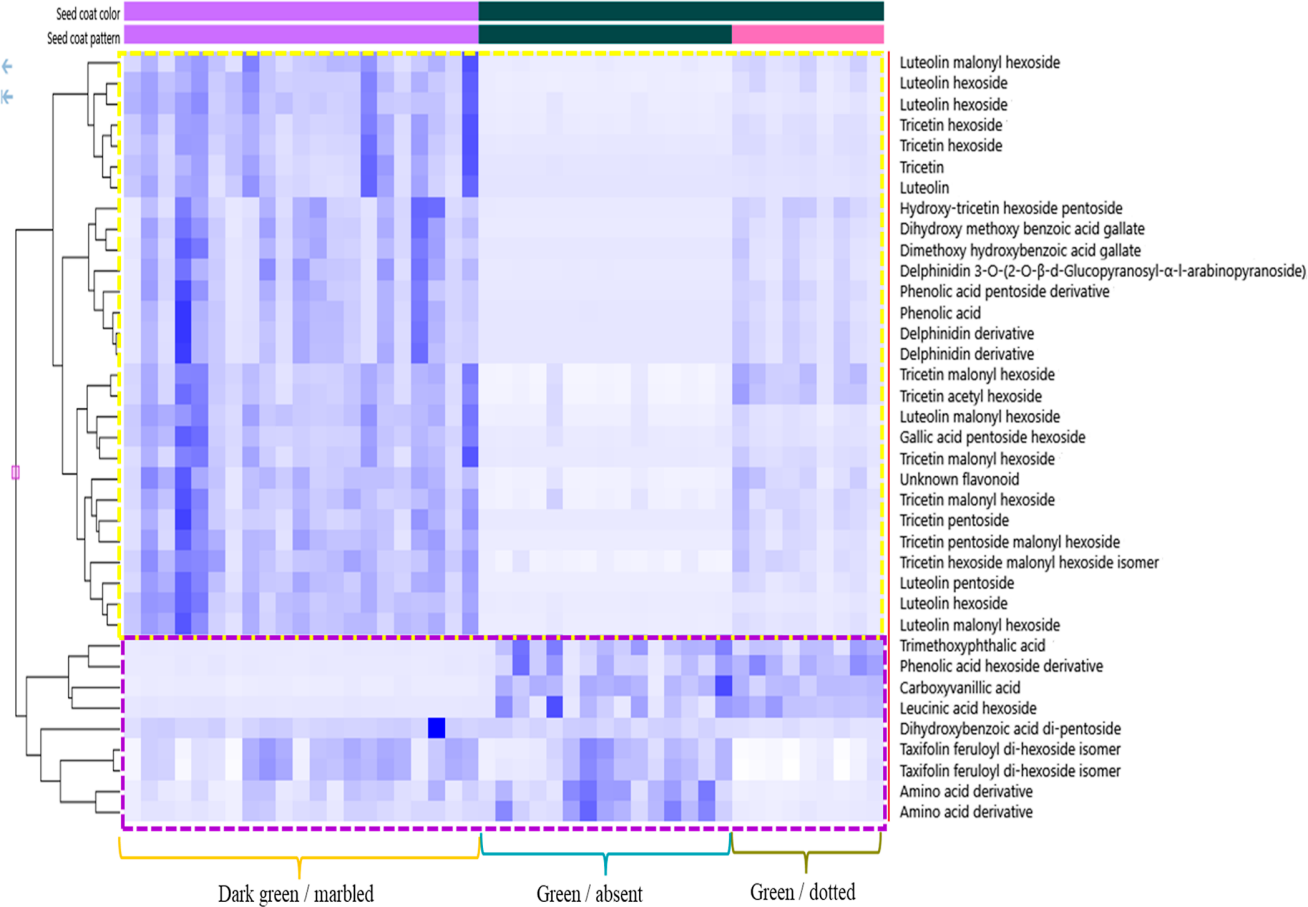
HCA plot of dark green/marbled, green/dotted, and green/absent lentil seed coat using the blue dots (red highlighted areas) and green dots (green highlighted areas) in the volcano plots (Figure 2). Each rectangle represents a metabolite, and its color intensity refers to the relative amount (by area) of that specific metabolite in a specific sample. The plot was divided into two distinct groups of metabolites shown in yellow (upregulated) and purple (downregulated). The ID of these metabolites, including the confidence of the identification, is given in Table 2.

**Figure 4:**
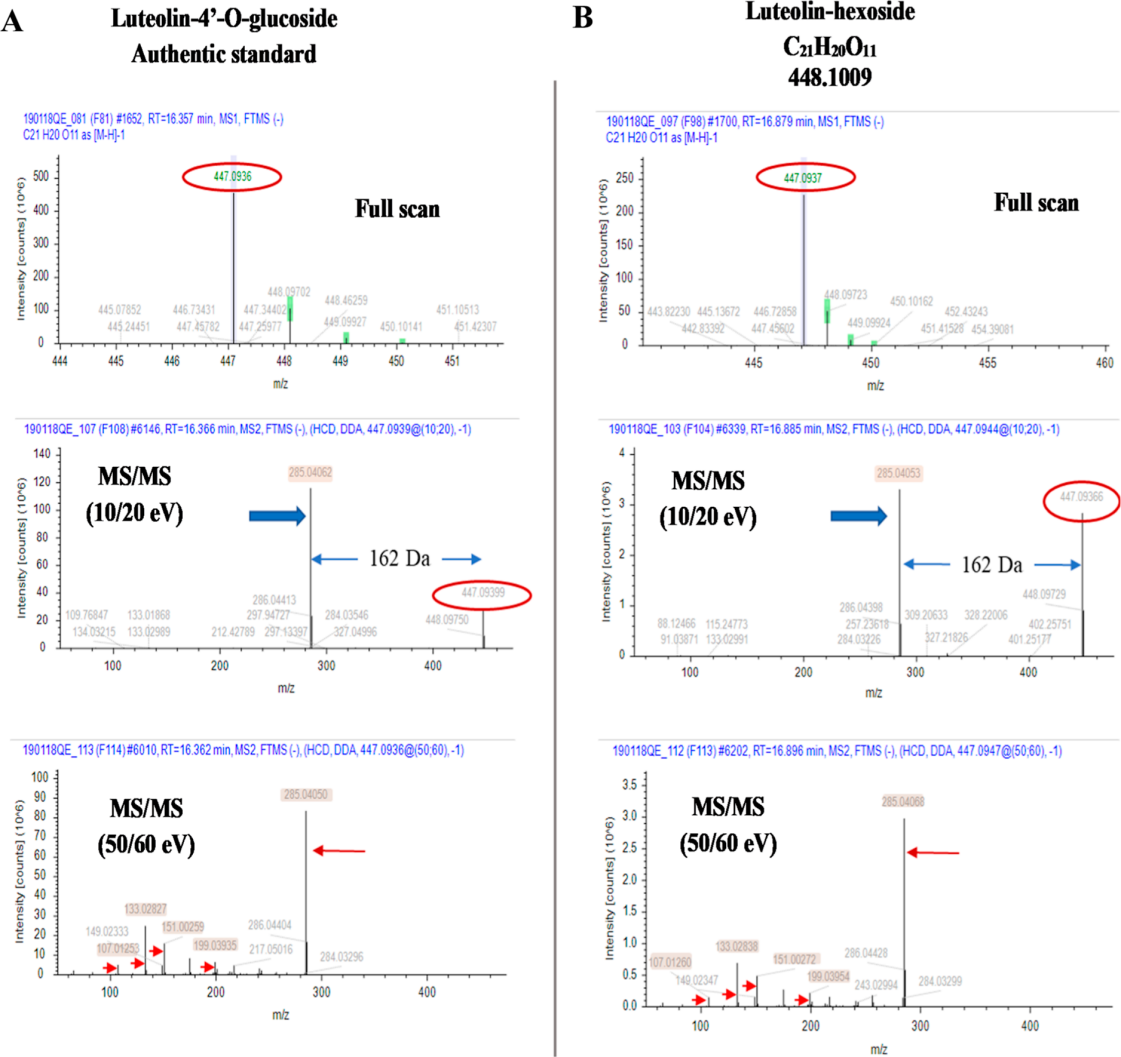
Full-scan and MS/MS spectra of (A) luteolin 4′-O-glucoside authentic standard and (B) glycosylated flavone, identified as luteolin hexoside in Table 2 (compound 30), by comparison to spectra shown in Figure 4A.

The data were also examined using a hierarchal clustering analysis (HCA) plot (as a heat map), which used the metabolites appearing in the regions of significance in both volcano plots in Figure 2; in particular, the blue dots in the red-highlighted area and the green dots in the green-highlighted area. The heat map in Figure 3 shows the distribution of these metabolites among green seed coat groups where the color intensity of each rectangle represents the relative amount (by area) of a specific metabolite in a specific sample. The HCA heat map was grouped into two clusters: upregulated (yellow-outlined cluster) and downregulated (purple-outlined cluster). The metabolites representing each cluster were identified with varying levels of confidence, by comparing their MS/MS spectra, acquired at low (10/20 eV) and high (50/60) collision energies, with online and in-house databases (Table 2). Since our extensive in-house library could only confirm three compounds (level 1), many of the metabolites were putatively identified. As was done previously, the identification levels in the table follow Sumner et al.,^19^ with the addition of level 2/3 indicating a glycosylated metabolite with a putative aglycone identification, but with only a partial identification (e.g., hexose and pentose) of the glycosyl portion.^18^Figure 4 illustrates an example for a level 2/3 identification, since a majority of compounds in the table were identified at this level. Figure 4A shows full-scan and MS/MS spectra for luteolin 4′-O-glucoside from our mzVault library. Compound #27 in Table 2 was confirmed to be luteolin 4′-O-glucoside (level 1 identification). The low collision energy (10/20 eV) spectrum is used to identify the glycosylation (i.e., loss of 162), whereas the higher collision energy (50/60 eV) spectrum provides a fingerprint of the aglycone luteolin with several characteristic ions. The unknown compound (compound # 30 in Table 2) in Figure 4B also shows a loss of 162 (exact mass is 162.0531) at low collision energy and is therefore a hexose, but we cannot tell which type or the location of the attachment. The higher collision energy spectrum has the same fingerprint spectrum as luteolin, thereby enabling the putative identification of luteolin as the aglycone. Compound #30 is therefore listed in Table 2 as luteolin hexoside with a 2/3 identification level. Both the low and high collision energy MS/MS spectra for the 28 up-regulated metabolites in both patterned seed coats are given in Figure S1.

**Table 2:**
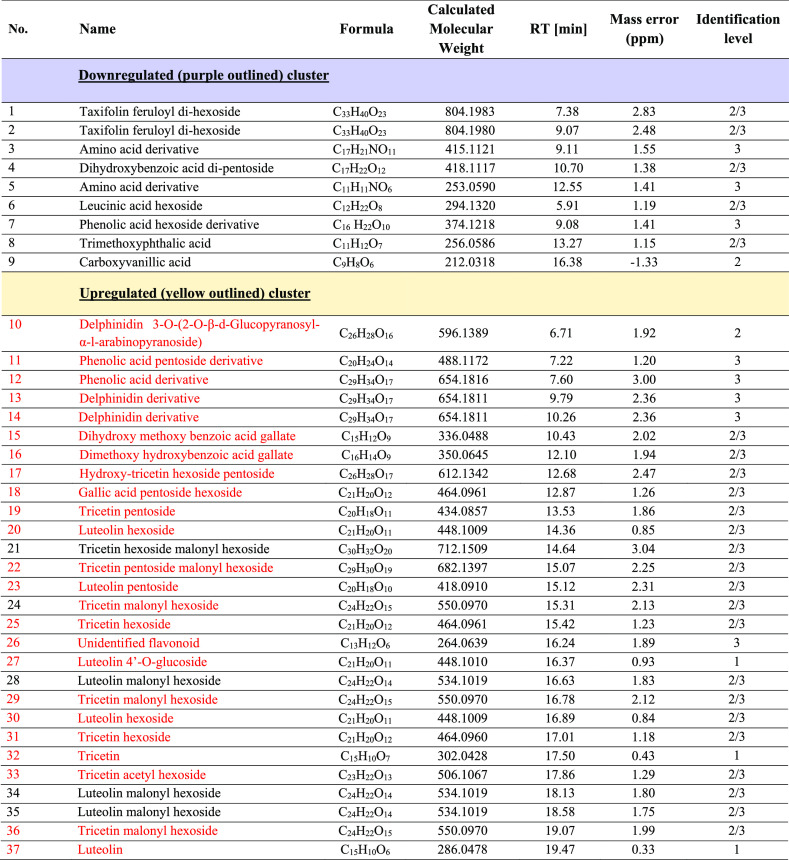
Identification of the Compounds Used in the Hierarchal Clustering Plot (HCA) in Figure 3^a^

a Identification levels are: confirmed (1), putative (2), isomeric (2/3), class only (3), and unidentified (4). Compounds written in red are upregulated in the volcano plots for green/patterned and black/black seed coats versus green/absent seed coats (23 compounds).

Although a large number of proanthocyanidins were present in all samples, they are not observed in the HCA plot, or in Table 2, suggesting that the proanthocyanidins are not responsible for the seed coat pattern differences. The HCA plot contains 37 metabolites, with only 9 being significantly higher in the absent seed coats compared with either patterned seed coat; that is, four are higher versus dark green/marbled and five are higher versus green/dotted (downregulated, purple-outlined cluster). A majority of these nine metabolites are either phenolic or amino acids. Out of the remaining 28 metabolites in the HCA plot, which were significantly upregulated in *both* green patterned seed coats, five are identified as phenolic acids, indicating that there are some differences in the types of phenolic acids produced between absent and patterned seed coats, but that both types of seed coats contain phenolic acids. The two most noticeable differences in the HCA plot are that the upregulated metabolites in the green patterned seed coats contain 3 delphinidin derivatives and 19 flavones (17 glycosylated and 2 aglycones). As these delphinidins and flavones were at a significantly lower concentration in the absent seed coat, and no other flavones or delphinidins specific to the absent seed coats were found, this indicates that the pathways for their synthesis have been largely blocked, which appears to be the likely reason for the absence of the seed coat pattern. The flavones in particular were highly abundant in the patterned seed coats as tricetin, luteolin, tricetin hexoside, and luteolin hexoside were four of the top five metabolites detected by Compound Discoverer when sorted by maximum peak area. In addition, the peak areas of 16 of the 19 flavones were at least 50% higher in dark green/marbled compared with green dotted (two of these were ∼10× higher), whereas the other 3 were at about the same level. This result strongly suggests a dependence of the amount of the pattern on the flavone concentration. The presence of high flavone amounts in the patterned seed coats might indicate a possible evolutionary event due to interaction with pathogens which led to induced biosynthesis of flavones as a defensive mechanism.^2,20^

**Figure 5:**
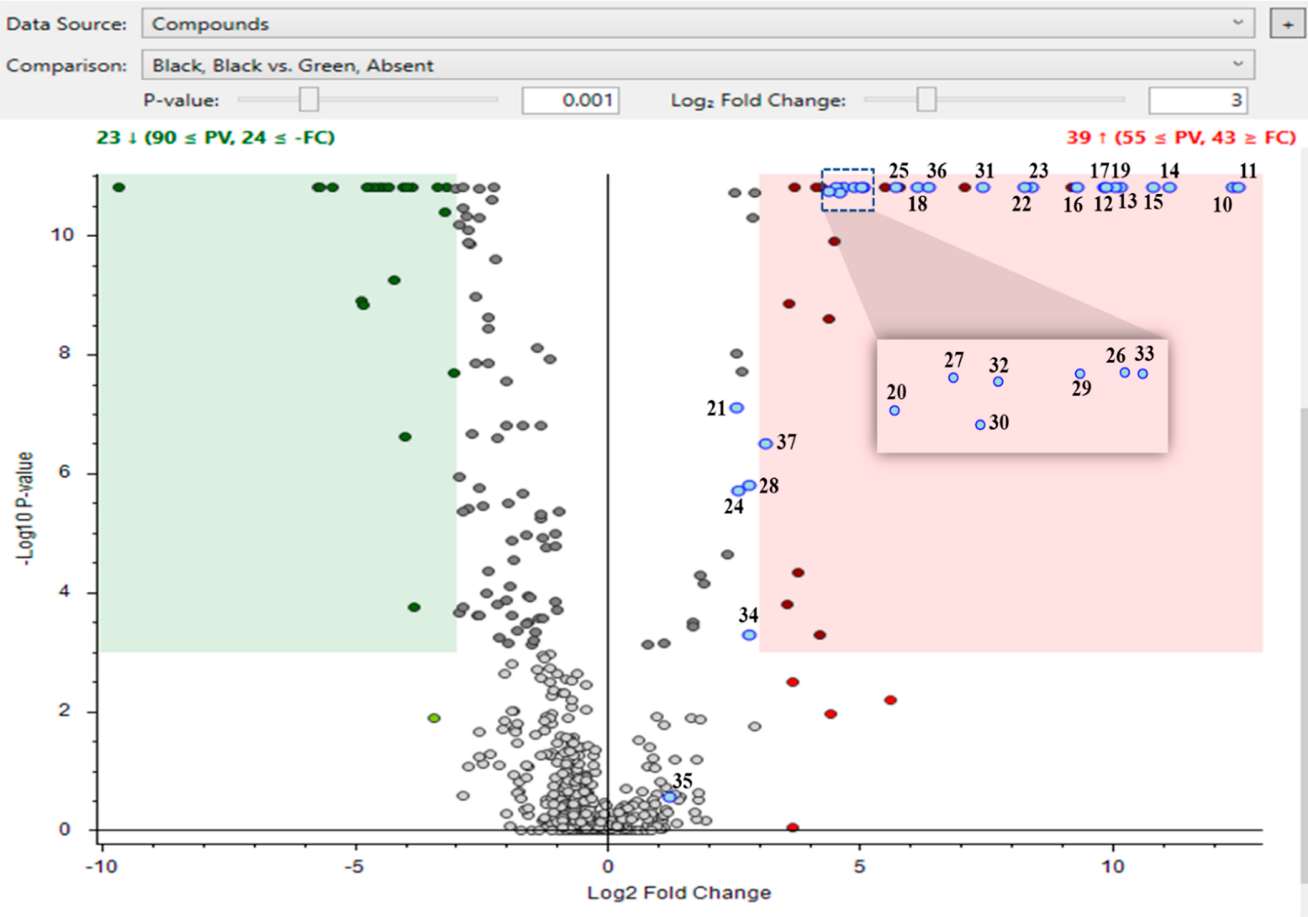
Volcano plot of the black/black vs green/absent seed coats. Using a *p*-value 0.001 and log_2_ fold change of 3 (eightfold change), the upregulated and downregulated metabolites are shown in the red and green highlighted areas, respectively. The blue dots are significantly higher in both dark green/marbled and green/dotted seed coats compared with green/absent seed coats. The metabolite numbers correspond to the same metabolite numbers in Table 2.

Since the seed coat is black in color in black/black seed coats, it is difficult to determine visually whether the dark pattern observed on the green seed coats is also present on the black/black seed coats. If the pattern is present, then many of the same upregulated metabolites should also be observed. Figure 5 shows a volcano plot that was used to compare the black/black and green/absent seed coats. The figure is similar to the plots shown in Figure 2, except that the number of metabolites that significantly change when comparing black/black seed coats to the green/absent is higher than what is observed in Figure 2. It is expected that in addition to the seed coat pattern, there are differences as a result of the seed coat color.^10^ In particular, a much greater number of metabolites have significantly increased in green/absent (in the green highlighted area in Figure 5), presumably due to the differences in seed coat color. Importantly, the metabolites represented by blue dots in Figure 2 (the 28 metabolites upregulated in both green/patterned seed coats identified in the yellow-outlined cluster in Table 2) are also represented by blue dots in Figure 5. Of these 28 metabolites, all are higher in black/black seed coat compared with green/absent, with 23 being significantly higher (another 4 more would also be significant at a log_2_ = 2.5), indicating that black/black seed coats have high levels of virtually all of the significantly upregulated metabolites found in both of the green patterned genotypes. Since the black/black seed coat also contains these metabolites believed to be critical to the presence of a pattern, this strongly suggests that the black/black seed coat also contains a pattern. Out of the 19 flavones in the yellow cluster, 8 are higher in the black/black and 11 are higher in the dark green/marbled, which suggests that these two groups have similar total amounts of flavones and, possibly, similar patterns.

The metabolites written in red in Table 2 are significantly upregulated for *all* three groups (black/black, dark green/marbled, and green/dotted) versus green/absent (23 metabolites). Table S1 also shows all of the significantly upregulated metabolites for *any* of the three groups compared with the green/absent seed coats (from Figures 2 and 5). Although the black/black seed coat shares many of the significantly upregulated compounds present in green/patterned seed coats, there are also some differences among these three groups. Some of the significantly upregulated metabolites in black/black that were not significantly upregulated in the green patterned seed coats include gallic acid, gallocatechin containing metabolites, and flavone glycosides including *C*-glycosylated apigenin (shown in Table S1).

**Figure 6:**
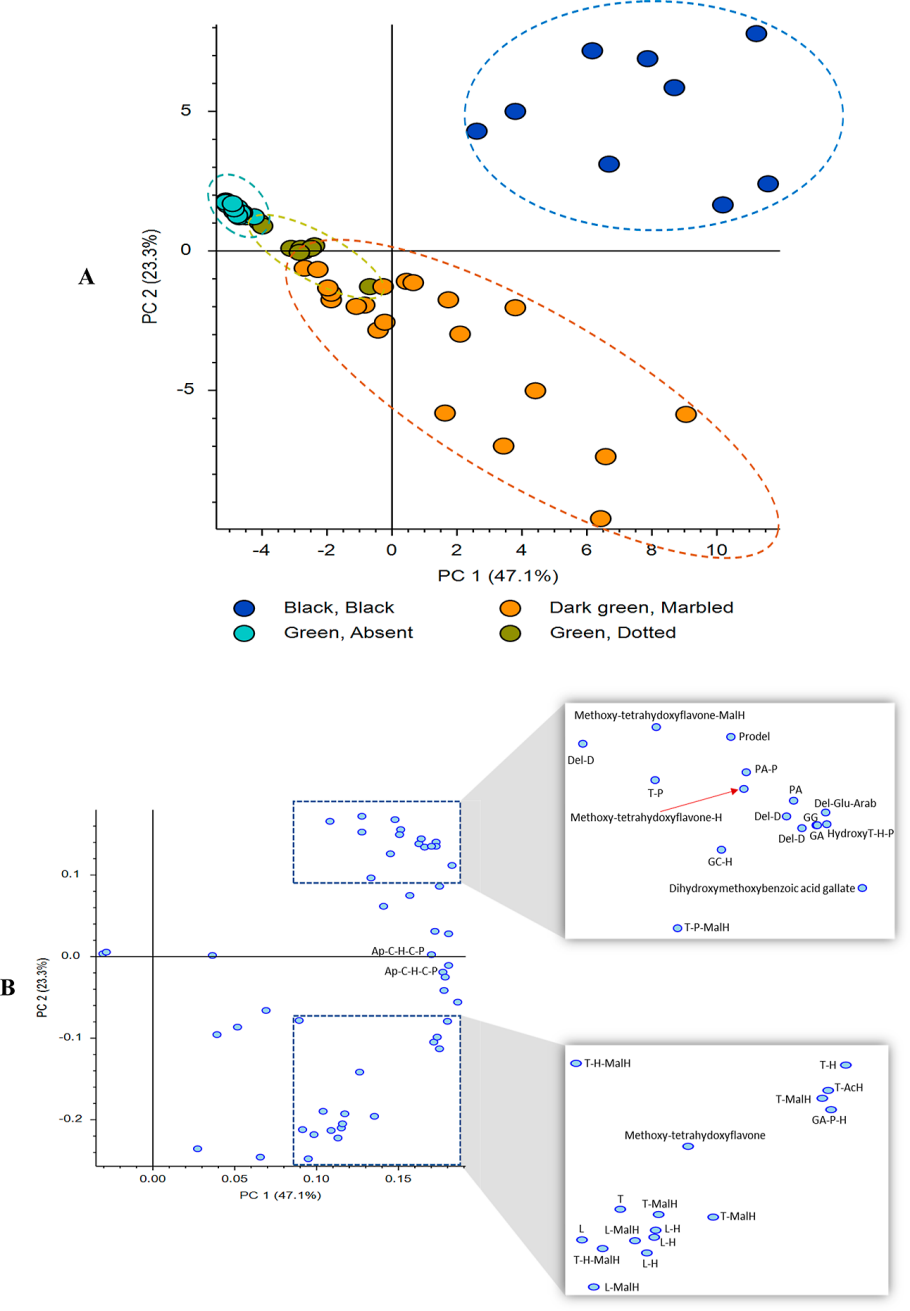
PCA (A) scores plot and (B) loadings plot (PC1 vs PC2) of colored/patterned lentil seed coat groups including the upregulated metabolites in all seed coats. Blue-colored dots in dotted boxes refer to the metabolites that are expected to be contributing the most to the seed coat patterns. These metabolites were putatively identified in Tables 2 and S1. Ap: apigenin, T: tricetin, L: luteolin, GA: gallic acid, Del: delphinidin, D: derivative, Prodel: prodelphinidin, H: hexoside, P: pentoside, AcH: acetyl hexoside, MalH: malonyl hexoside, C–H: C-hexoside, C–P: C-pentoside, Glu: glucoside, Arab: arabinoside, PA: phenolic acid, GC: gallocatechin, GG: prodelphinidin dimer.

Although the delphinidin compounds have much higher peak areas in the black seed coat, the peak areas of the remaining highlighted metabolites are not as predictable, with some being higher in black, and others higher in green/patterned seed coats. A PCA plot using the 53 total upregulated metabolites (in all seed coats) found in green patterned and/or black/black seed coats compared with green/absent is shown in Figure 6. The black seed coats cluster somewhat differently from the green seed coats, and mostly along PC2. The loadings plot (Figure 6B) indicates that delphinidin metabolites, gallic acids, and gallocatechin metabolites are in the upper region of PC2, whereas luteolin hexosides and luteolin malonyl hexosides are in the lower region. Important in this plot is how the green seed coats cluster. The dark green/marbled and green/absent seed coats show separate clusters, whereas the green/dotted cluster intersects with both green/absent and dark green/marbled. Many of the flavones are far right in the loadings plot in PC1. These PCA clusters, along with the relative areas of the peaks, support our hypothesis that the pattern in lentil seed coats is correlated to the concentration of flavones. At the lowest concentration of flavones, the pattern is absent, and then as the flavone concentration increases, the pattern is dotted, and then is marbled at the highest flavone concentrations in lentil seed coats. Although these seed coat patterns are visually separated into groups, there appears to be more of a continuum in the pattern as is suggested by the slight overlap between green/dotted with both green/absent and dark green/marbled seed coats (Figure 6). The results of this study have implications for the enzymes responsible for the biosynthetic pathways in transcriptomic analysis.^21,22^ The black/black and dark green/marbled seed coats have similar amounts of flavones, but the pathway appears partially blocked in the dotted seed coats and almost completely blocked in the non-patterned (i.e., absent) seed coats. Genetic studies are needed to confirm this hypothesis.

In summary, untargeted metabolomics was used to investigate differences in polyphenolic profiles among colored-patterned lentil seed coats. Differential analyses revealed the presence of several significantly upregulated flavones when comparing lentil seed coats with patterns to those with no pattern. Furthermore, the levels of flavones were the highest in the black/black (8 flavones) and the dark green/marbled (11 flavones), whereas green/dotted had lower levels and green/absent had the lowest levels. These findings strongly suggest that the flavone branch in the polyphenol biosynthetic pathway is partially blocked in the dotted seed coats and further blocked in the absent (i.e., no pattern) lentil seed coats. Although these data present strong evidence, to confirm this pathway blockage, additional studies need to be carried out, including the analysis of the critical genes in this pathway. In addition to the analysis of critical genes, future studies will examine the importance of these seed coat flavones in disease resistance and their influence on antioxidant properties.

## Supporting Information Available

List of polyphenols, including subclasses and supplier (and country) for the standards used in creating the mzVault library, identification of the upregulated compounds in each seed coat group and/or shared with other groups (from the volcano plots in Figures 2 and 5), full-scan and MS/MS spectra of the putatively identified metabolites in Table 2, and parameters used in generating the analysis of patterned lentil seed coat samples using Compound Discoverer 3.2 software (PDF)
