# Supplementary material for: Mass Spectrometry-Based Untargeted Metabolomics Reveals the Importance of Glycosylated Flavones in Patterned Lentil Seed Coats
Source: J Agric Food Chem. 2023 Feb 8;71(7):3541–9. doi: 10.1021/acs.jafc.2c07844 (PMC9951240; doi:10.1021/acs.jafc.2c07844)
Supplement: Supplementary file 1 — jf2c07844_si_001.pdf [file jf2c07844_si_001.pdf]

Mass spectrometry-based untargeted metabolomics reveals the importance of glycosylated flavones in lentil seed coat patterns.

Fatma M. Elessawy,[1] Derek Wright,[2] Albert Vandenberg,[2] Anas El-Aneed,[1] and Randy W. Purves [1,2,3,*]

[1]College of Pharmacy and Nutrition, University of Saskatchewan, Saskatoon, SK, Canada

[2]Department of Plant Sciences, University of Saskatchewan, Saskatoon, SK, Canada

[3]Centre for Veterinary Drug Residues, Canadian Food Inspection Agency, Saskatoon, SK, Canada

*Corresponding author

Email address: randy.purves@usask.ca or randy.purves@inspection.gc.ca, Tel: +1 306-385-7843

Table S1. List of polyphenols, including subclasses and supplier (& country) for the standards used in creating the mzVault library. Labelled internal standards (IS) are indicated in bold.

**Compound name**	**Supplier (Country)**
**Anthocyanins**	
Pelargonidin 3-*O*-glucoside	Extrasynthese, France
Malvidin 3-*O*-glucoside (oenin)	Extrasynthese, France
Pelargonidin 3,5-di-*O*-glucoside	Extrasynthese, France
Peonidin 3,5-di-*O*-glucoside	Extrasynthese, France
Peonidin 3-*O*-glucoside	Extrasynthese, France
Cyanidin 3,5-di-*O*-glucoside	Extrasynthese, France
Cyanidin 3-*O*-rhamnoside	Extrasynthese, France
Delphinidin 3,5-di-*O*-glucoside	Extrasynthese, France
Delphinidin 3-*O*-rhamnoside	Extrasynthese, France
Delphinidin 3-β-D-Glucoside	Santa Cruz Biotech, USA
Cyanidin 3-*O*-glucoside	Extrasynthese, France
Malvidin 3,5-di-*O*-glucoside	Extrasynthese, France
Malvidin 3-*O*-galactoside	Extrasynthese, France
Cyanidin 3-*O*-rutinoside	Extrasynthese, France
Peonidin 3-*O*-rutinoside	Extrasynthese, France
Delphinidin 3-*O*-rutinoside	Extrasynthese, France
Pelargonidin 3-*O*-rutinoside	Extrasynthese, France
**Chalcones**	
Phloretin	Sigma Aldrich, USA
Xanthohumol	Extrasynthese, France
**Hydroxycoumarins**	
4-Hydroxy-6-methylcoumarin	Sigma Aldrich, USA
**Flavonols**	
Kaempferol-3-*O*-rhamnoside	Sigma Aldrich, USA
Fisetin	Extrasynthese, France
Isorhamnetin	Sigma Aldrich, USA
Kaempferol	Sigma Aldrich, USA
Kaempferol 3-*O*-robinoside-7-*O*-rhamnoside (robinin)	Sigma Aldrich, USA
Kaempferol 3-*O*-rutinoside-4'-glucoside	Sigma Aldrich, USA
Kaempferol-3-*O*-D-galactoside	Sigma Aldrich, USA
Kaempferol-3-*O*-glucoside	Extrasynthese, France
Kaempferol-3-*O*-rutinoside	Extrasynthese, France

Kaempferol-7-*O*-glucoside	Extrasynthese, France
Kaempferol-7-*O*-neohesperidoside	Extrasynthese, France
Myricetin	Sigma Aldrich, USA
Myricetin-3-*O*-rhamnoside	Extrasynthese, France
Quercetin	Extrasynthese, France
Quercetin-3,4'-di-*O*-glucoside	Extrasynthese, France
Quercetin-3-*O*-galactoside	Extrasynthese, France
Quercetin-3-*O*-glucoside (Isoquercetrin)	Extrasynthese, France
Quercetin-3-*O*-rhamnoside (Quercitrin)	Extrasynthese, France
Quercetin-3-*O*-rutinoside (Rutin)	Extrasynthese, France
Quercetin-4′-*O*-glucoside (Spiraeoside)	Sigma Aldrich, USA
Tiliroside	Extrasynthese, France
**Quercetin-d3**	Toronto Research Chemicals, CAN
**Flavones**	
5,7-Dimethoxyflavone	Sigma Aldrich, USA
Apigenin	Extrasynthese, France
Apigenin-7-*O*-glucoside	Extrasynthese, France
Apigenin-7-*O*-neohesperidoside	Extrasynthese, France
Apigenin-7-*O*-rutinoside	Extrasynthese, France
Apigenin-8-C-glucoside (Vitexin)	Extrasynthese, France
Chrysin	Extrasynthese, France
Diosmetin	Sigma Aldrich, USA
Diosmetin-7-*O*-rutinoside	Sigma Aldrich, USA
Flavone	Sigma Aldrich, USA
Luteolin	Extrasynthese, France
Luteolin-3',7-di-*O*-glucoside	Extrasynthese, France
Luteolin-4'-*O*-glucoside	Extrasynthese, France
Luteolin-7-*O*-glucoside	Extrasynthese, France
Luteolin-7-*O*-rutinoside	Sigma Aldrich, USA
Luteolin-8'-*C*-glucoside	Extrasynthese, France
Tricetin	Extrasynthese, France
Tangeretin	Extrasynthese, France
Vitexin-2'-*O*-rhamnoside	Extrasynthese, France
**Flavan-3-ols**	
(+)-Catechin	Extrasynthese, France
(-)-catechin gallate	Extrasynthese, France

(-)-Epicatechin	Extrasynthese, France
(-)-Epicatechin gallate	Extrasynthese, France
(-)-Epigallocatechin	Extrasynthese, France
(-)-Epigallocatechin gallate	Extrasynthese, France
(-)-Gallocatechin	Sigma Aldrich, USA
**±-Catechin-2,3,4-$^{13}C_3$**	Sigma Aldrich, USA
**Flavanones**	
Eriocitrin	Extrasynthese, France
Eriodictyol	Extrasynthese, France
Flavanone	Sigma Aldrich, USA
Hesperetin	Sigma Aldrich, USA
Hesperetin-7-*O*-rutinoside	Sigma Aldrich, USA
Isosakuranetin	Extrasynthese, France
Narigenin-7-*O*-rutinoside	Sigma Aldrich, USA
Naringenin	Sigma Aldrich, USA
**Dihydroflavonols**	
Dihydromyricetin	Sigma Aldrich, USA
Dihydrokaempferol	Sigma Aldrich, USA
Taxifolin (dihydroquercetin)	Sigma Aldrich, USA
**Stilbenes**	
Resveratrol	Sigma Aldrich, USA
Resveratrol-3-β-mono-D-glucoside (Polydatin)	Santa Cruz Biotech, USA
**Resveratrol-(4-hydroxyphenyl-$^{13}C_6$)**	Sigma Aldrich, USA
**Hydroxybenzoic acids**	
3,4-Dihydroxybenzoic acid	Sigma Aldrich, USA
4-amino salicylic acid	Sigma Aldrich, USA
4-hydroxybenzoic acid	Sigma Aldrich, USA
Gallic acid	Sigma Aldrich, USA
Salicin	Sigma Aldrich, USA
Syringic acid	Extrasynthese, France
Vanillic acid	Sigma Aldrich, USA
Vanillic acid-4-β-D-glucoside	Sigma Aldrich, USA
Vanillin	Sigma Aldrich, USA
**Vanillin-(ring-$^{13}C_6$)**	Sigma Aldrich, USA
**4-hydroxybenzoic acid - $^{13}C_7$**	Sigma Aldrich, USA
**Hydroxycinnamic acids**	
Caffeic acid	Sigma Aldrich, USA

Chlorogenic acid	Sigma Aldrich, USA
Ferulic acid (trans)	Sigma Aldrich, USA
p-Coumaric acid (trans)	Sigma Aldrich, USA
trans-3-hydroxycinnamic acid	Sigma Aldrich, USA
**Ferulic acid-D_3**	Toronto Research Chemicals, CAN
**Isoflavones**	
Genistein	Sigma Aldrich, USA
Prunetin	Sigma Aldrich, USA
**Procyanidins**	
Procyanidin A2	Sigma Aldrich, USA
Procyanidin B1	Sigma Aldrich, USA
Procyanidin B2	Sigma Aldrich, USA
Procyanidin B3	AdooQ, USA
Procyanidin C1	Sigma Aldrich, USA

Table S2. Identification of the upregulated compounds in each seed coat group and/or shared with other groups (from the volcano plots in **Figure 2 & 5**. Identification levels are: confirmed (1), putative (2), isomeric (2/3), class only (3) and unidentified (4).

Name	Formula	Calculated Molecular Weight	RT [min]	Mass error (ppm)	Identification level
**Black/black (not significantly upregulated in dark green/marbled nor green/dotted) seed coats**					
Gallic acid isomer	C7 H6 O5	170.02086	4.22	-3.91	2/3
Gallocatechin hexoside	C21 H24 O12	468.12703	8.07	0.54	2/3
Myricetin hexoside feruloyl hexoside	C33 H38 O24	818.17751	8.23	2.70	2/3
Prodelphinidin dimer (GG)	C30 H26 O14	610.13321	9.10	1.57	2/3
Prodelphinidin derivative/adduct	C41 H40 O25	932.18812	9.99	2.42	3
Apigenin C-hexoside C-pentoside	C26 H28 O14	564.14917	11.26	2.24	2/3
Apigenin C-hexoside C-pentoside	C26 H28 O14	564.14913	11.39	2.18	2/3
Tricetin di-hexoside	C27 H30 O17	626.14967	14.58	2.18	2/3
Methoxy-tetrahydroxy-flavone hexoside	C22 H22 O12	478.11169	16.39	1.18	2/3
**Dark green/marbled (not significantly upregulated in green/dotted nor black/black) seed coats**					
Leucinic acid hexoside	C12 H22 O8	294.13182	4.99	1.19	2/3
Unknown hexoside	C13 H24 O8	308.14773	7.13	1.98	3
Luteolin deoxyhexoside di-pentoside	C30 H32 O19	696.15593	14.06	3.10	2/3

**Green/dotted (not significantly upregulated in dark green/marbled nor black/black) seed coats**					
Dihydroxybenzoic acid hexoside	C13 H16 O9	316.07983	8.78	1.24	2/3
Dihydroxybenzoic acid malonyl hexoside	C16 H18 O12	402.08069	12.69	2.15	2/3
Tricetin hexoside malonyl hexoside	C30 H32 O20	712.14986	16.36	1.63	2/3
Unknown acetyl hexoside	C23 H24 O11	476.1326	19.60	1.54	3
**Black/black and dark green/marbled (not significantly upregulated in green/dotted) seed coats**					
**Unidentified**		**582.15974**	**9.76**		**4**
Jasmonic acid derivative	C20 H34 O11	450.21095	11.05	1.87	3
Heliotropic acid gallate	C15 H10 O8	318.03813	15.67	1.77	2/3
Methoxy-tetrahydroxy-flavone hexoside	C22 H22 O12	478.11174	16.93	1.28	2/3
Methoxy-tetrahydroxy-flavone	C16 H12 O7	316.05892	19.28	1.94	2/3
**Black/black and green/dotted (not significantly upregulated in dark green/marbled) seed coats**					
Delphinidin derivative	C32 H40 O22	776.20224	7.22	1.43	3
Methoxy-tetrahydroxy-flavone malonyl hexoside	C25 H24 O15	564.11265	17.65	2.00	2/3
**Dark green/marbled and green/dotted (not significantly upregulated in black/black) seed coats**					
Tricetin hexoside malonyl hexoside	C30 H32 O20	712.15086	14.64	3.04	2/3
Tricetin malonyl hexoside	C24 H22 O15	550.09704	15.31	2.13	2/3
Luteolin malonyl hexoside	C24 H22 O14	534.10193	16.63	1.83	2/3
Luteolin malonyl hexoside	C24 H22 O14	534.10192	18.13	1.80	2/3
Luteolin malonyl hexoside	C24 H22 O14	534.10189	18.58	1.75	2/3

10. Delphinidin 3-O-(2-O-β-d-Glucopyranosyl-α-l-arabinopyranoside)	$C_{26}H_{28}O_{16}$	596.1389	6.71	1.92	2

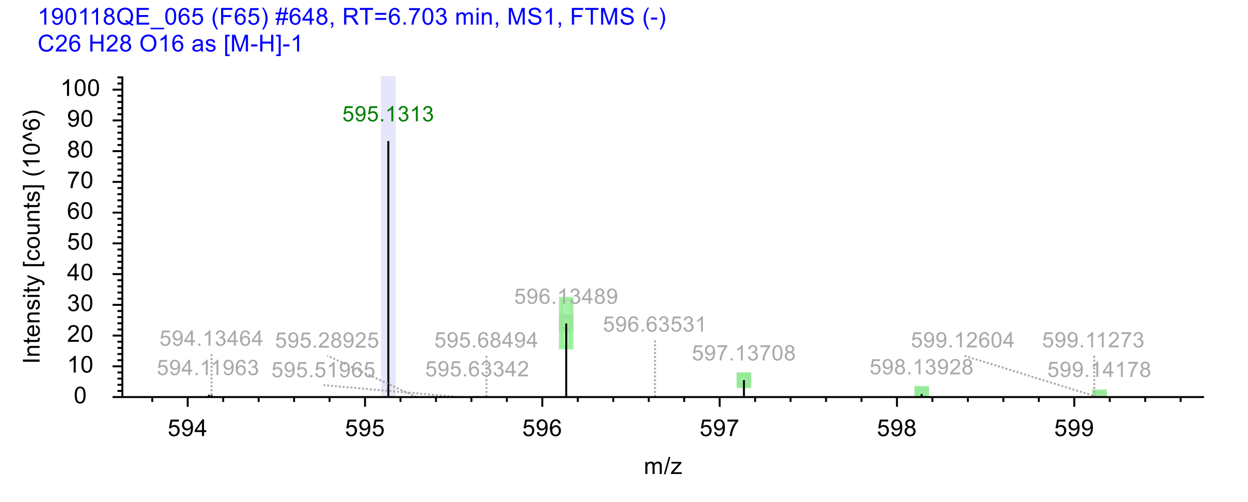

10/20 eV

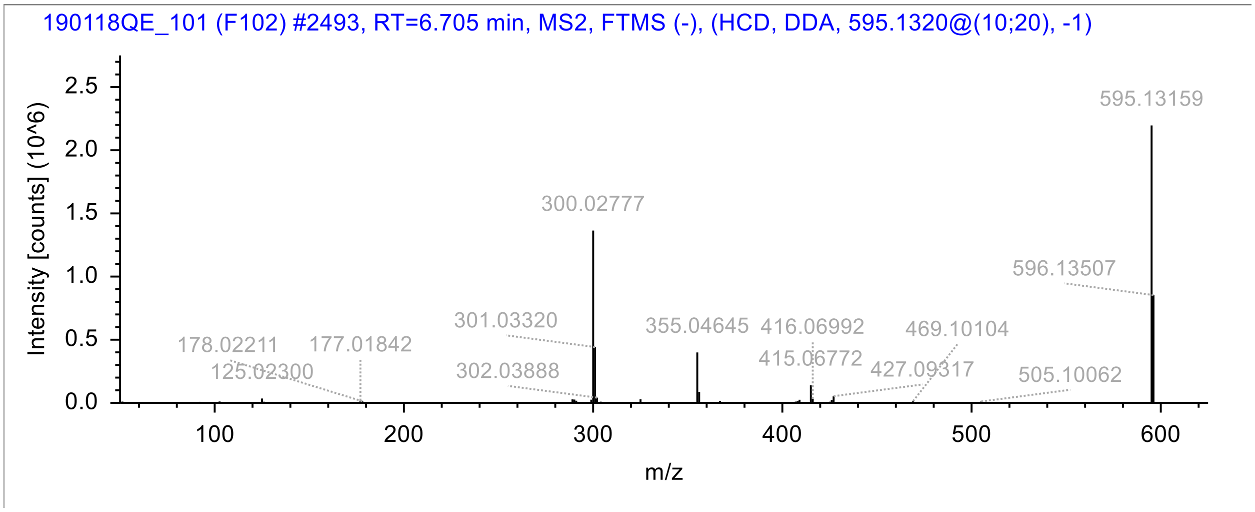

50/60 eV

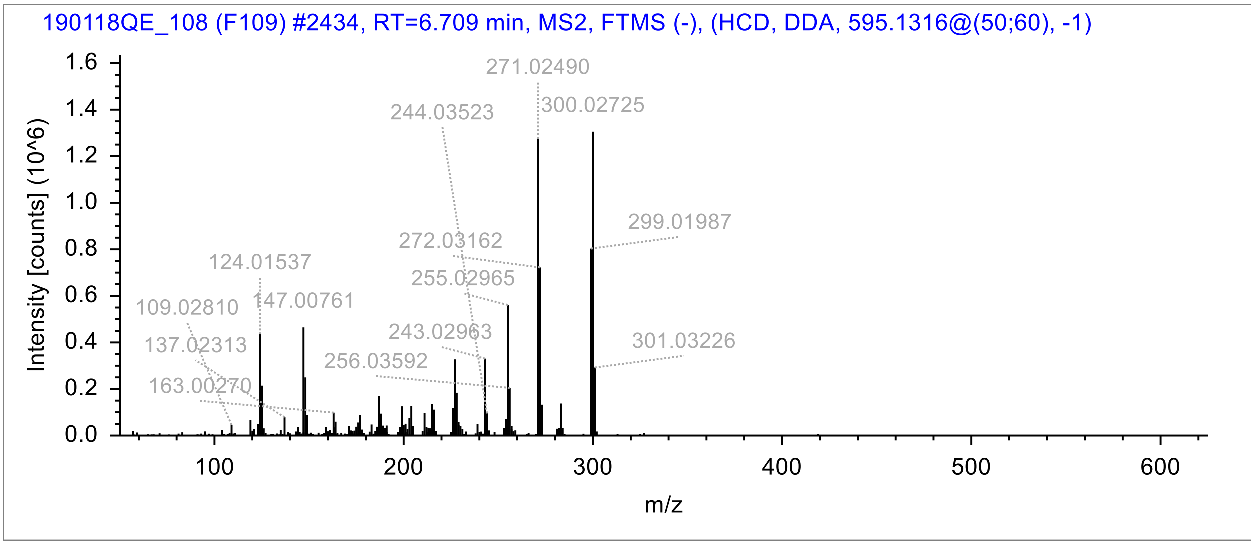

11. Phenolic acid pentoside derivative	$C_{20}H_{24}O_{14}$	488.1172	7.22	1.20	3

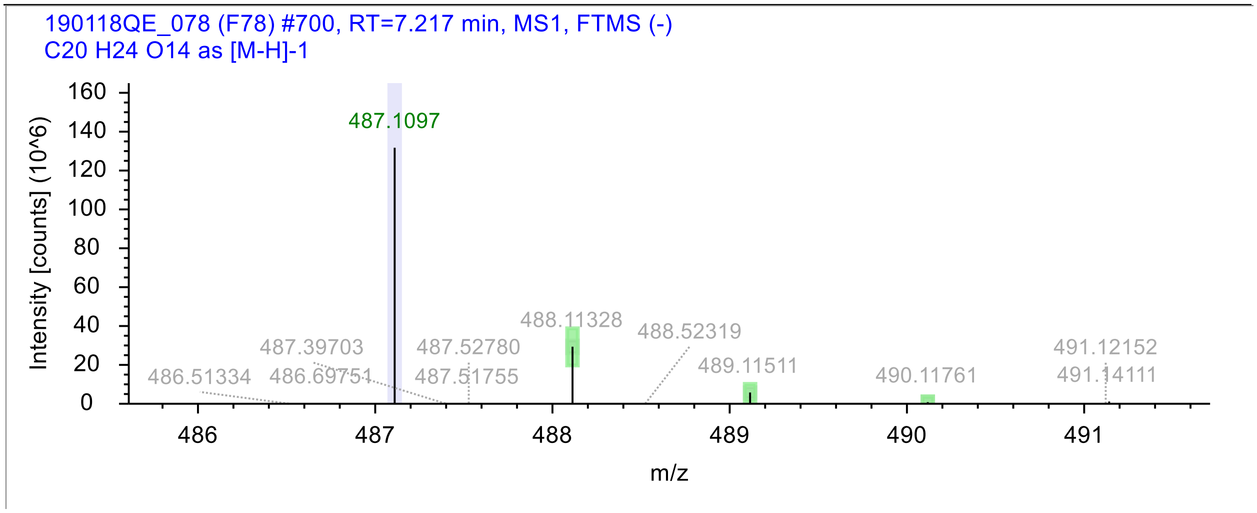

10/20 eV

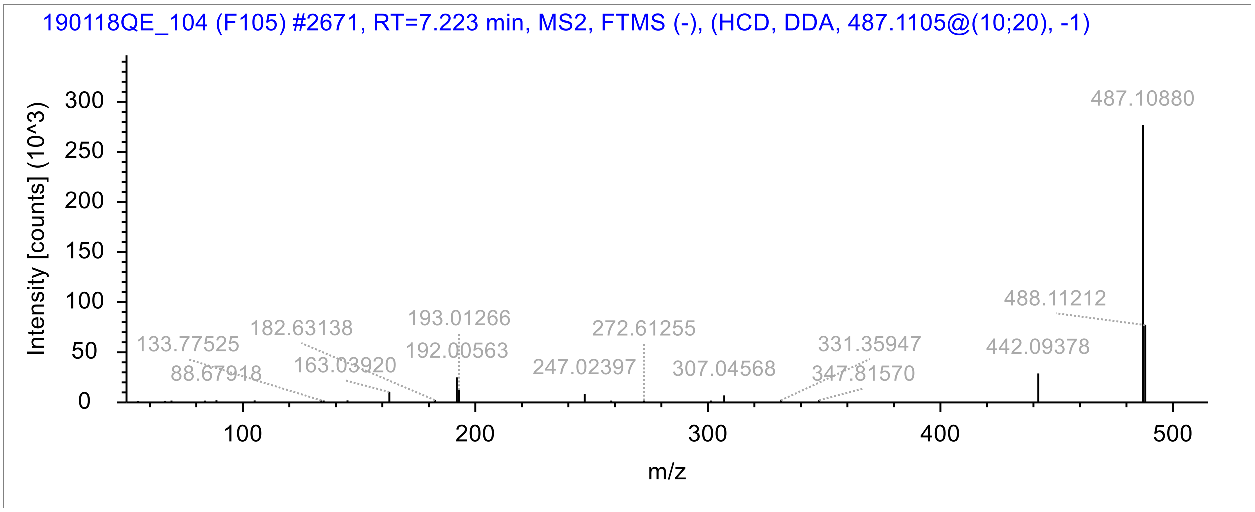

50/60 eV

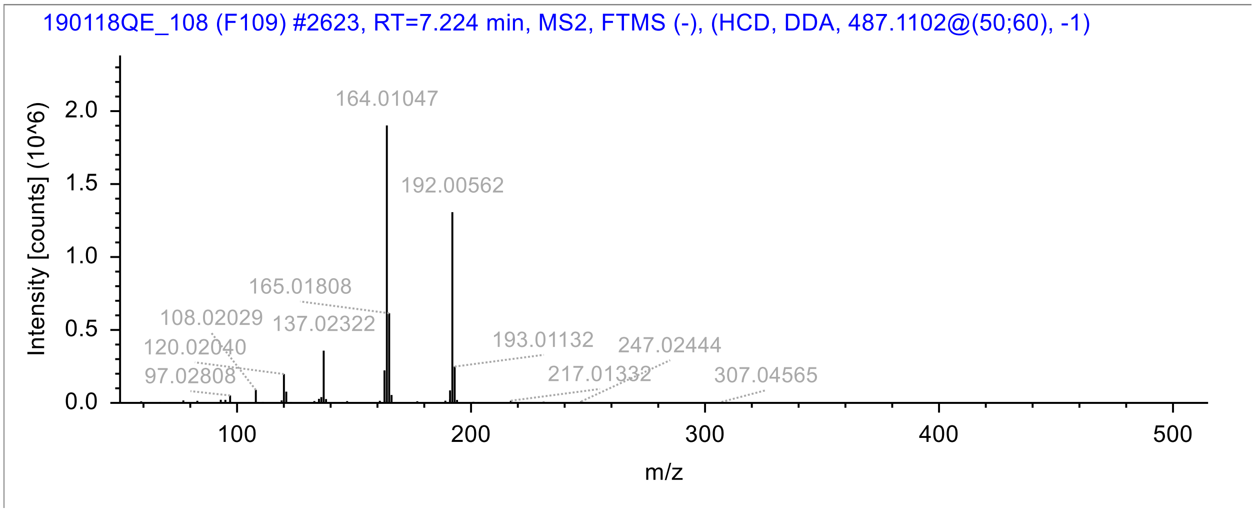

12. Phenolic acid derivative	$C_{29}H_{34}O_{17}$	654.1816	7.60	3.00	3

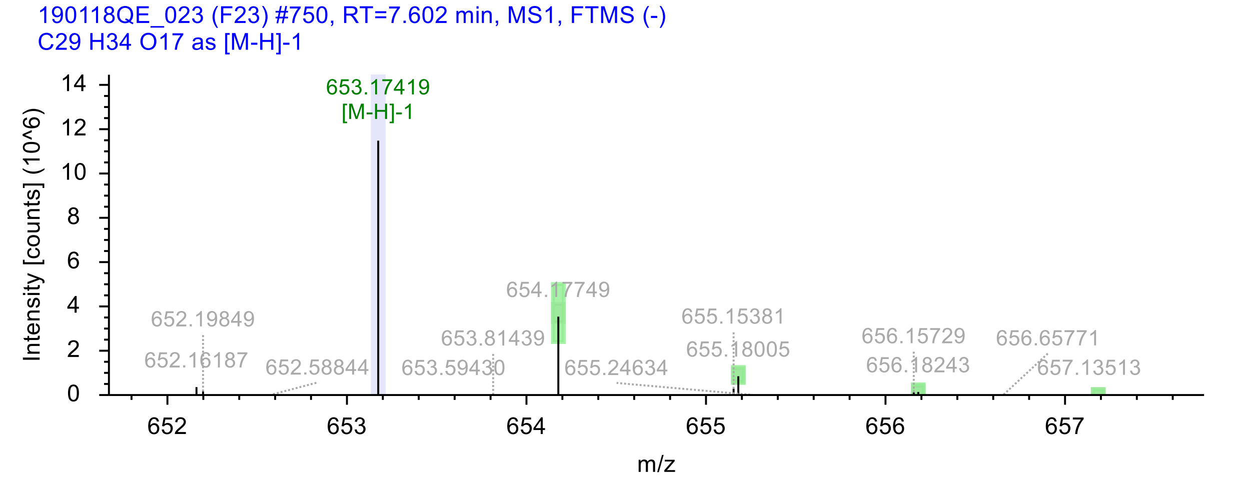

10/20 eV

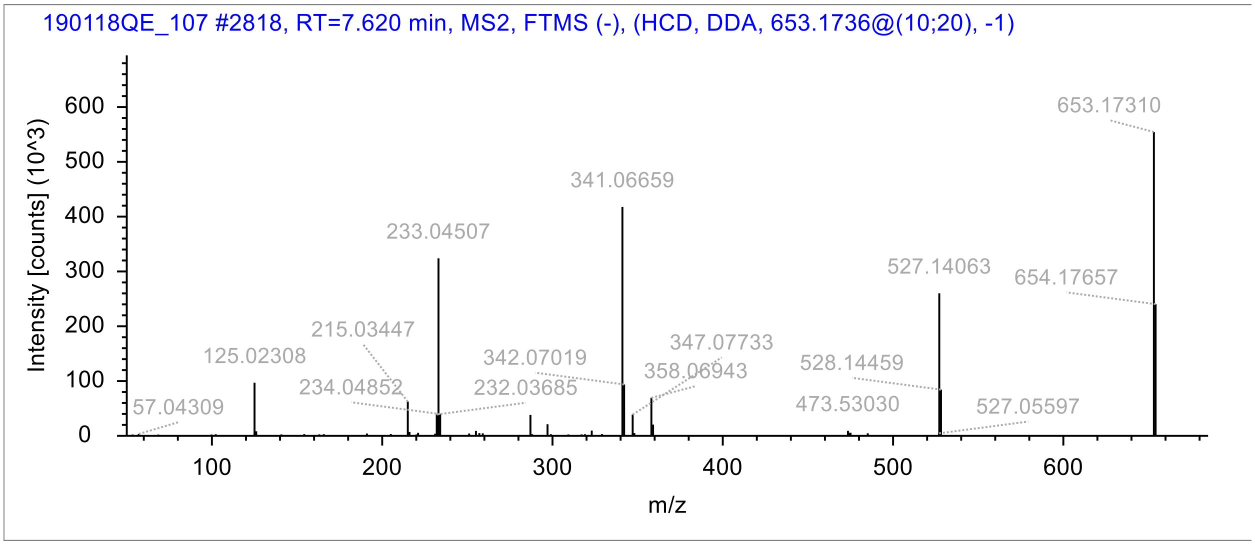

50/60 eV

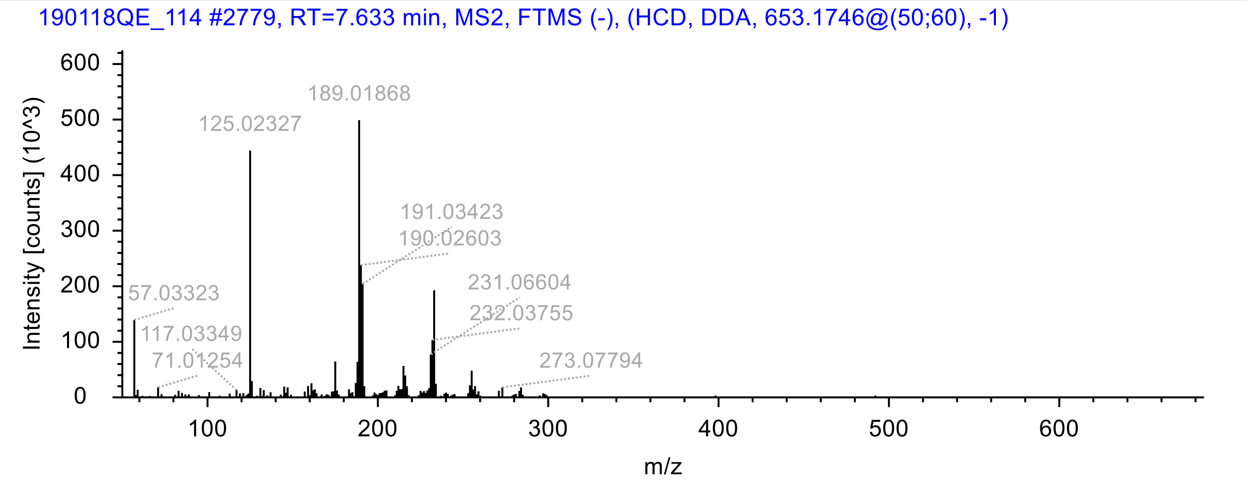

13. Delphinidin derivative	$C_{29}H_{34}O_{17}$	654.1811	9.79	2.36	3

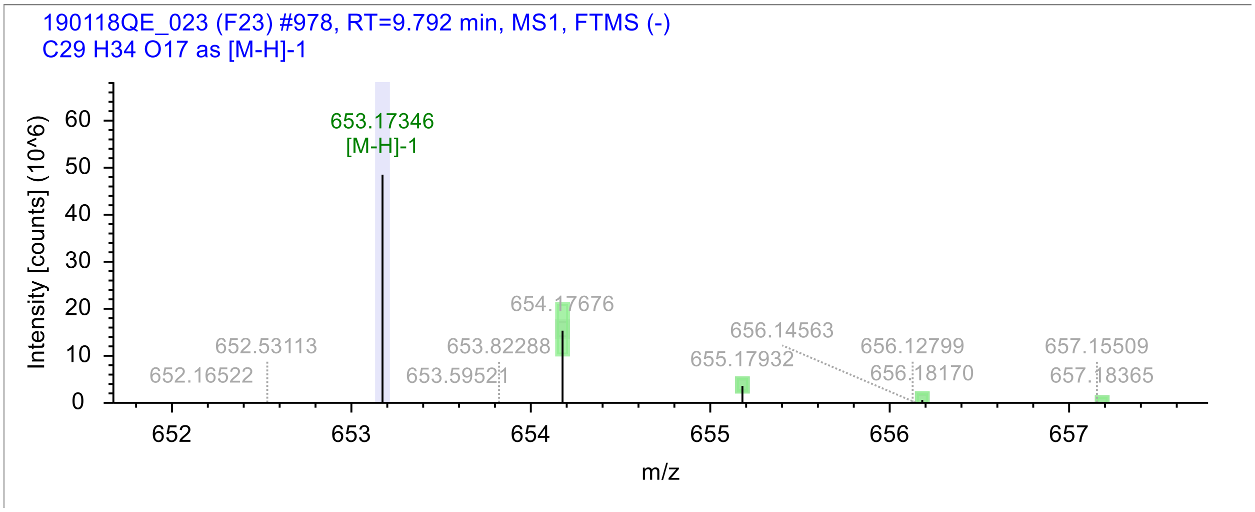

10/20 eV

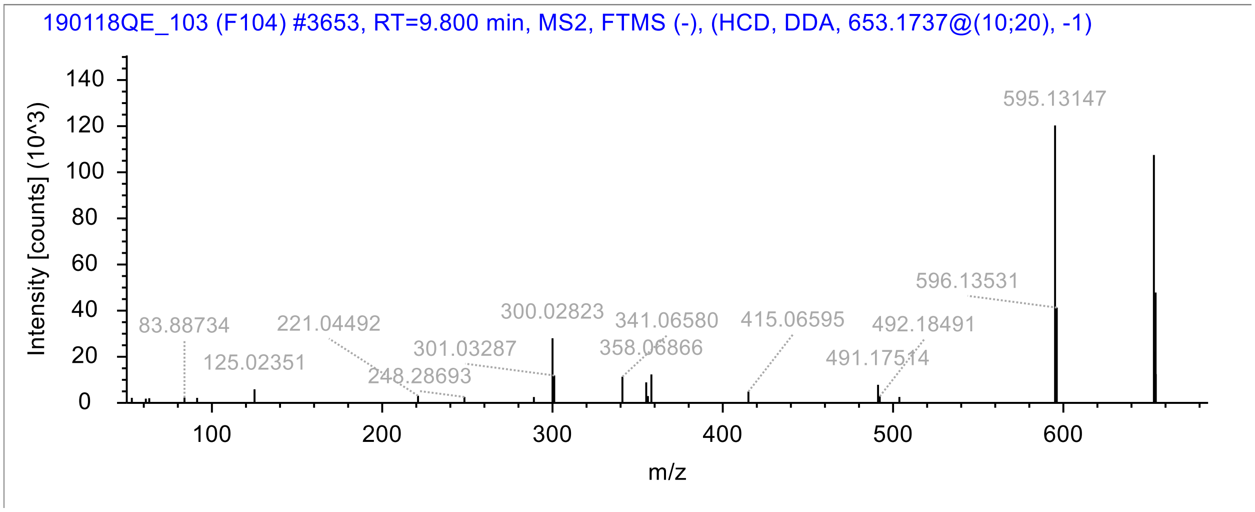

50/60 eV

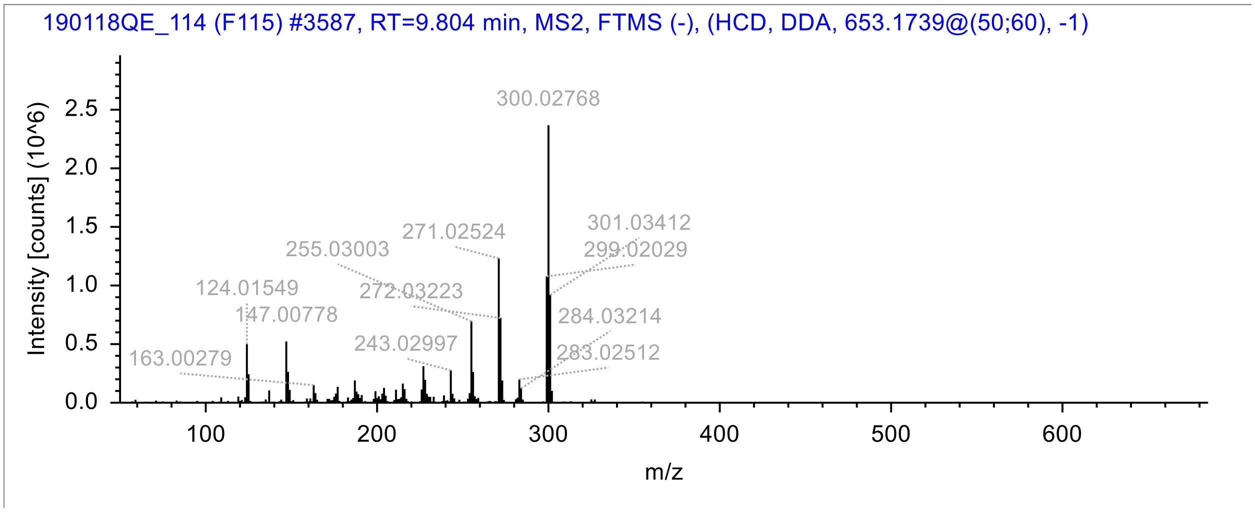

14. Delphinidin derivative	$C_{29}H_{34}O_{17}$	654.1811	10.26	2.36	3

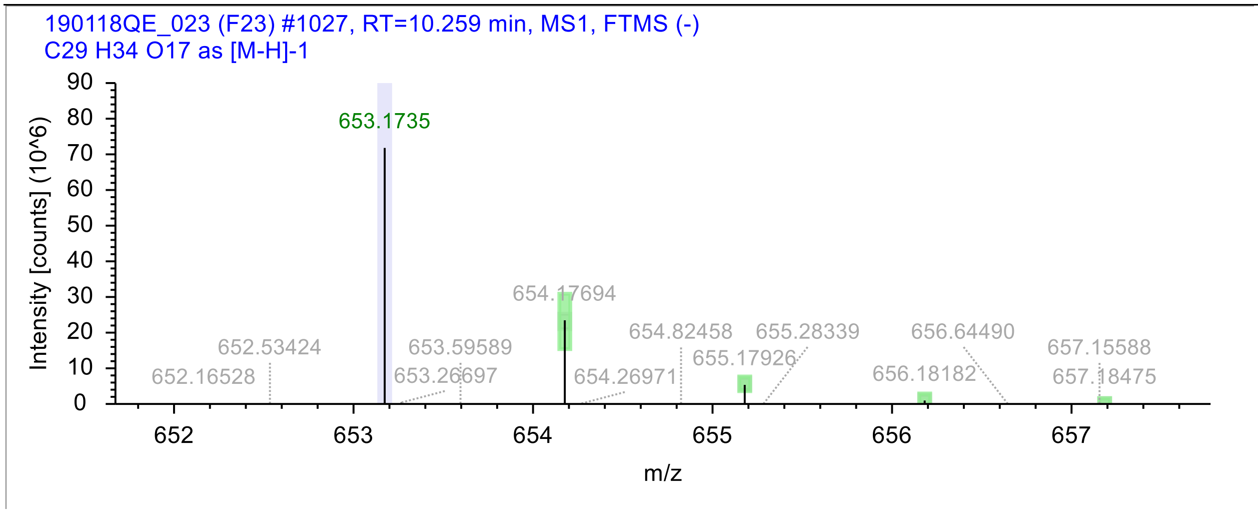

10/20 eV

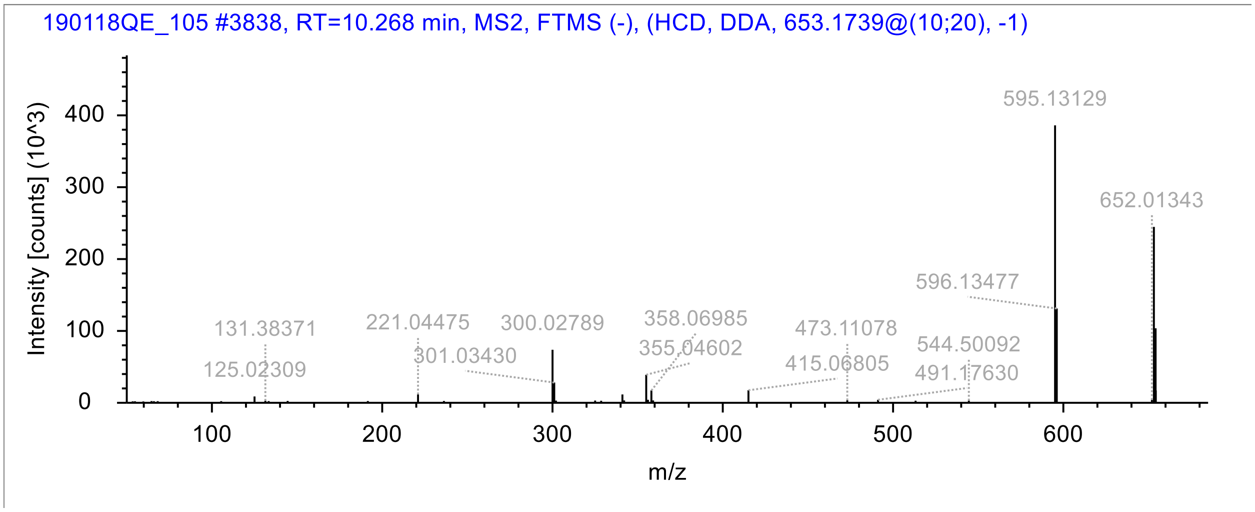

10/20 eV

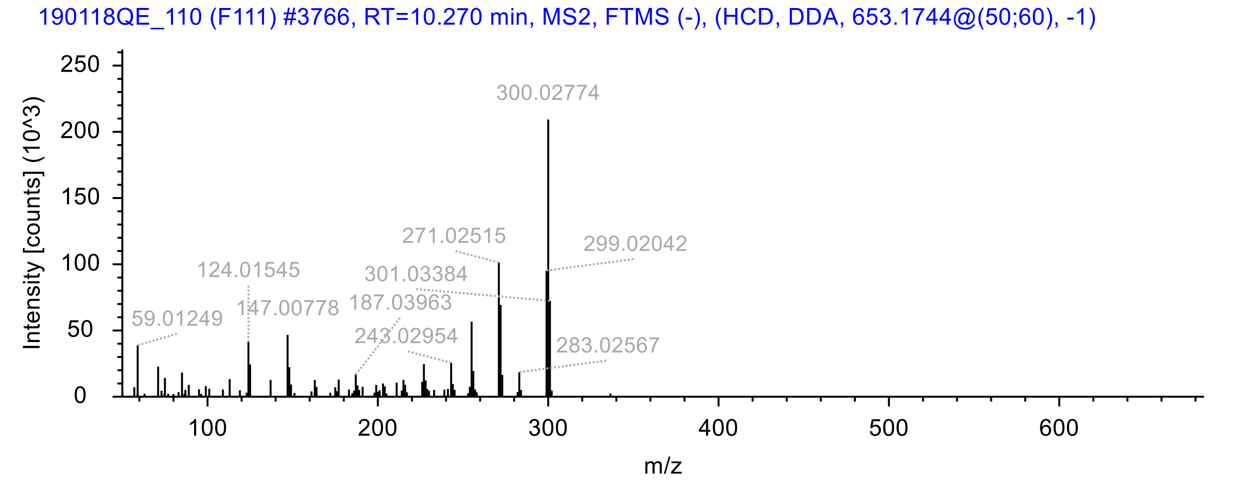

15. Dihydroxy methoxy benzoic acid gallate	$C_{15}H_{12}O_9$	336.0488	10.43	2.02	2/3

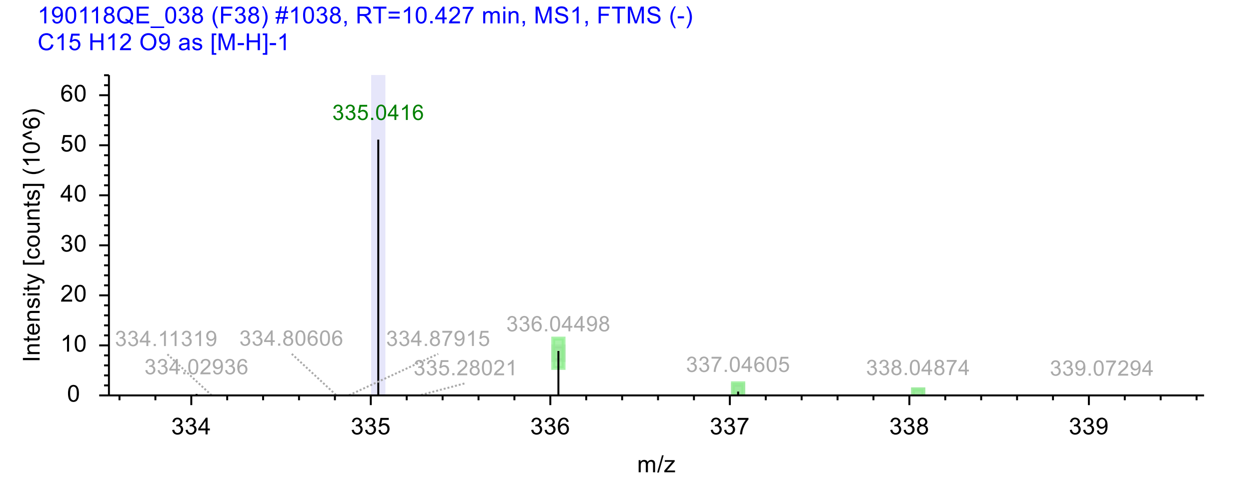

10/20 eV

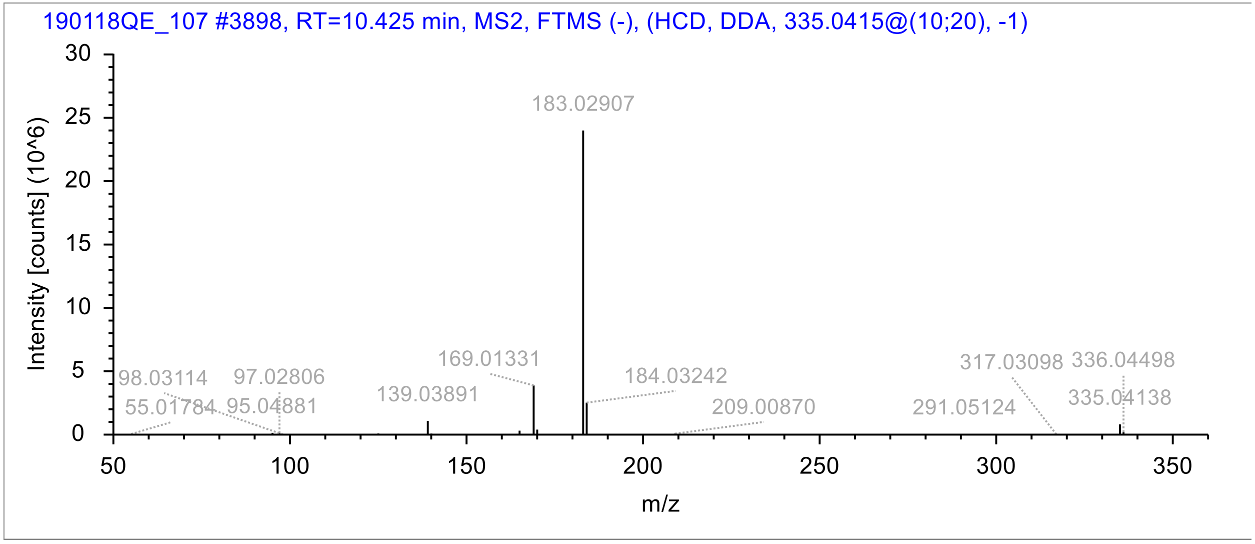

50/60 eV

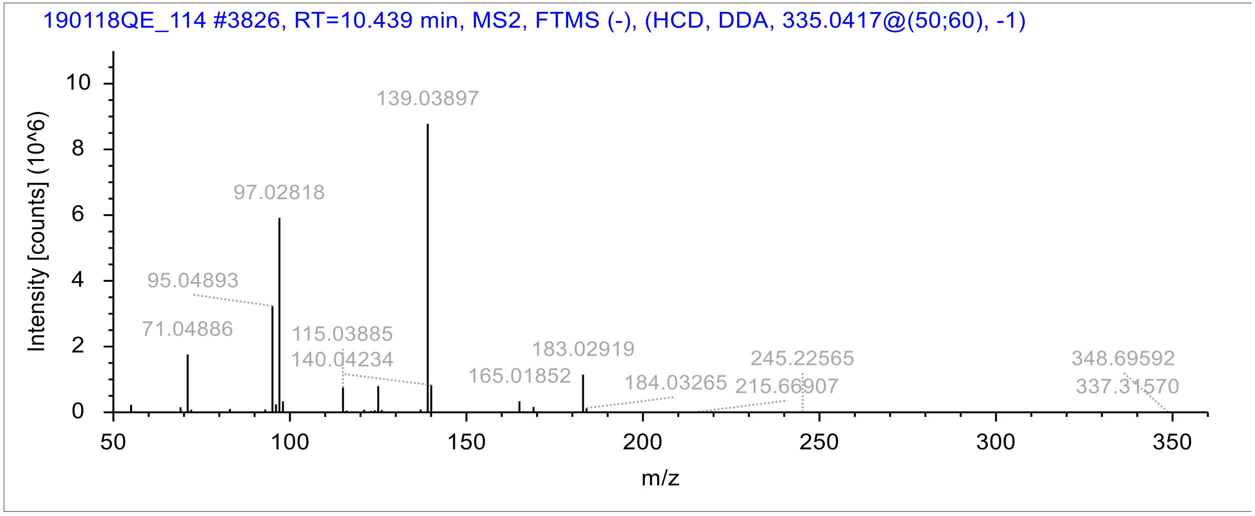

16. Dimethoxy hydroxybenzoic acid gallate	$C_{16}H_{14}O_9$	350.0645	12.10	1.94	2/3

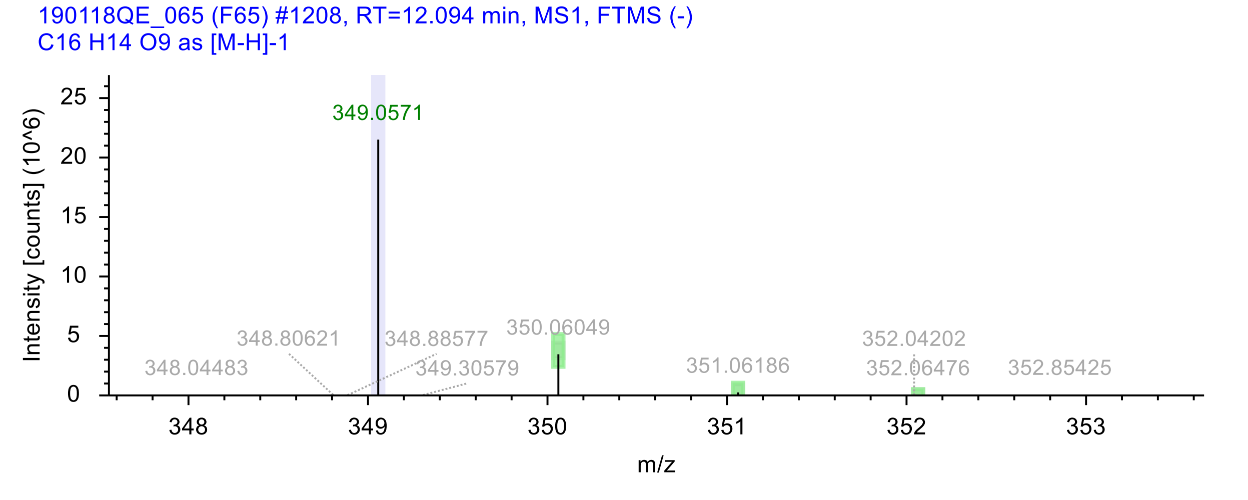

10/20 eV

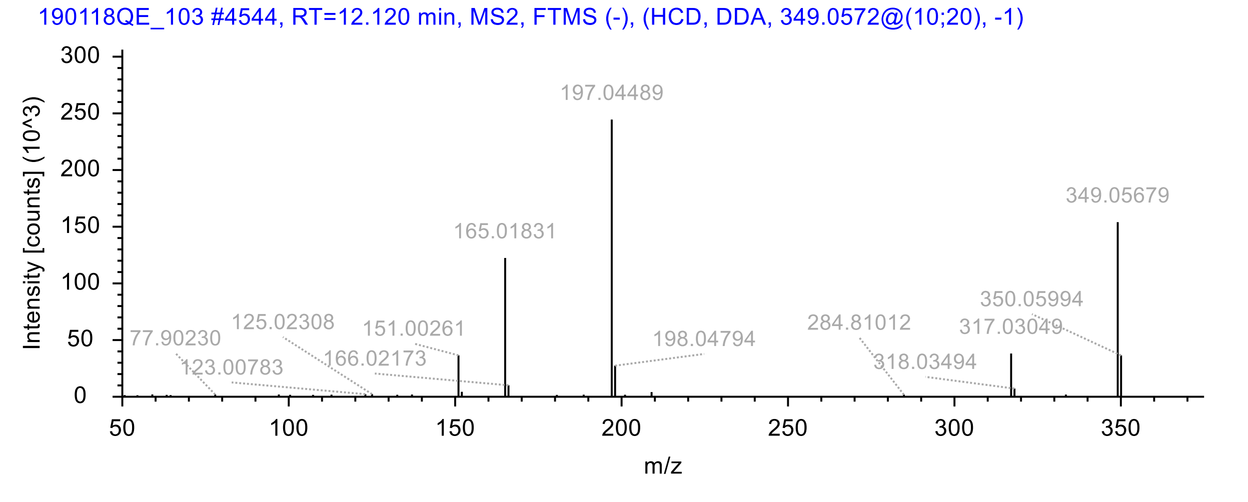

50/60 eV

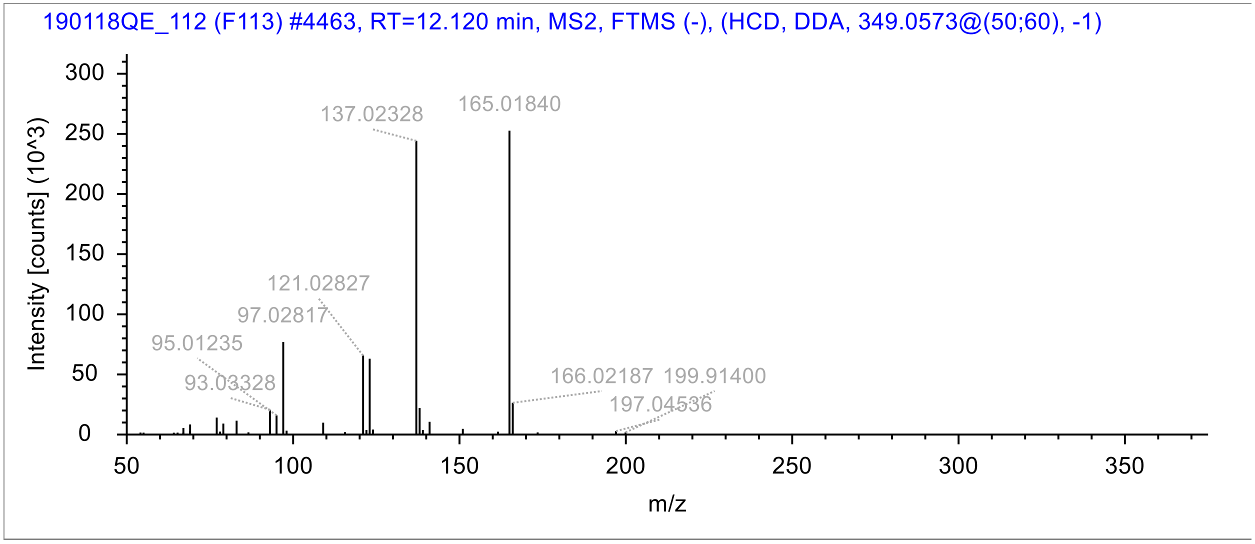

17. Hydroxy-tricetin hexoside pentoside	$C_{26}H_{28}O_{17}$	612.1342	12.68	2.47	2/3

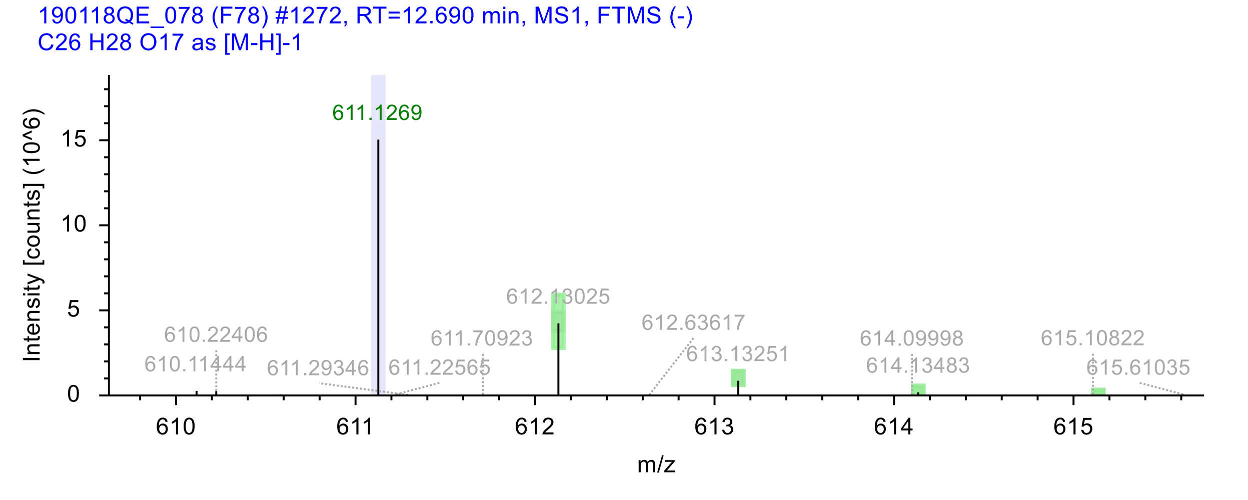

10/20 eV

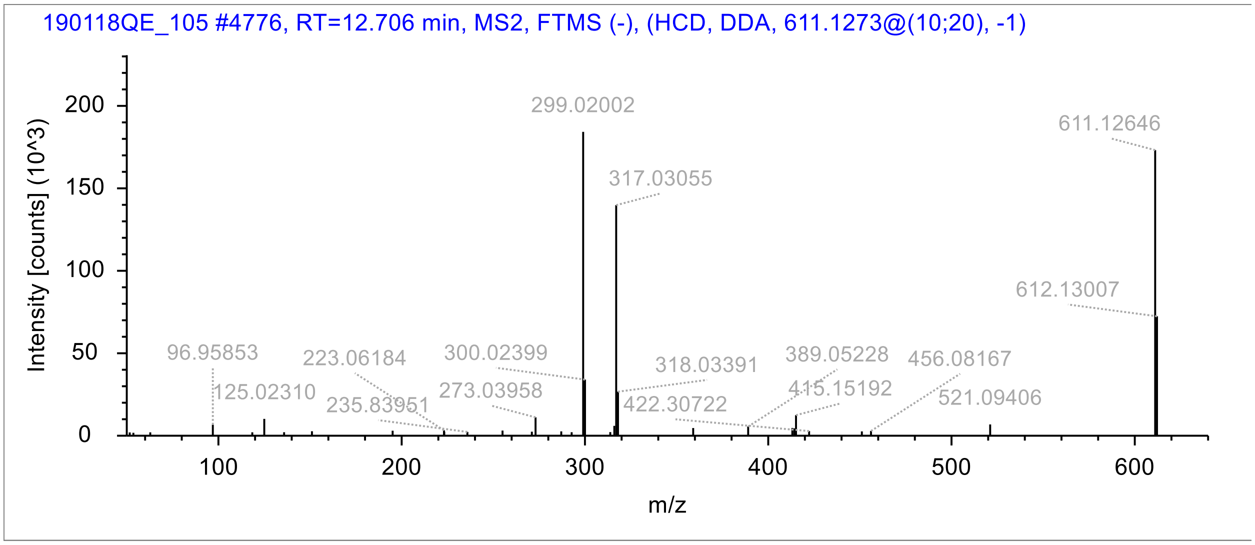

50/60 eV

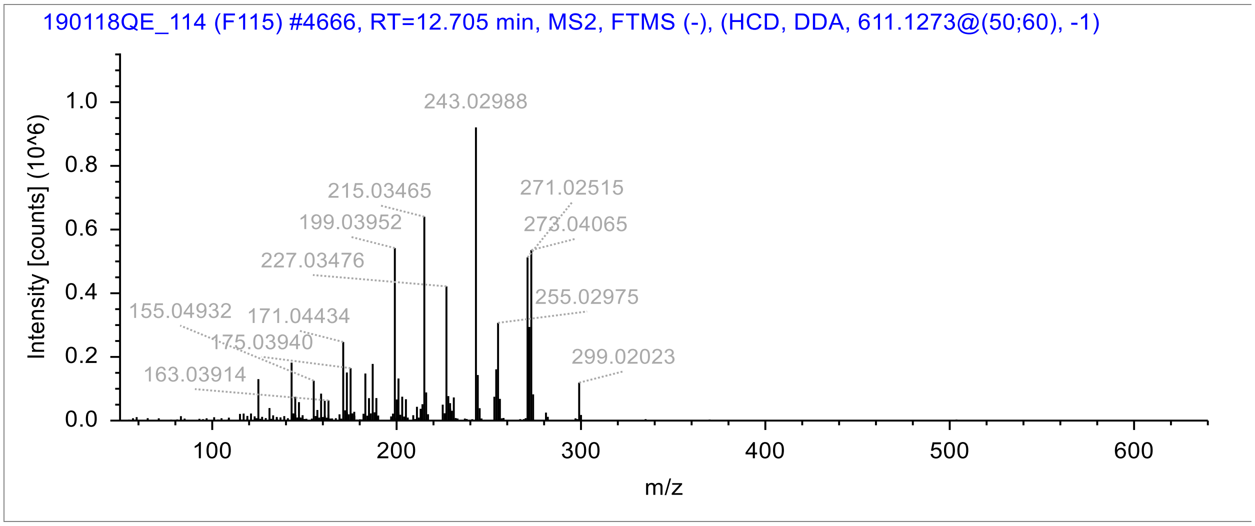

18. Gallic acid pentoside hexoside	$C_{21}H_{20}O_{12}$	464.0961	12.87	1.26	2/3

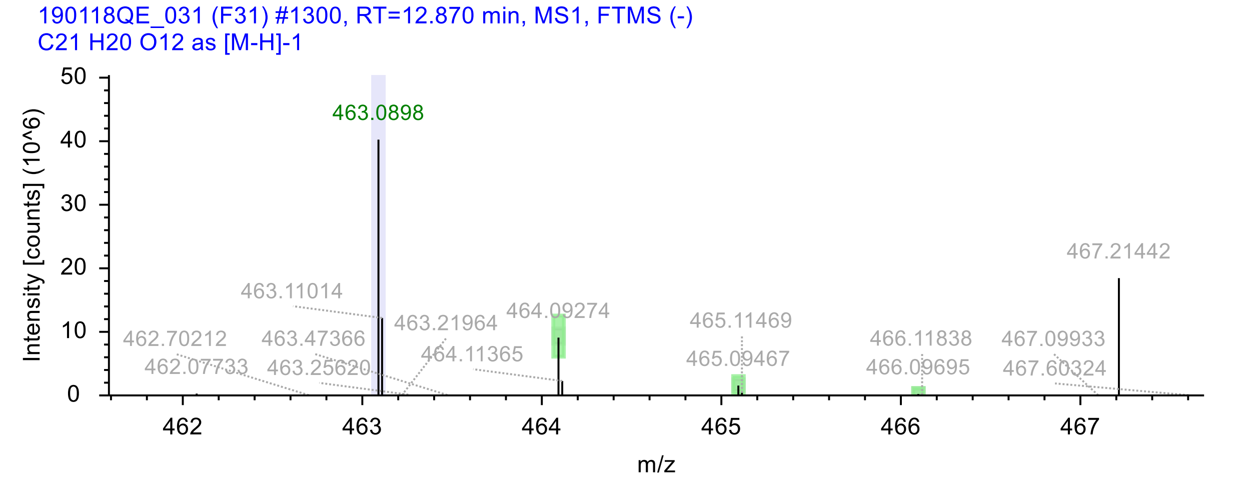

10/20 eV

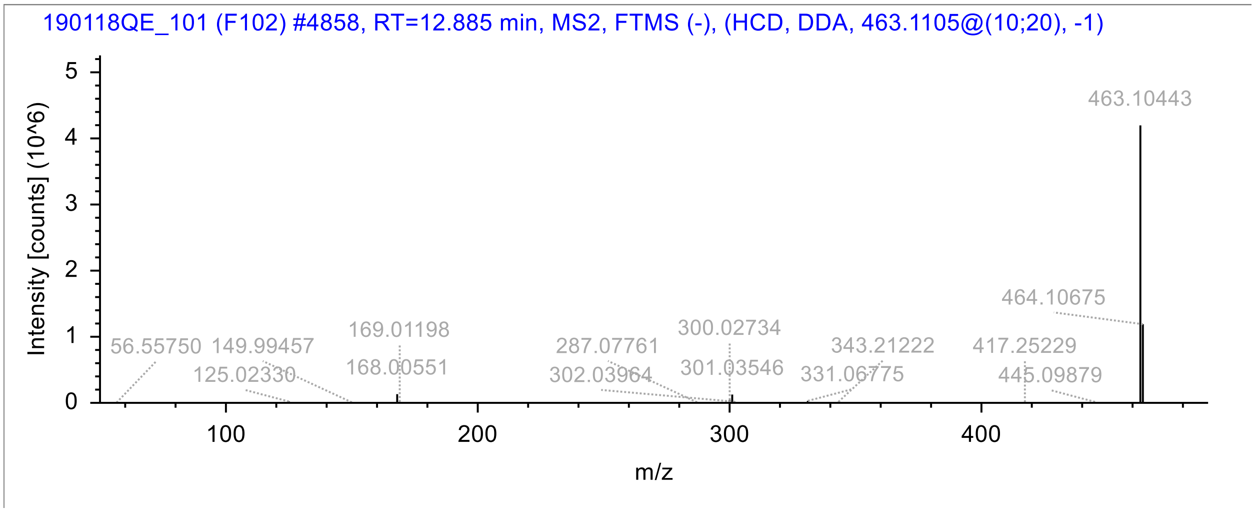

50/60 eV

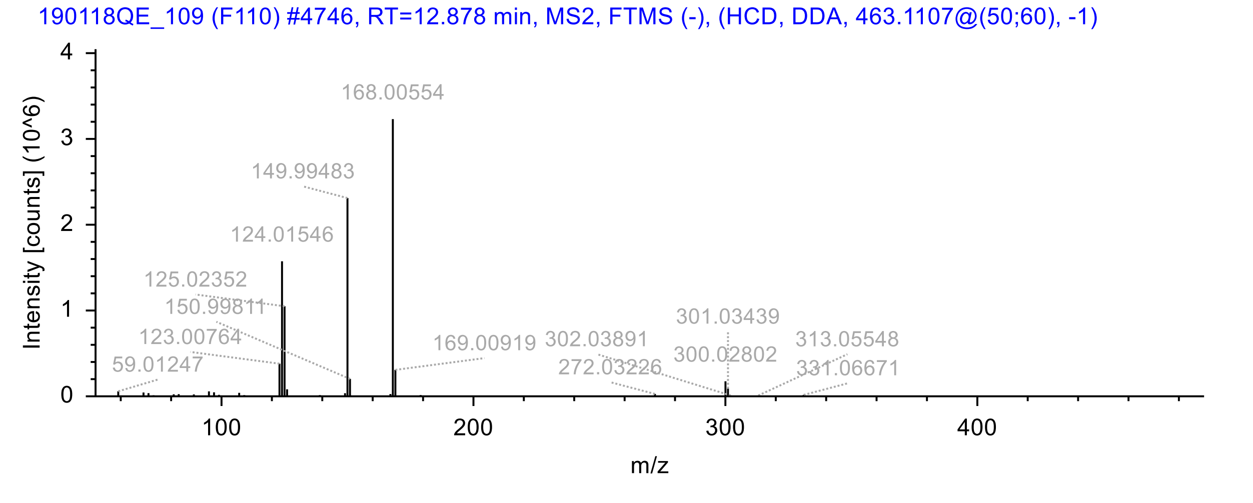

19. Tricetin pentoside	$C_{20}H_{18}O_{11}$	434.0857	13.53	1.86	2/3

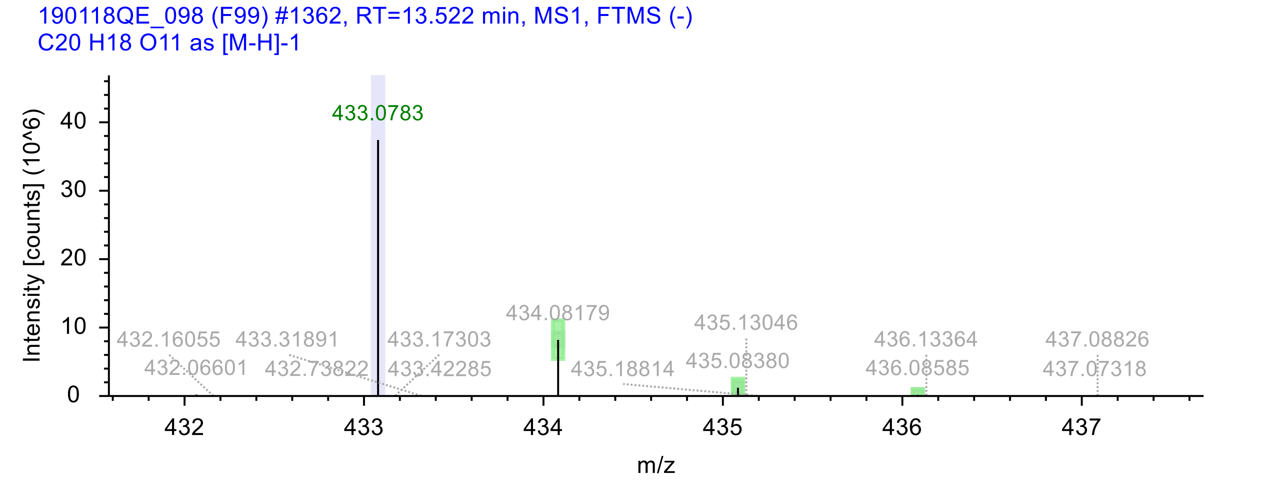

10/20 eV

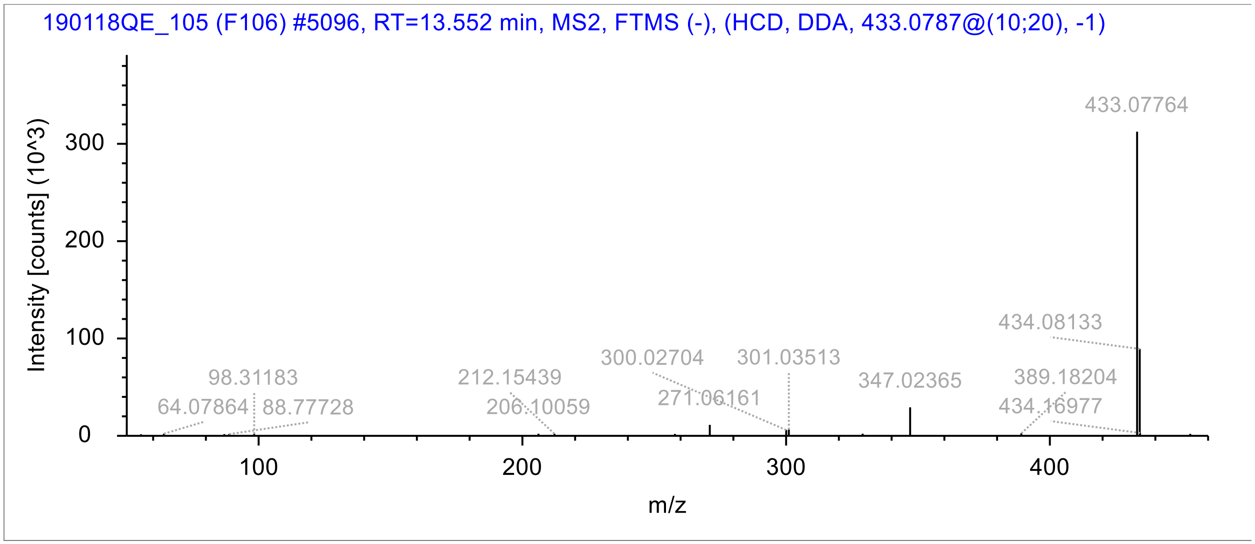

50/60 eV

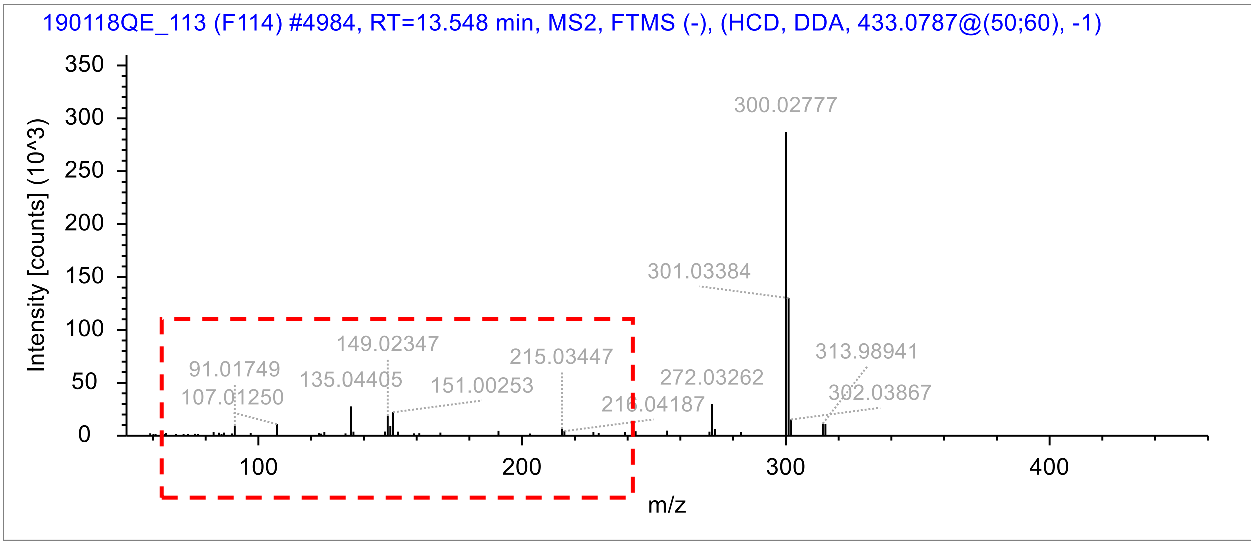

50/60 eV (zoomed-in)

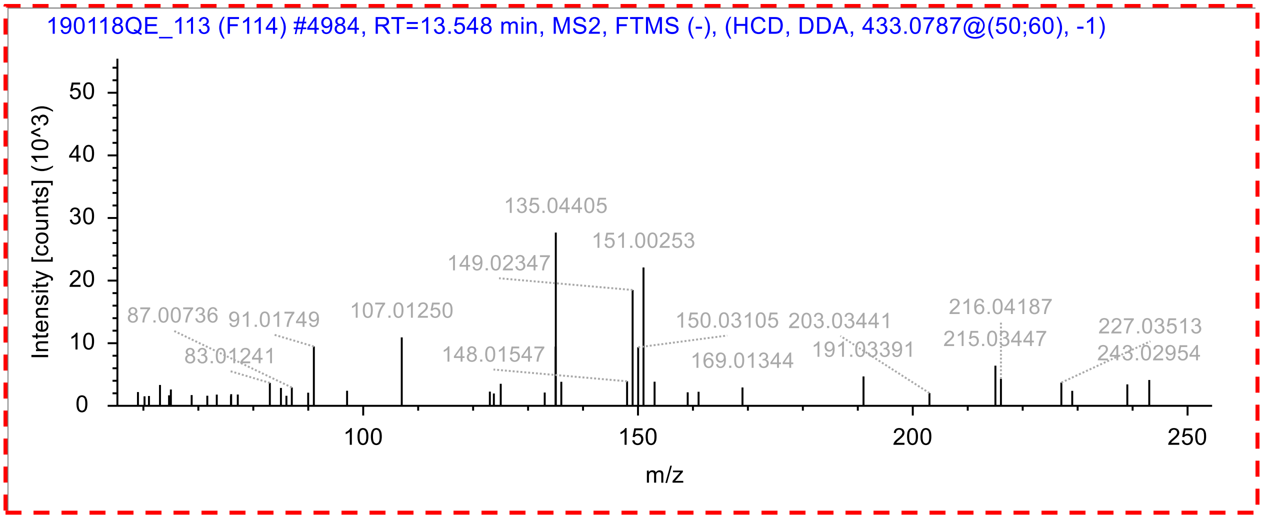

20. Luteolin hexoside	$C_{21}H_{20}O_{11}$	448.1009	14.36	0.85	2/3

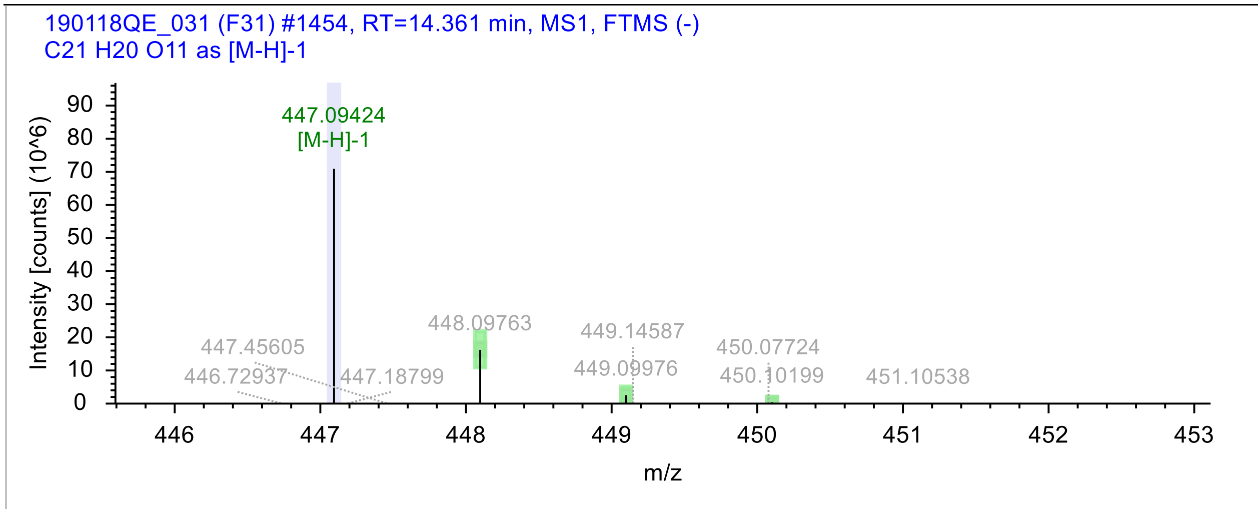

10/20 eV

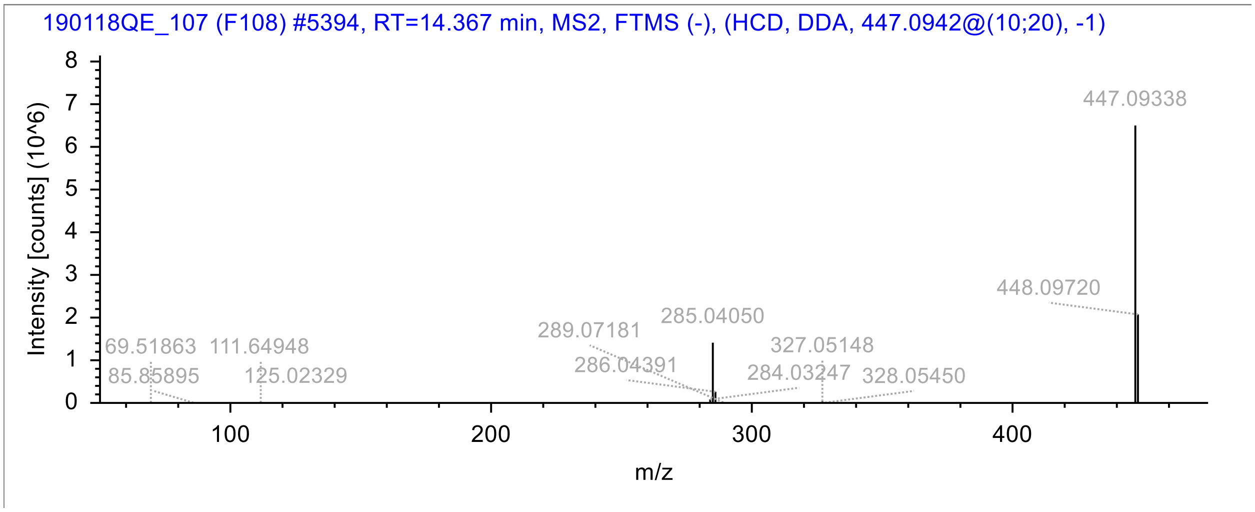

50/60 eV

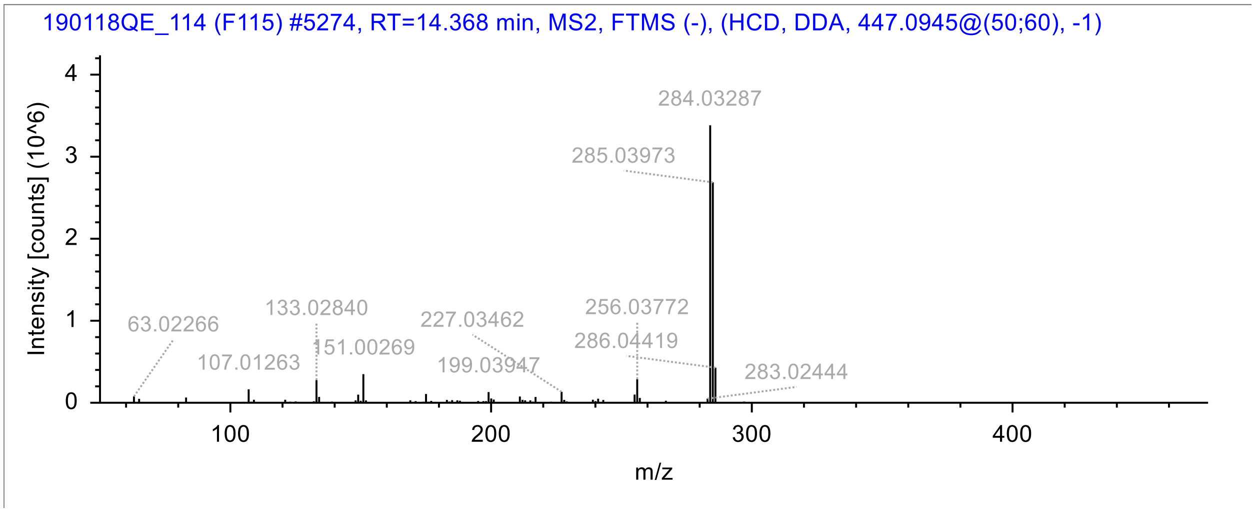

21. Tricetin hexoside malonyl hexoside	$C_{30}H_{32}O_{20}$	712.1509	14.64	3.04	2/3

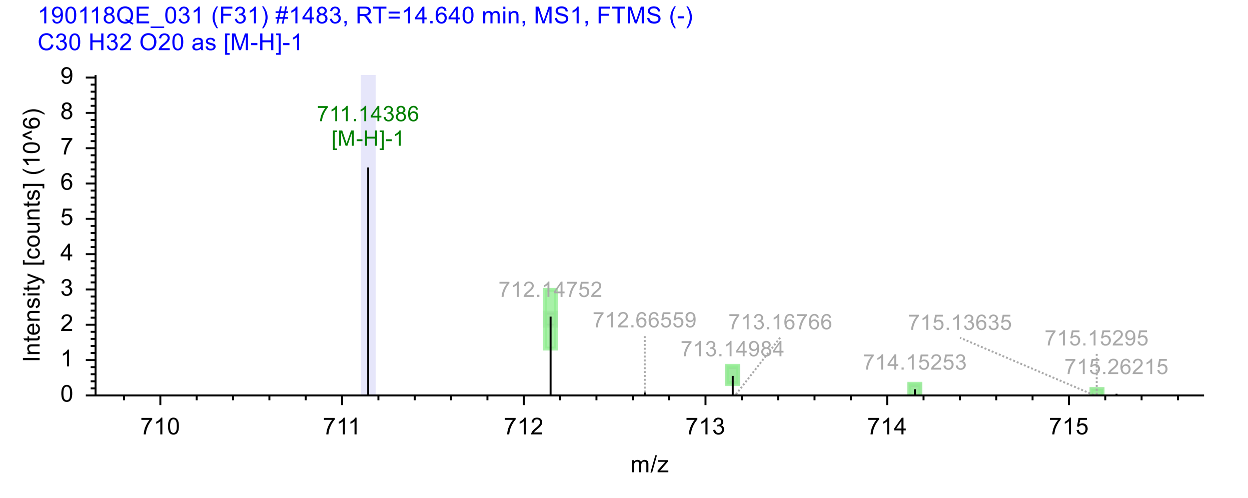

10/20 eV

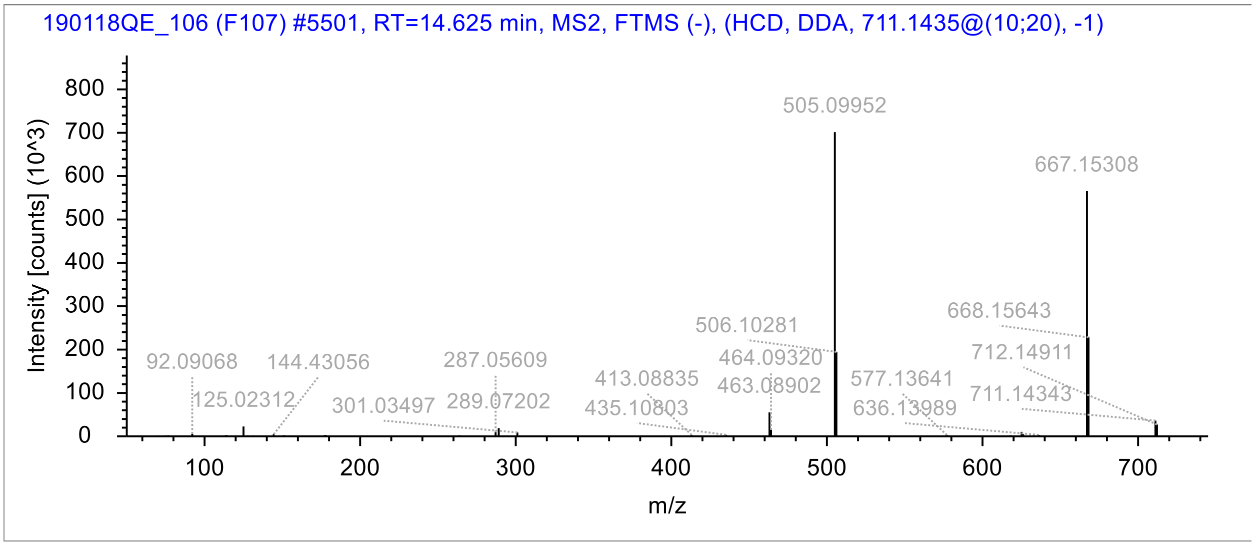

50/60 eV

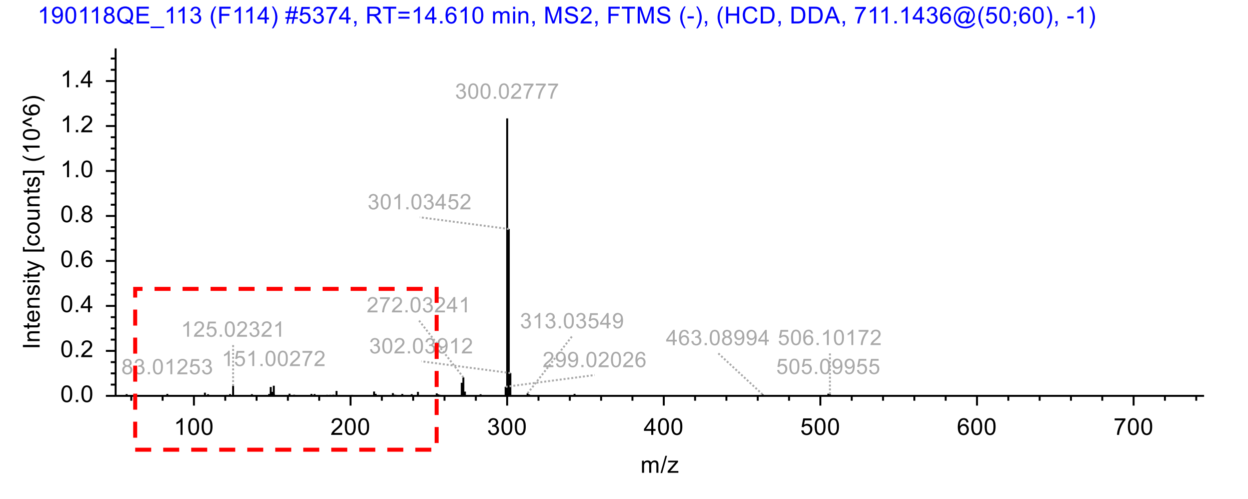

50/60 eV (zoomed-in)

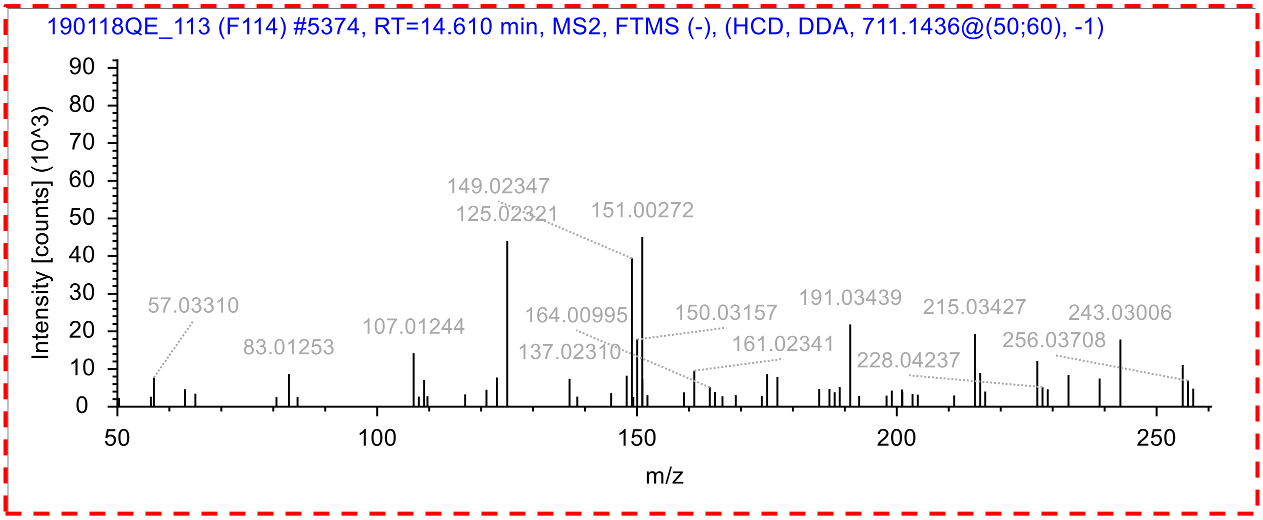

22. Tricetin pentoside malonyl hexoside	$C_{29}H_{30}O_{19}$	682.1397	15.07	2.25	2/3

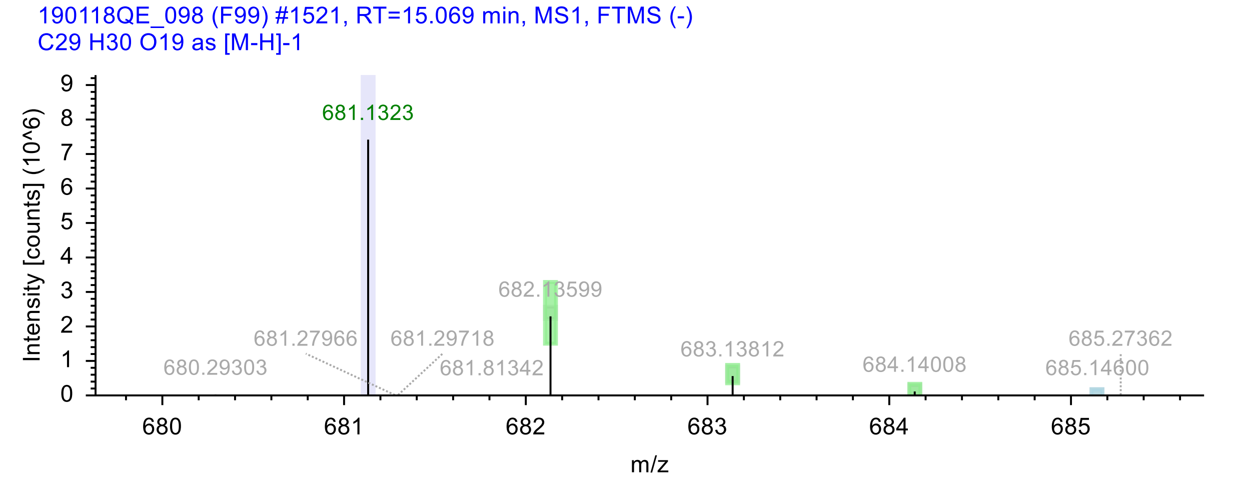

10/20 eV

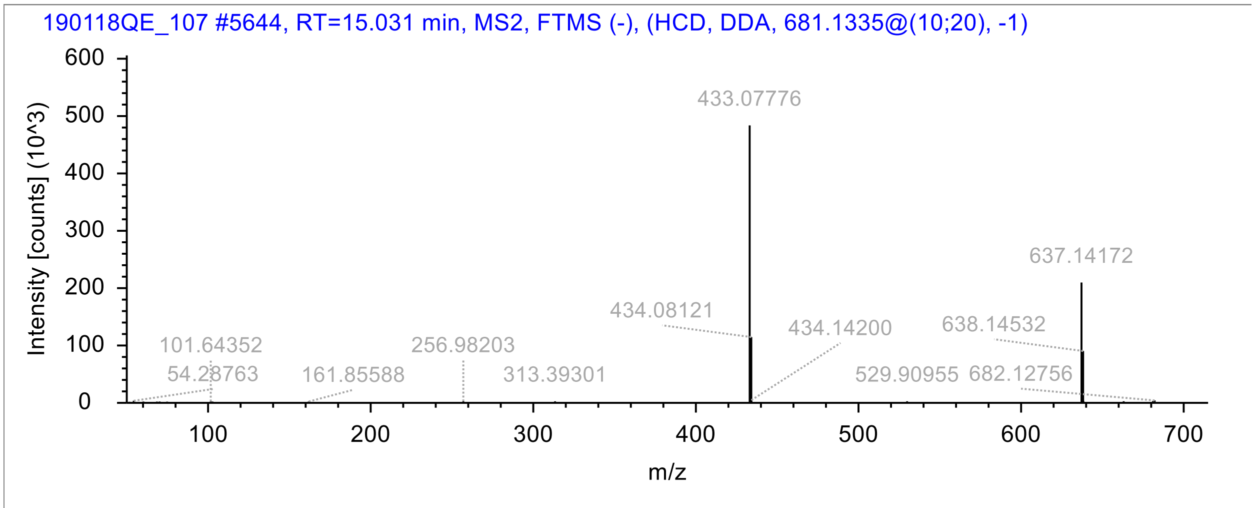

50/60 eV

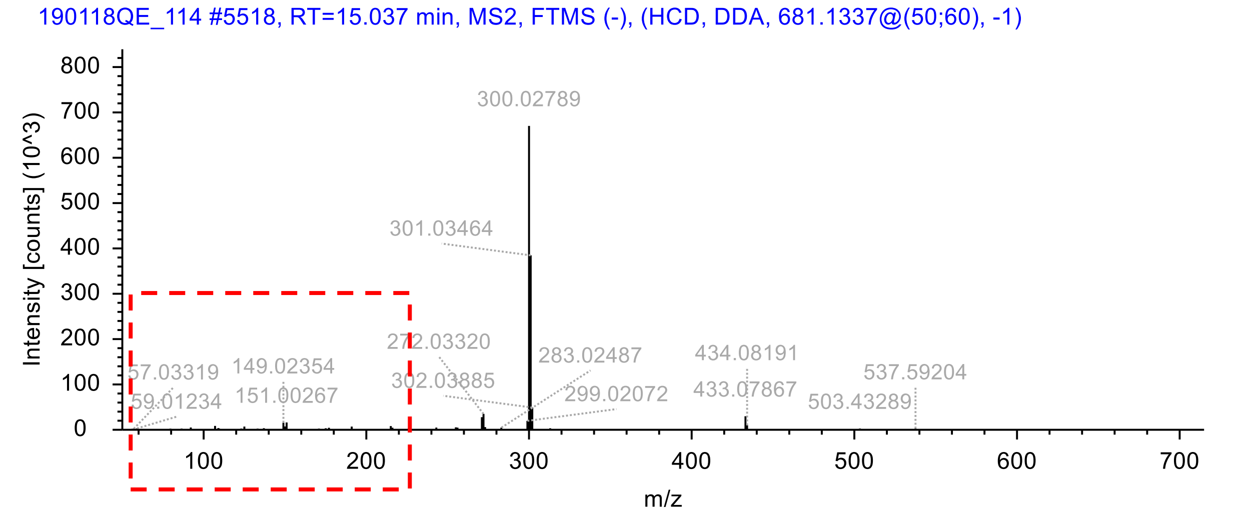

50/60 eV (zoomed-in)

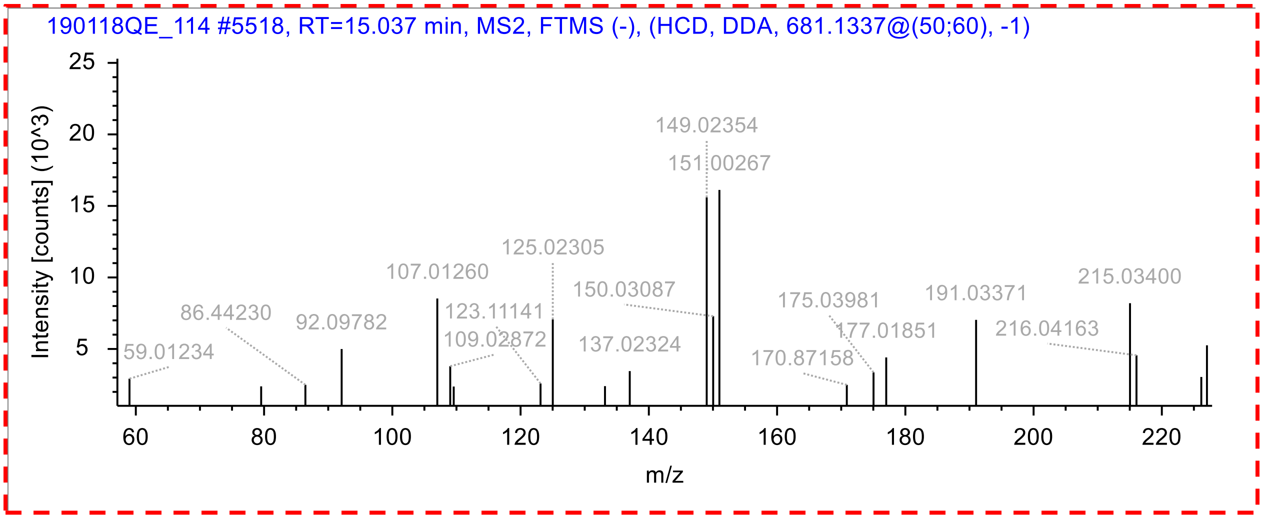

23. Luteolin pentoside	$C_{20}H_{18}O_{10}$	418.0910	15.12	2.31	2/3

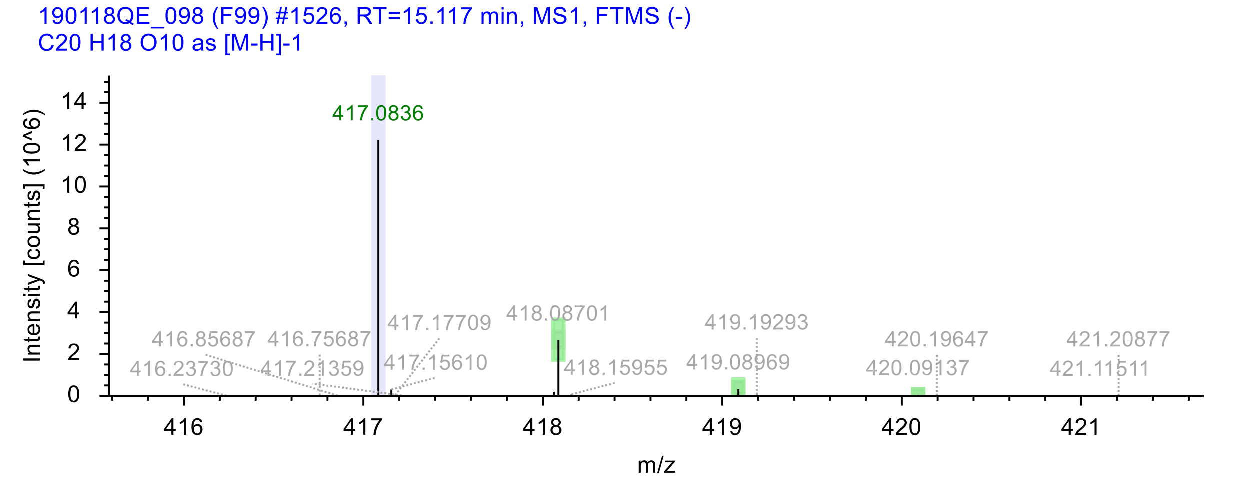

10/20 eV

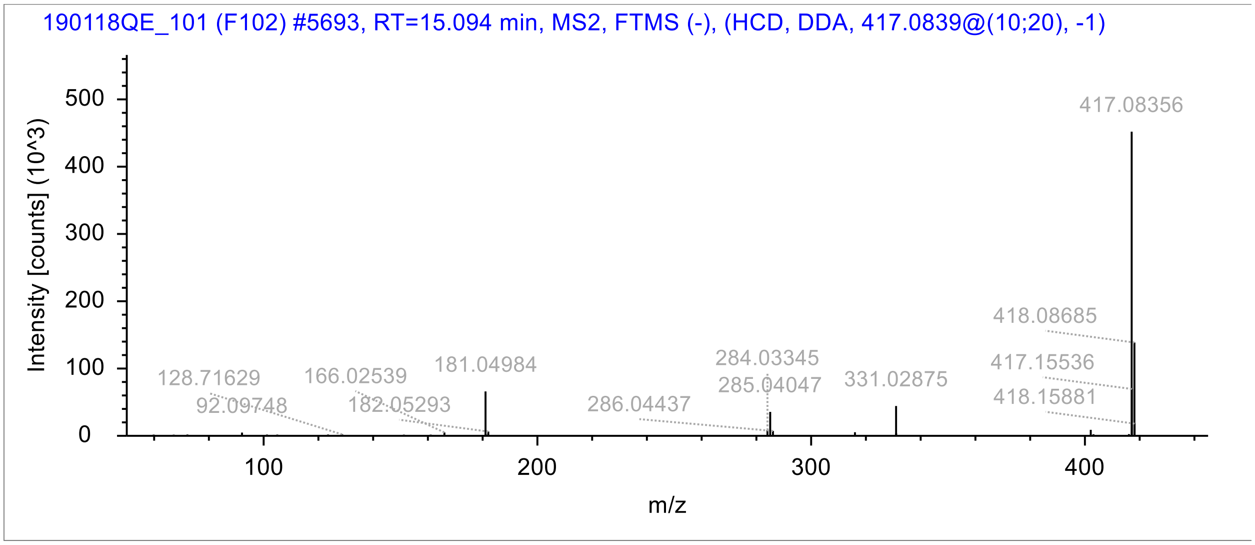

50/60 eV

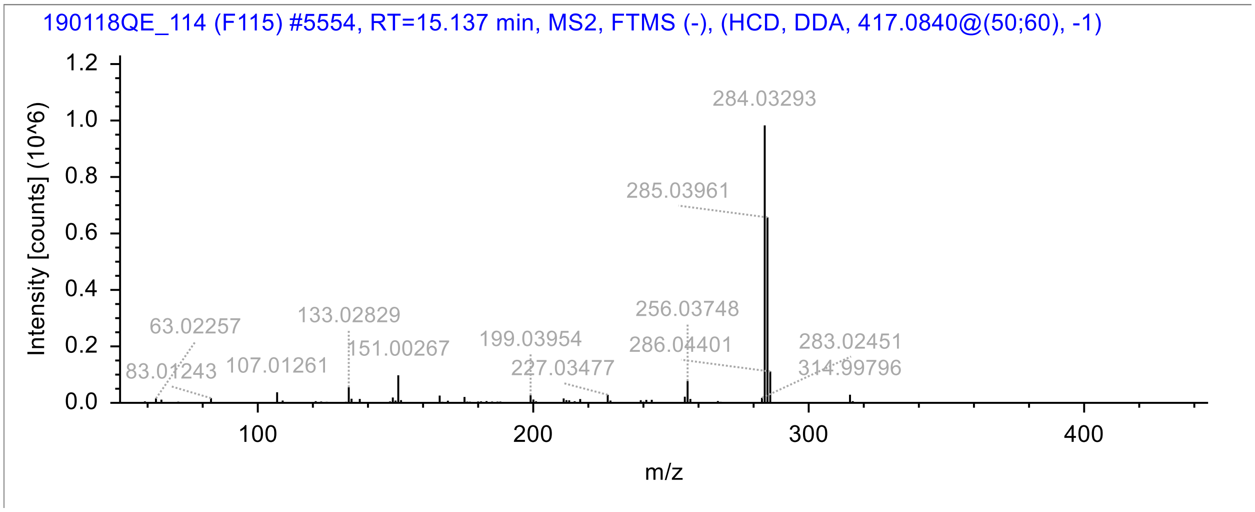

24. Tricetin malonyl hexoside isomer	$C_{24}H_{22}O_{15}$	550.0970	15.31	2.13	2/3

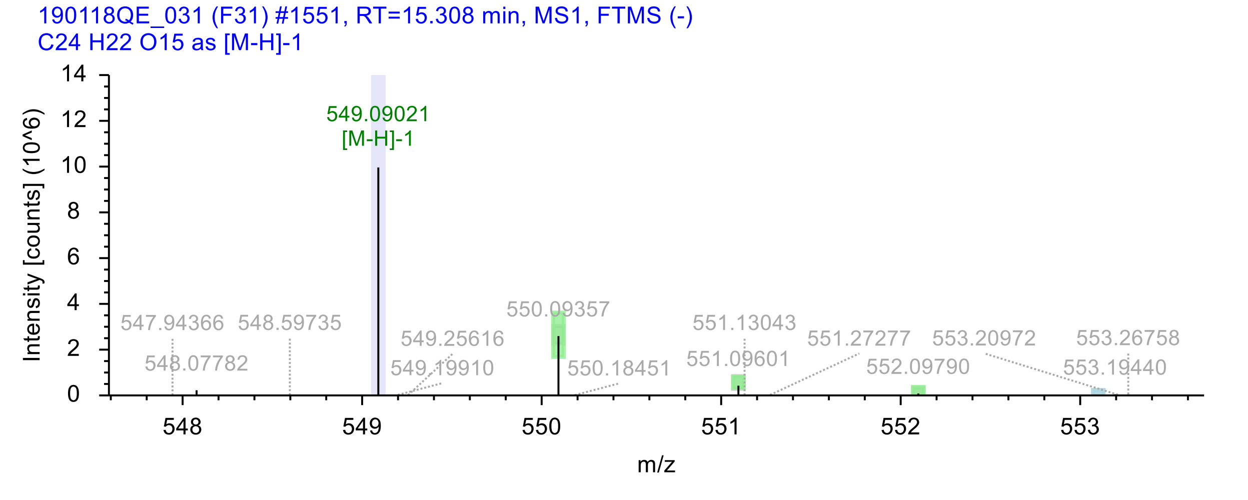

10/20 eV

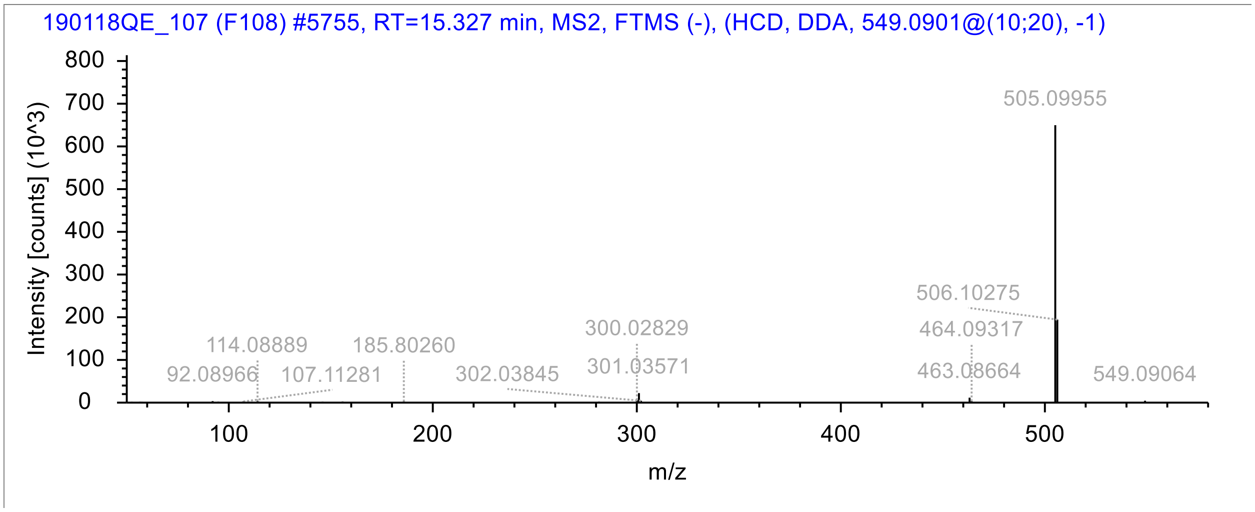

50/60 eV

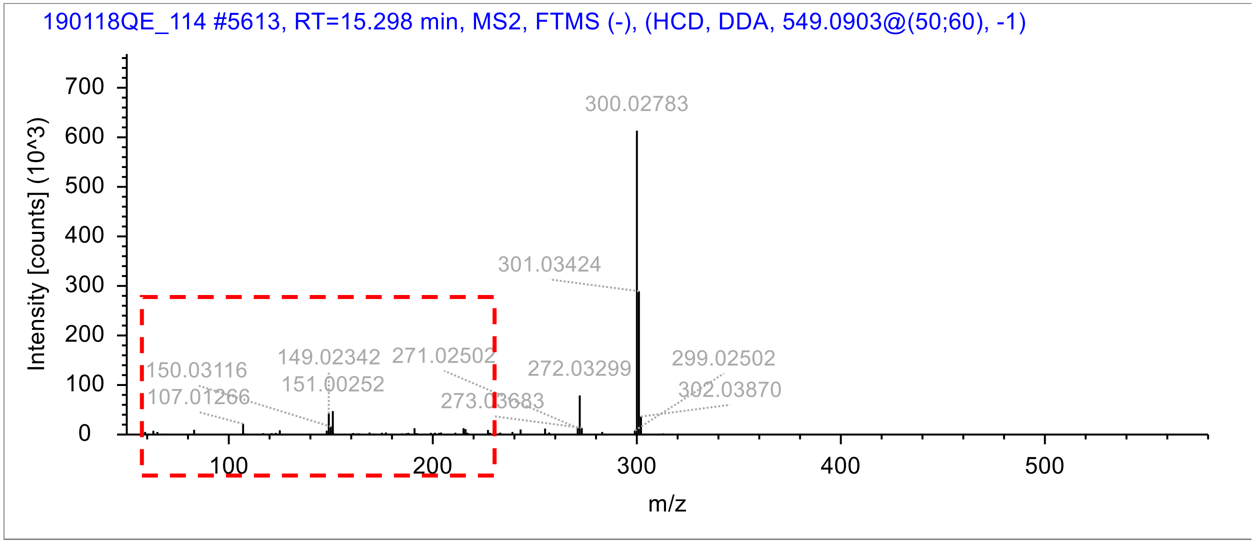

50/60 eV (zoomed-in)

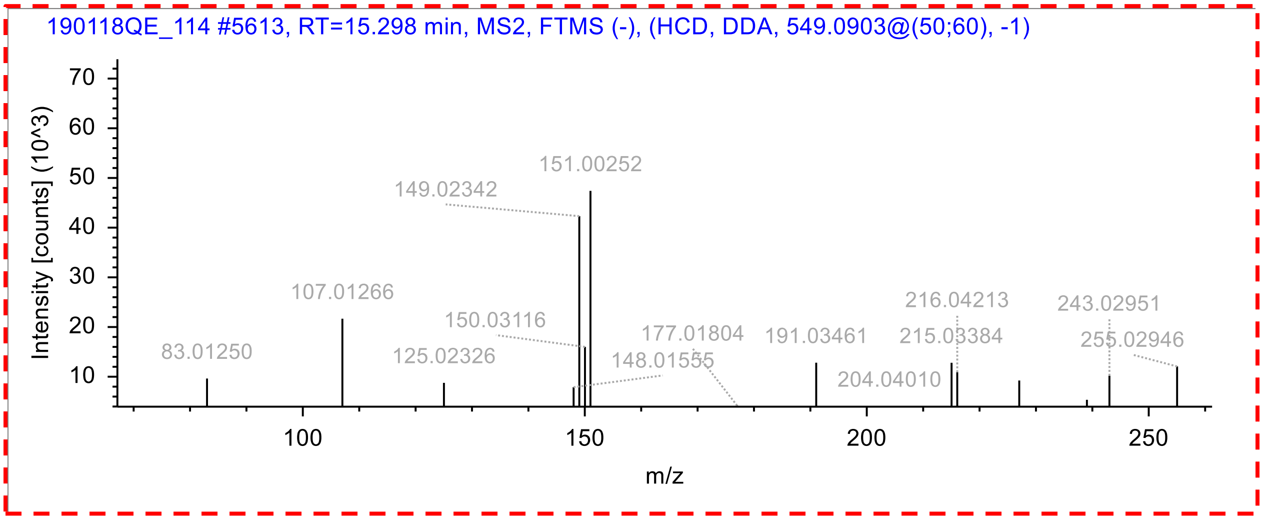

25. Tricetin hexoside	$C_{21}H_{20}O_{12}$	464.0961	15.42	1.23	2/3

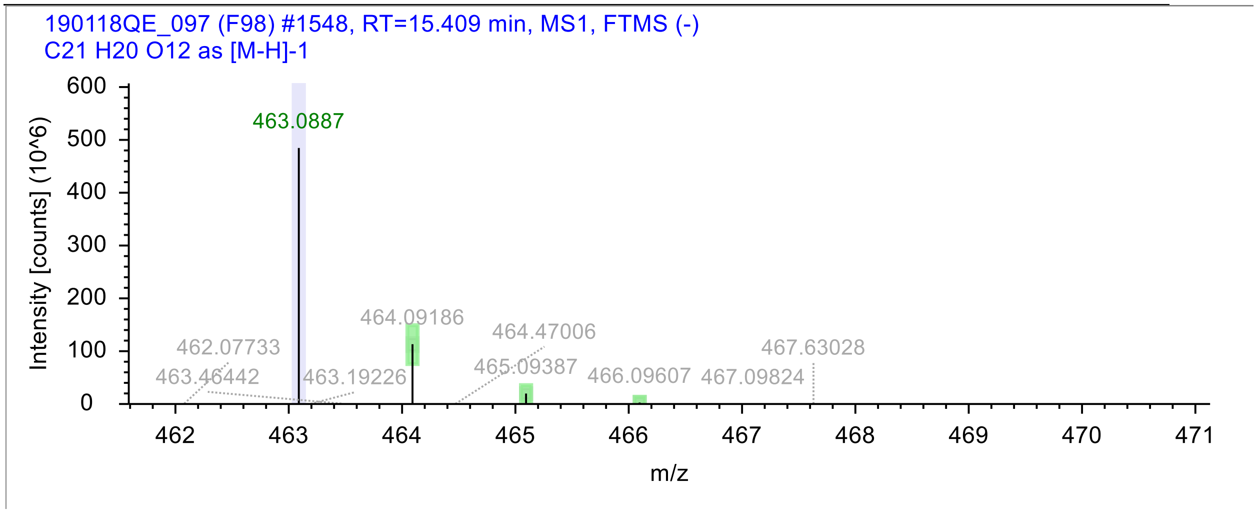

10/20 eV

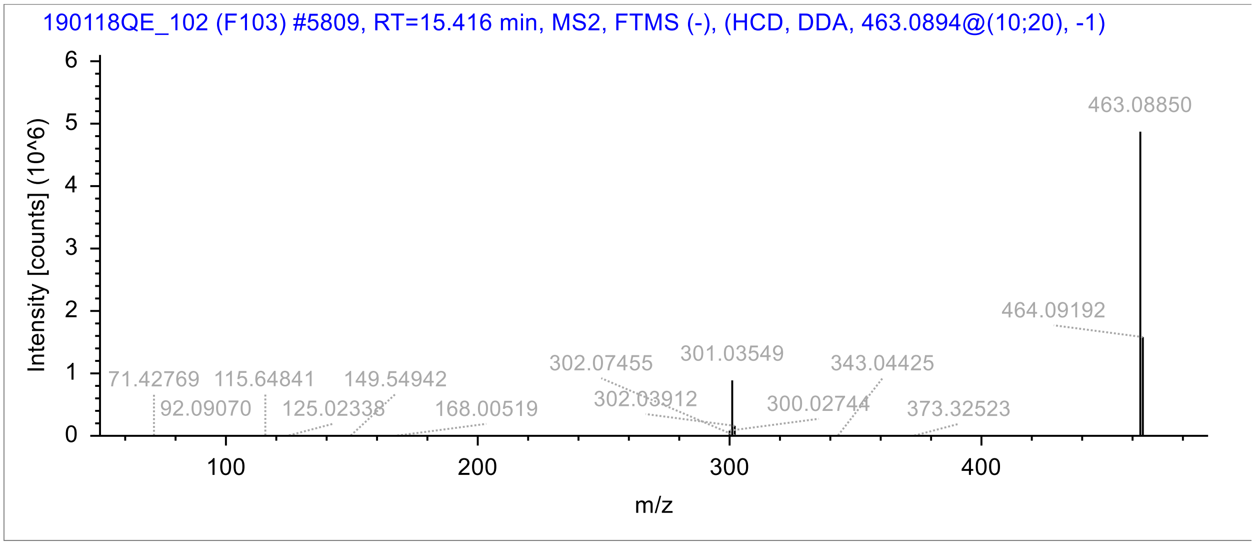

50/60 eV

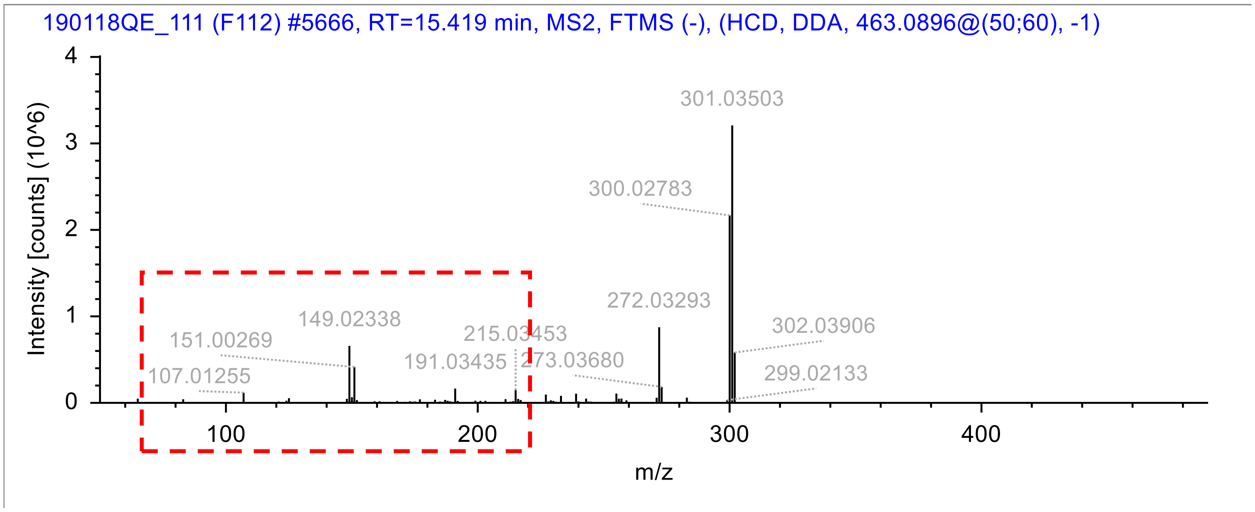

50/60 eV (zoomed-in)

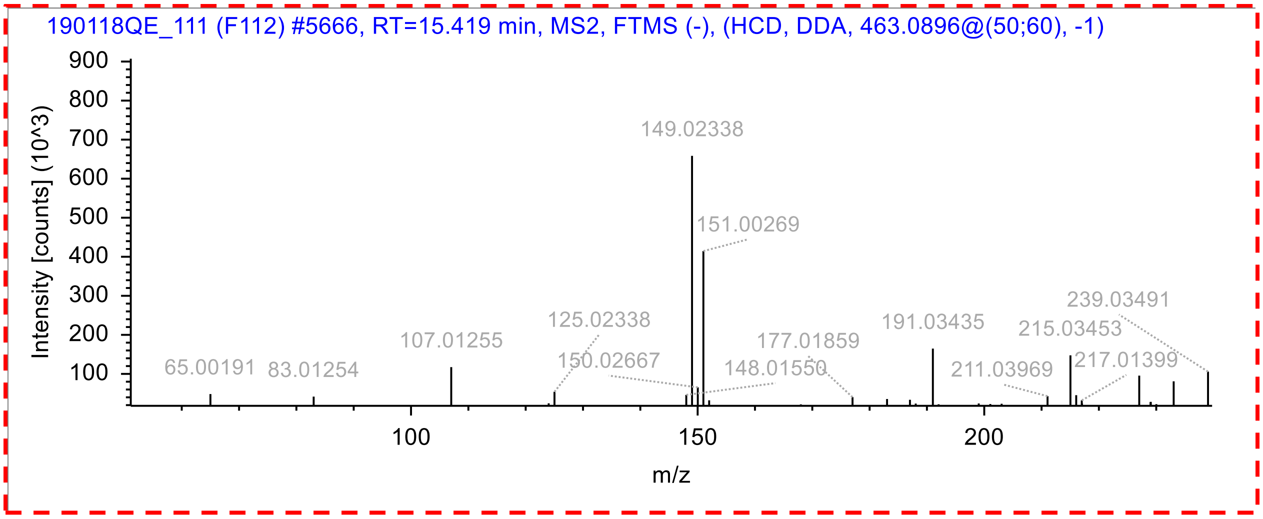

27. Luteolin 4'-O-glucoside	$C_{21}H_{20}O_{11}$	448.1010	16.37	0.93	1

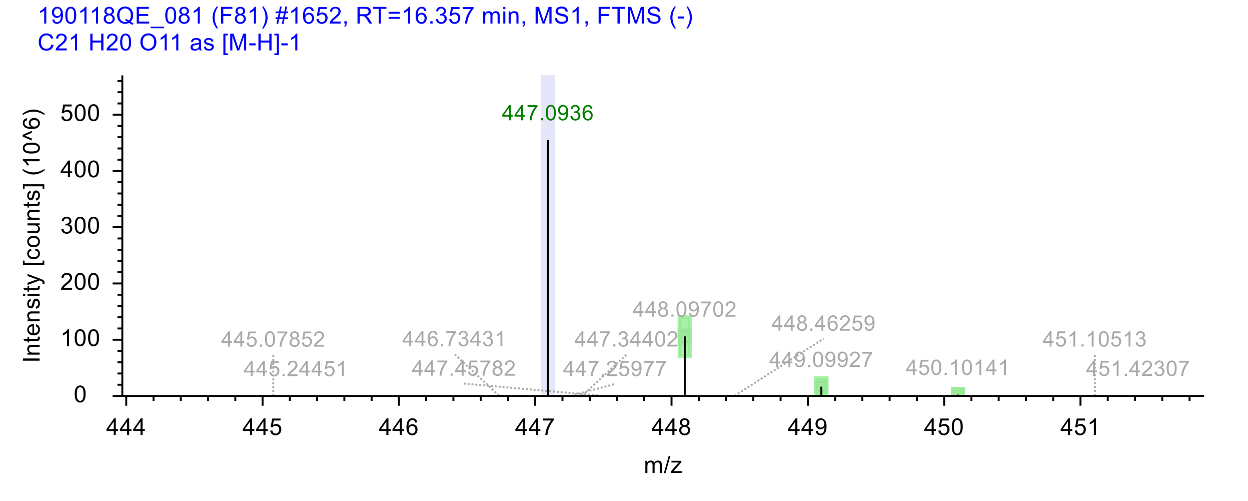

10/20 eV

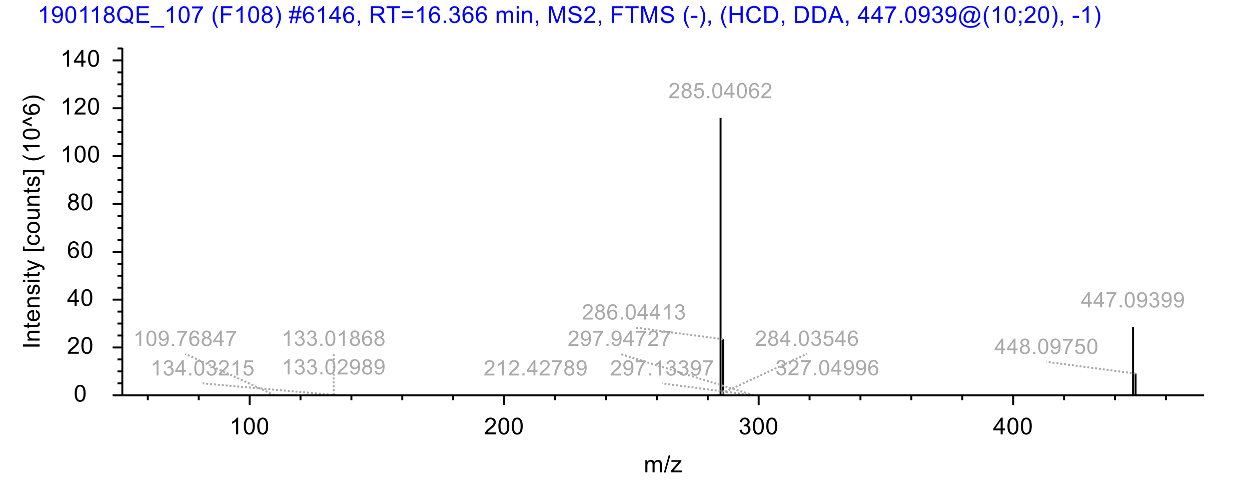

50/60 eV

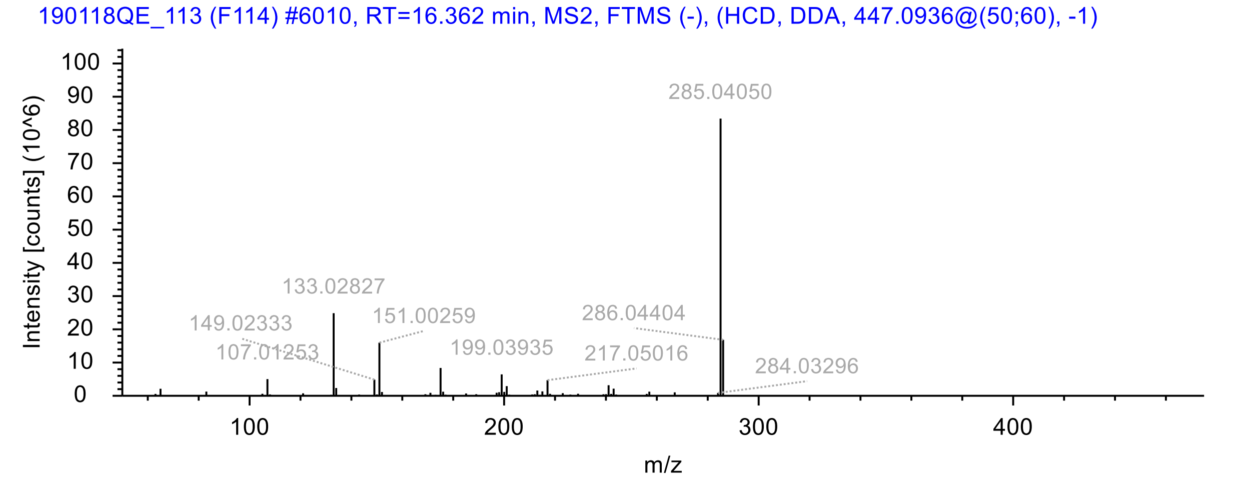

28. Luteolin malonyl hexoside	$C_{24}H_{22}O_{14}$	534.1019	16.63	1.83	2/3

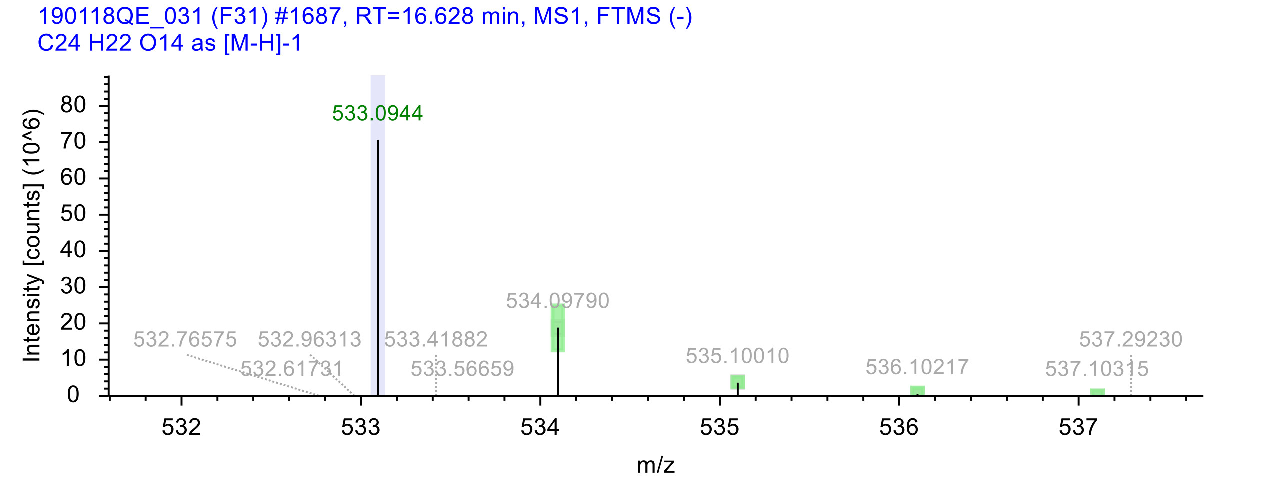

10/20 eV

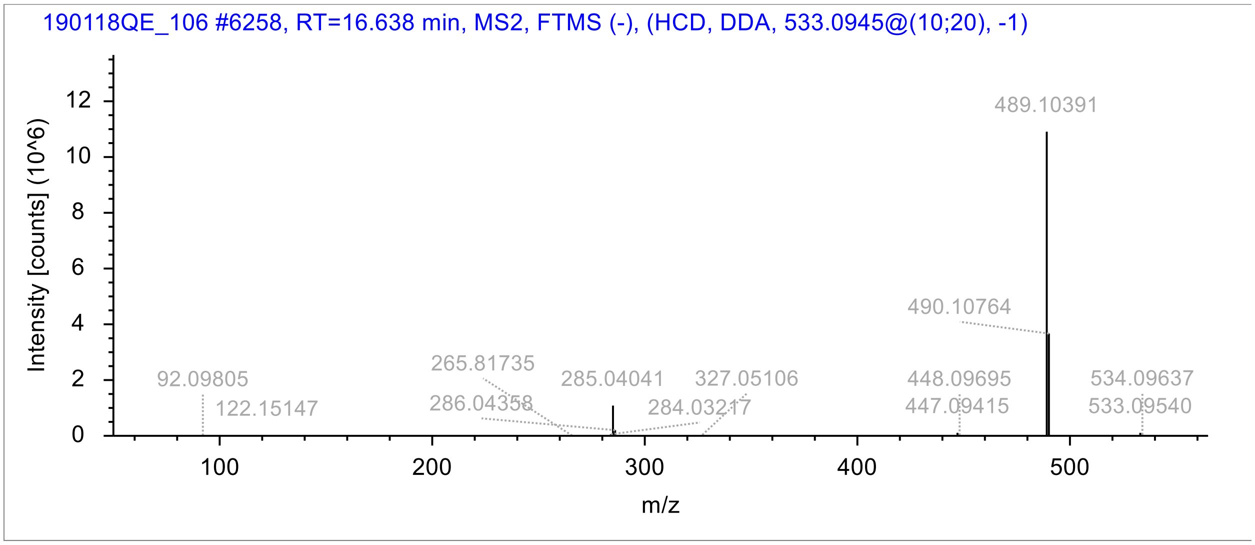

50/60 eV

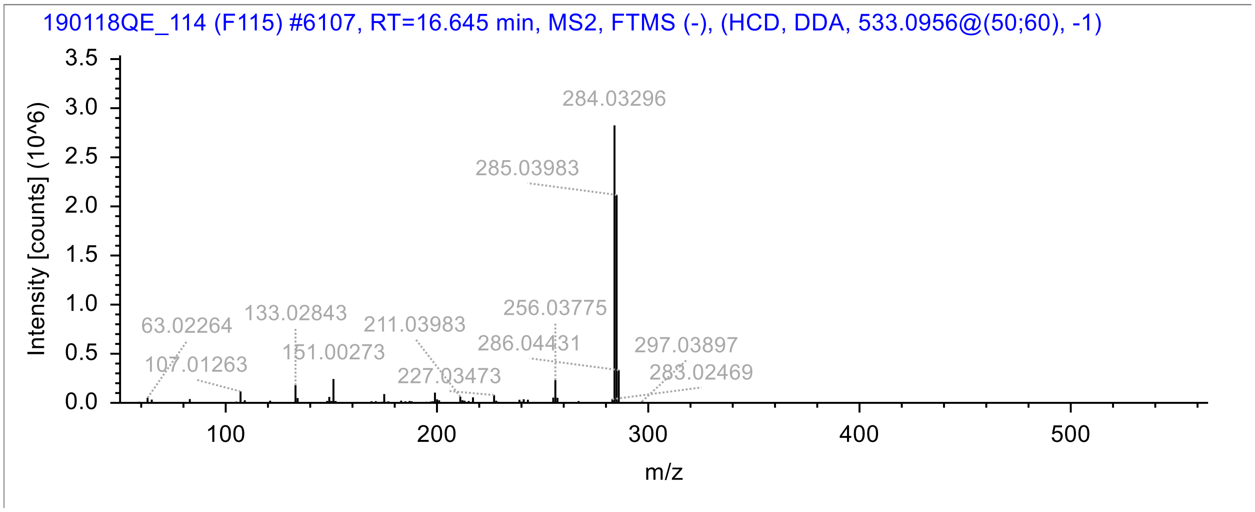

29. Tricetin malonyl hexoside	$C_{24}H_{22}O_{15}$	550.0970	16.78	2.12	2/3

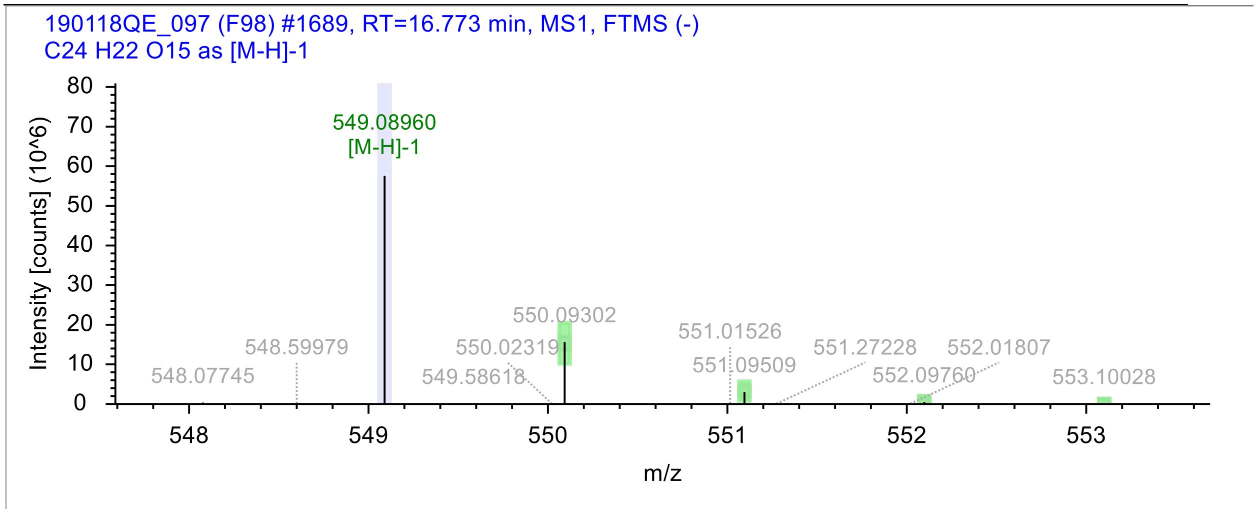

10/20 eV

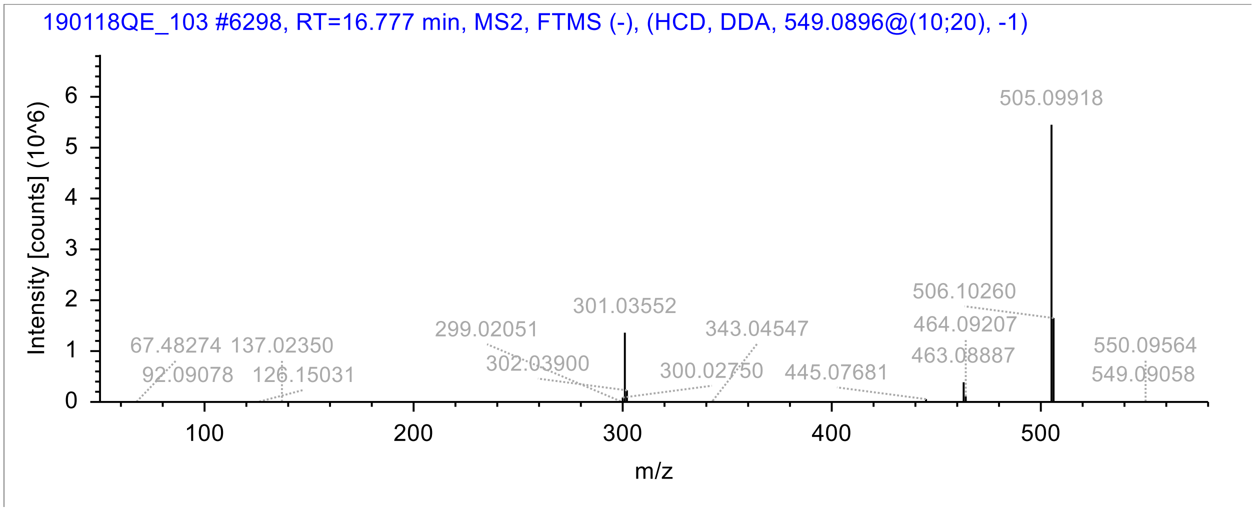

50/60 eV

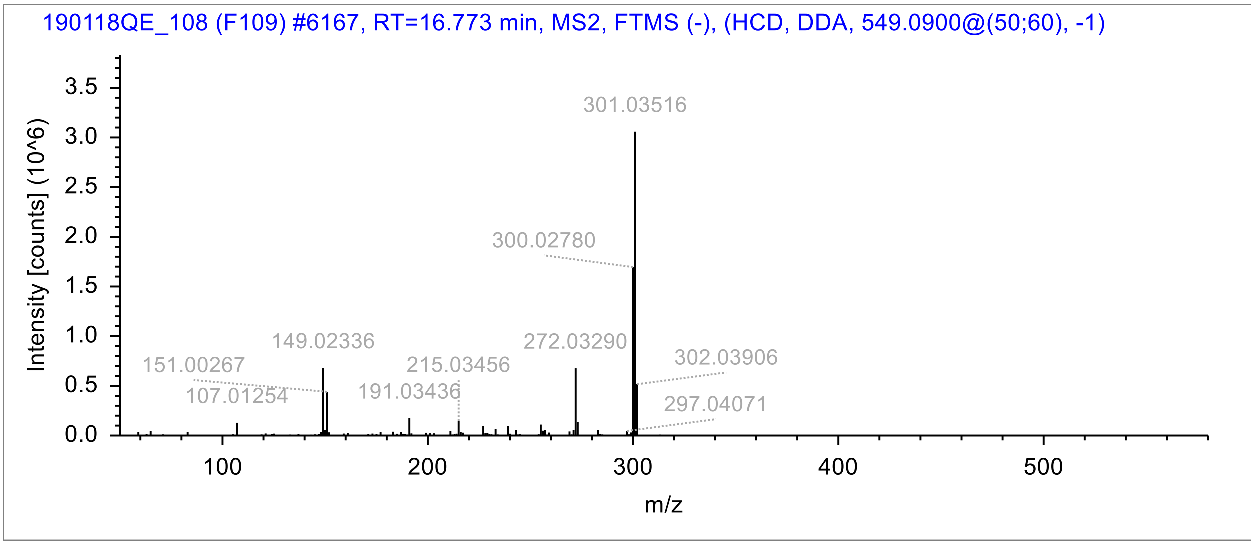

30. Luteolin hexoside	$C_{21}H_{20}O_{11}$	448.1009	16.89	0.84	2/3

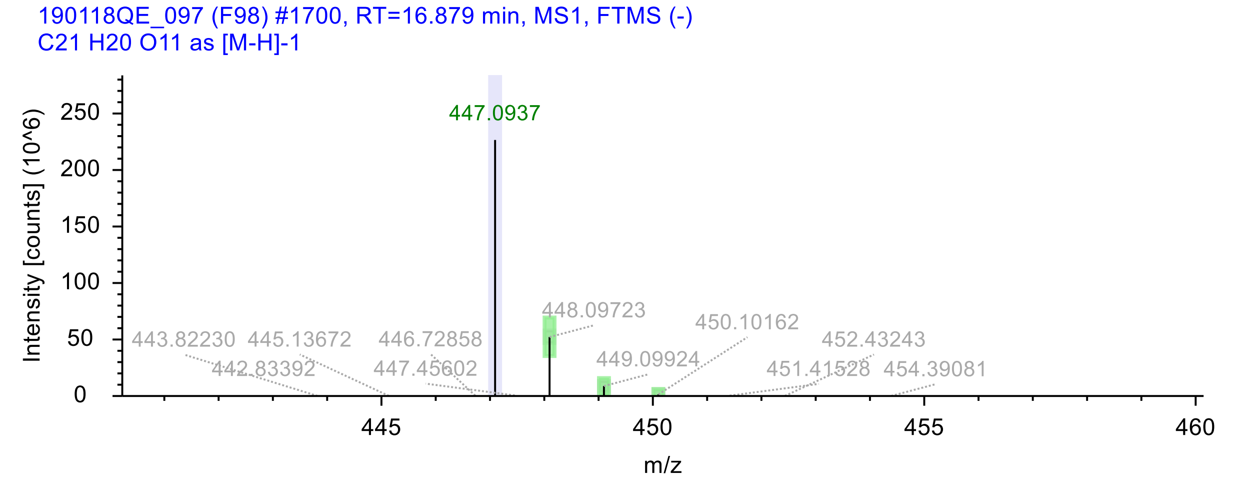

10/20 eV

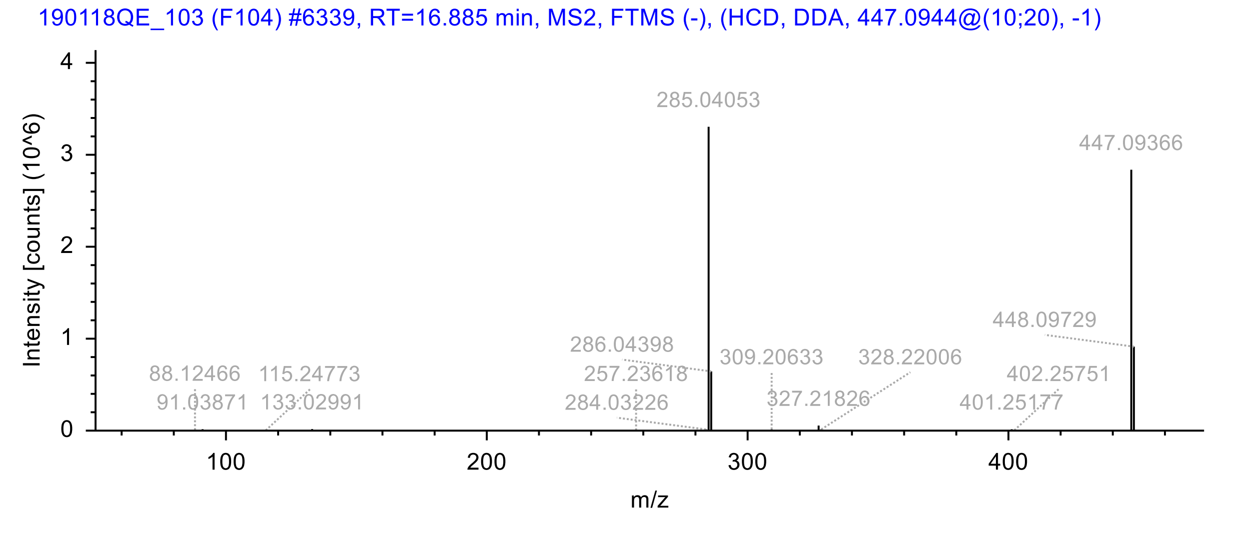

50/60 eV

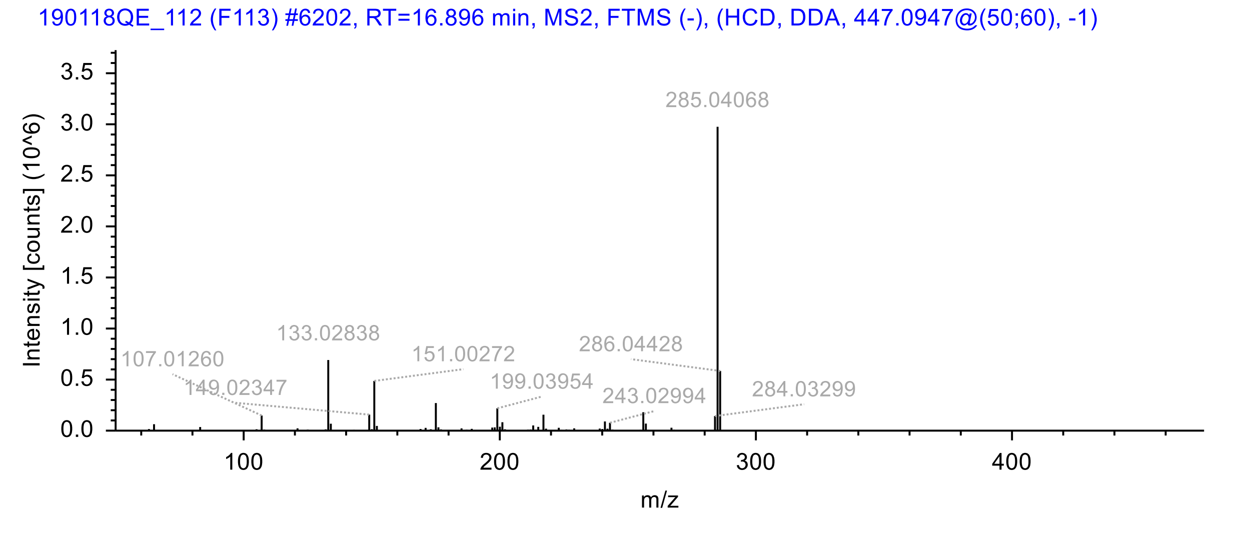

31. Tricetin hexoside	$C_{21}H_{20}O_{12}$	464.0960	17.01	1.18	2/3

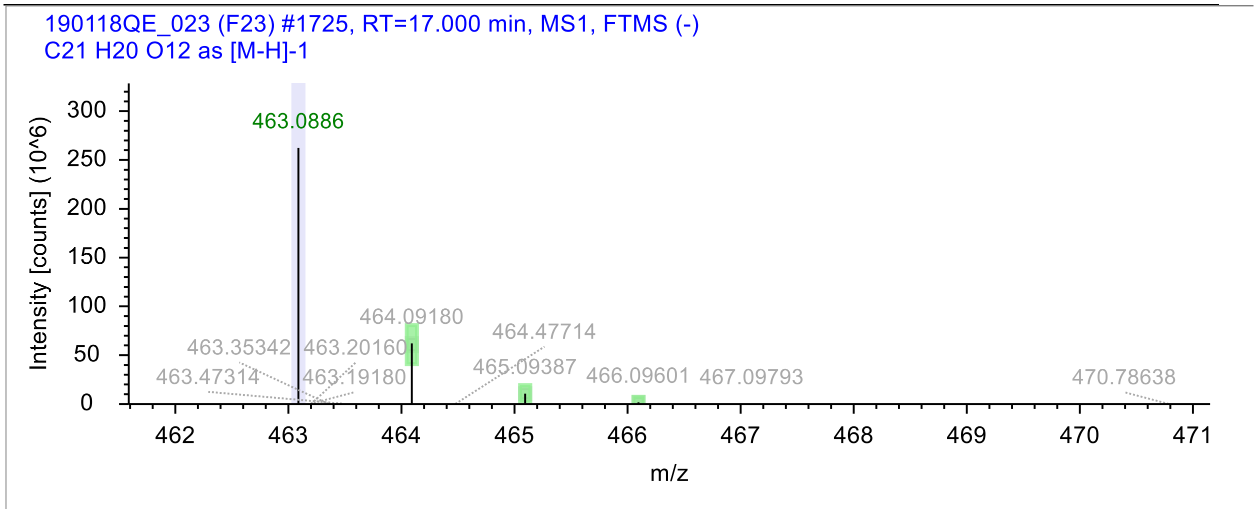

10/20 eV

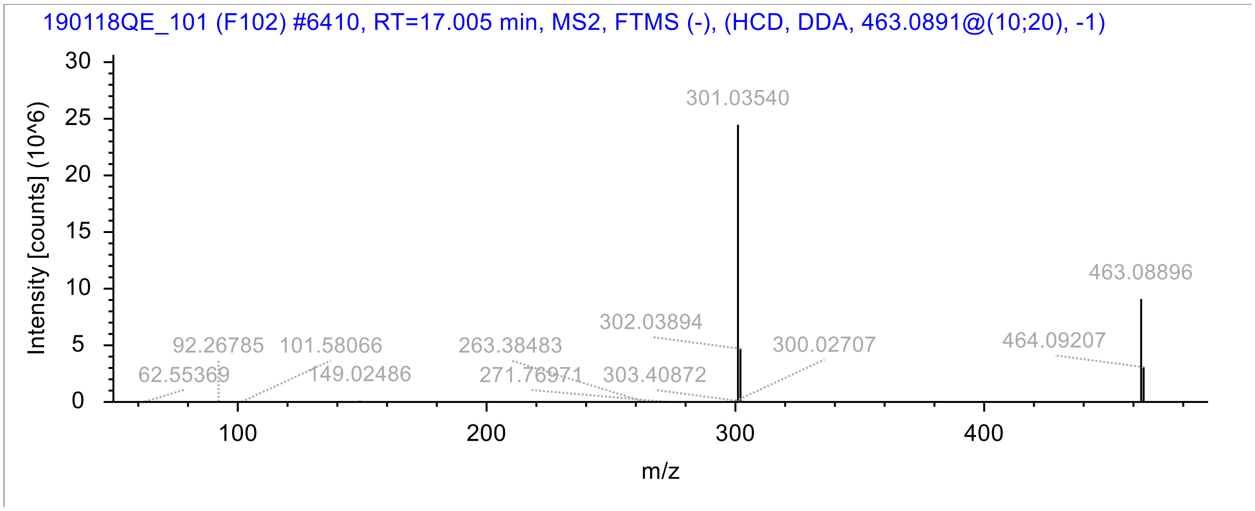

50/60 eV

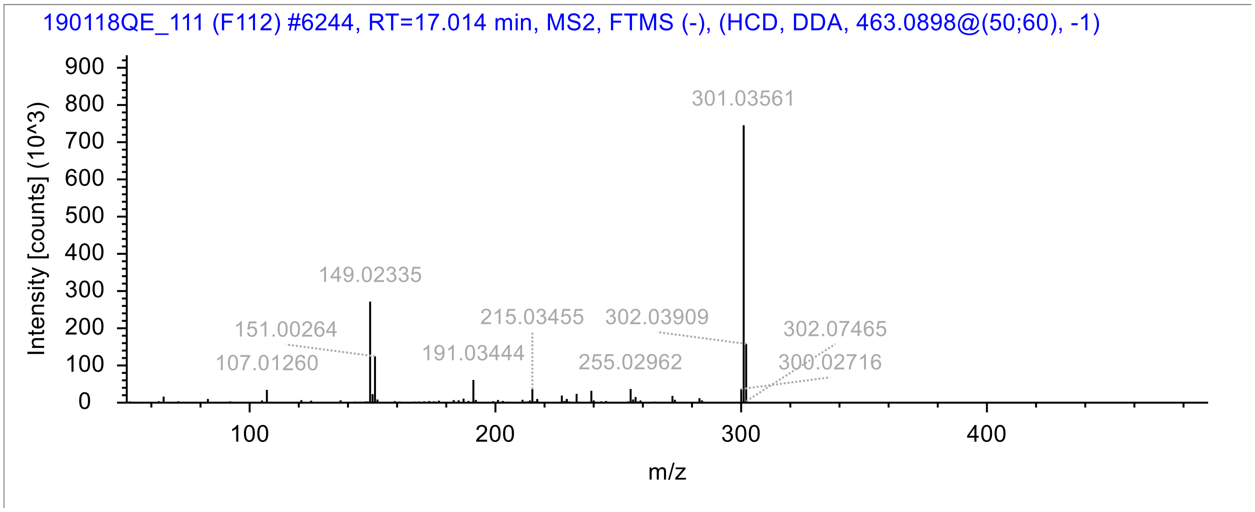

32. Tricetin	$C_{15}H_{10}O_7$	302.0428	17.50	0.43	1

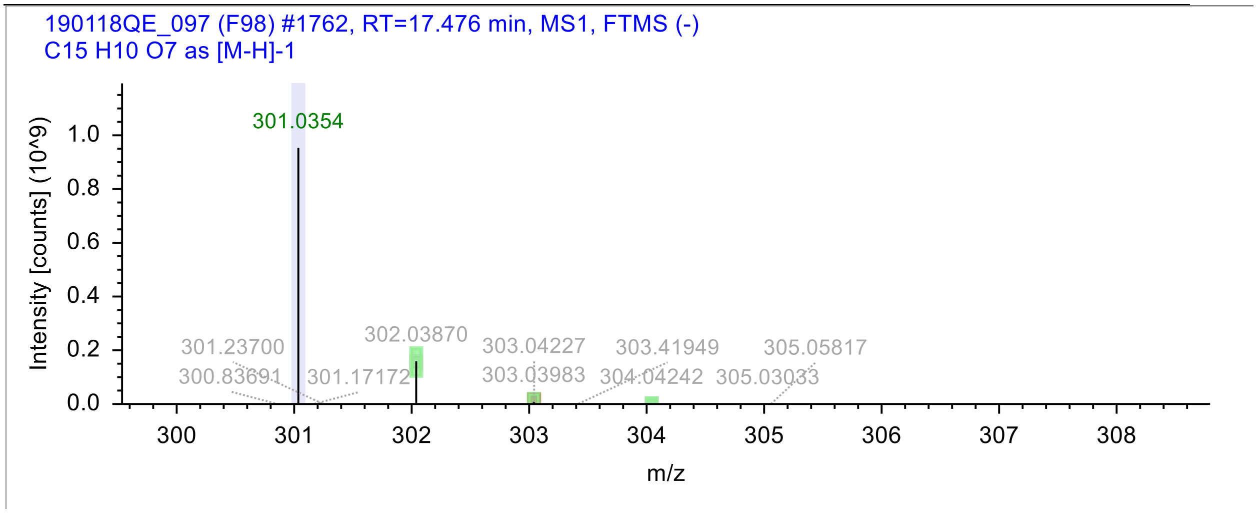

10/20 eV

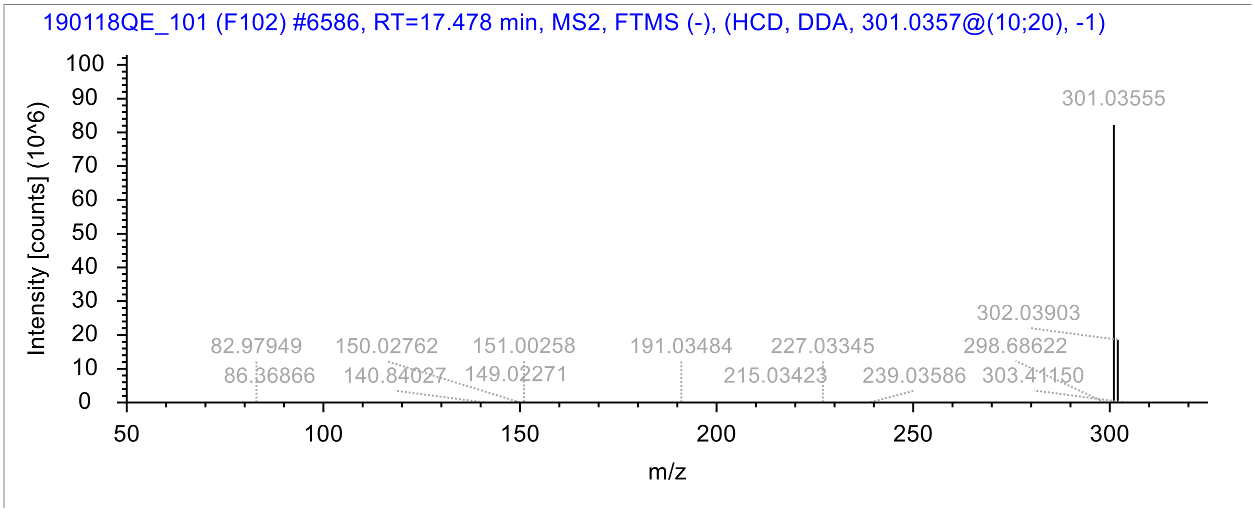

50/60 eV

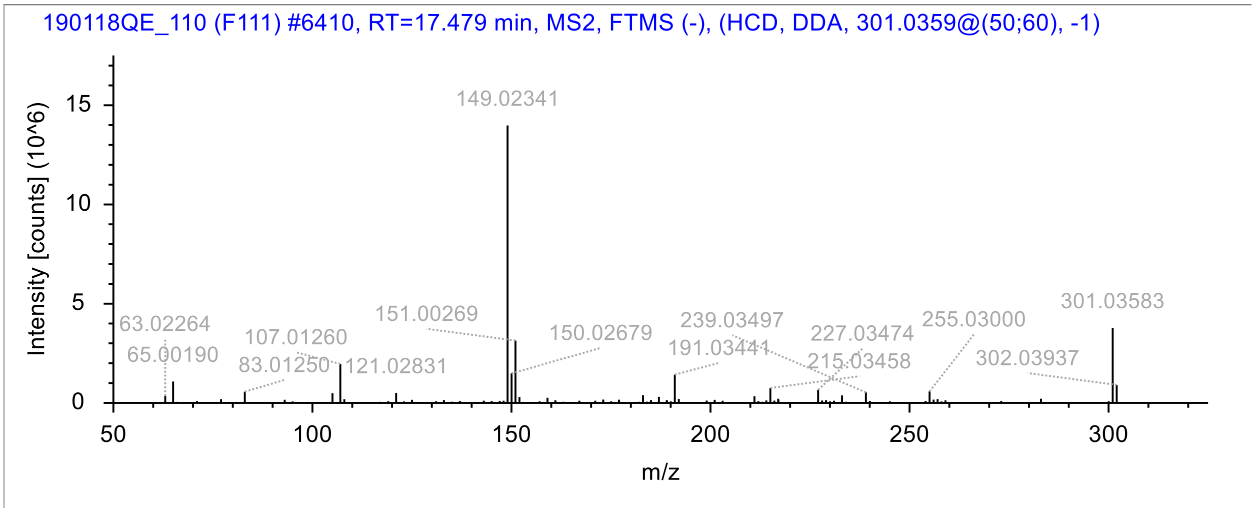

33. Tricetin acetyl hexoside	$C_{23}H_{22}O_{13}$	506.1067	17.86	1.29	2/3

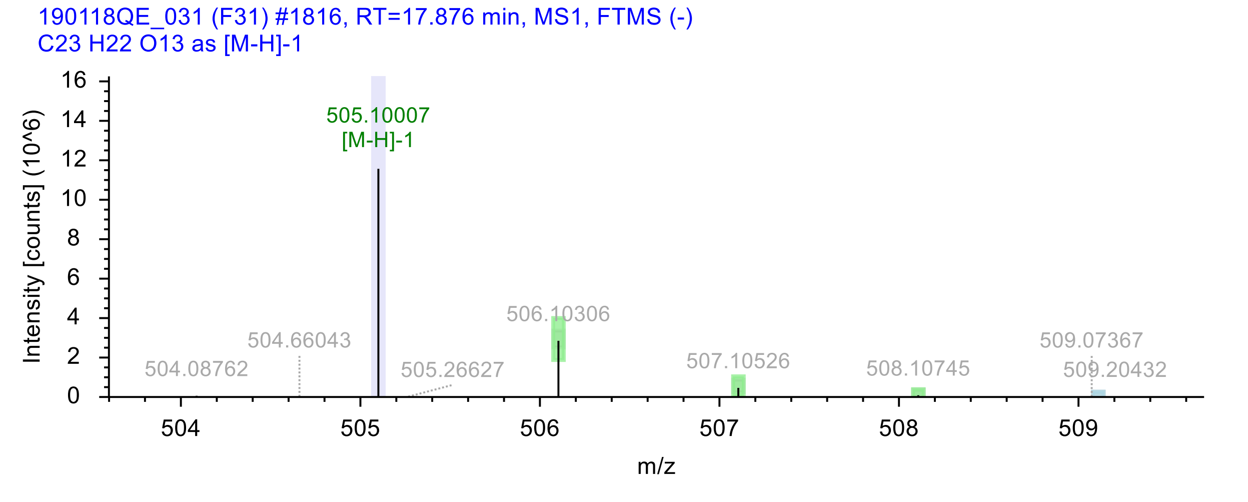

10/20 eV

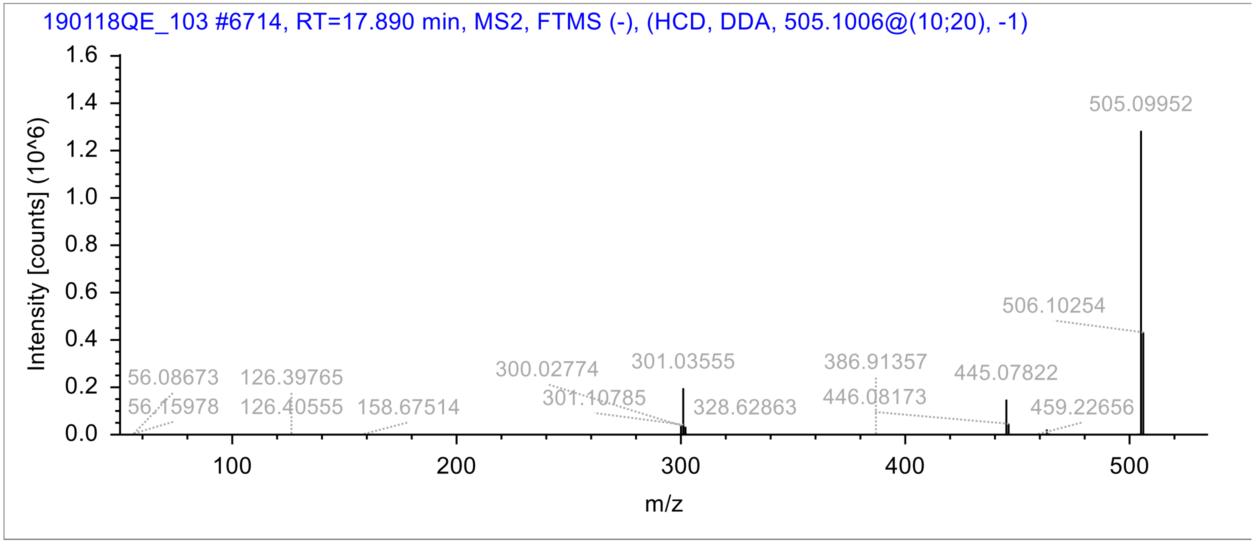

50/60 eV

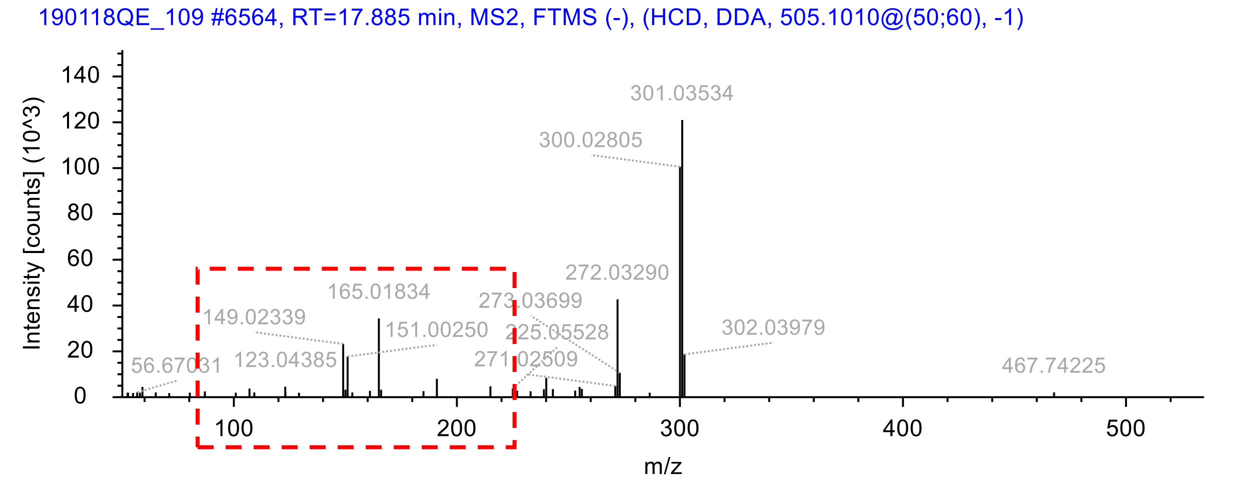

50/60 eV (zoomed-in)

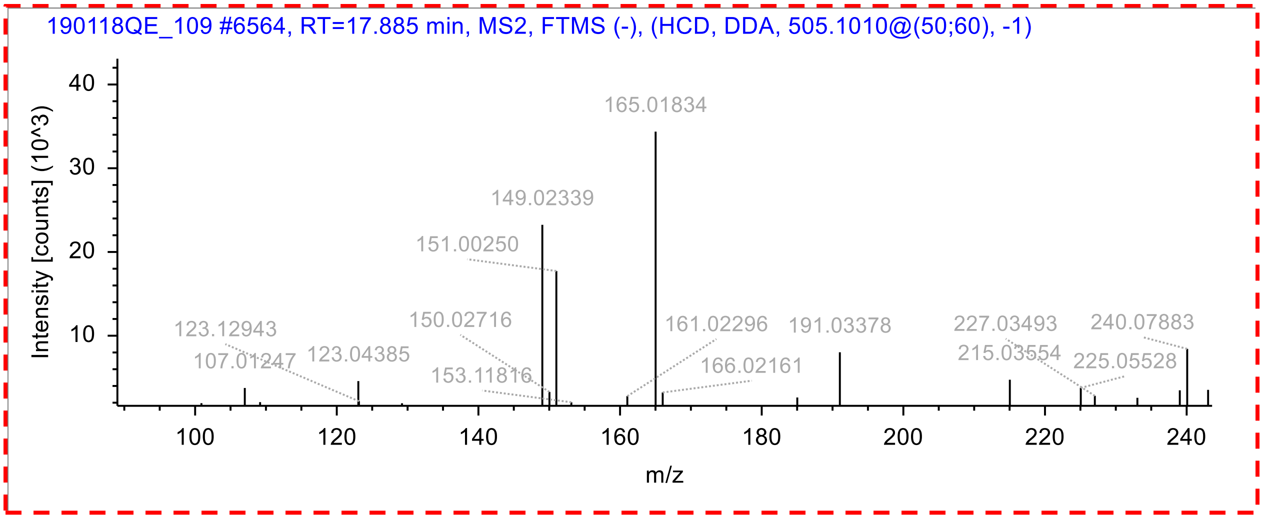

34. Luteolin malonyl hexoside	$C_{24}H_{22}O_{14}$	534.1019	18.13	1.80	2/3

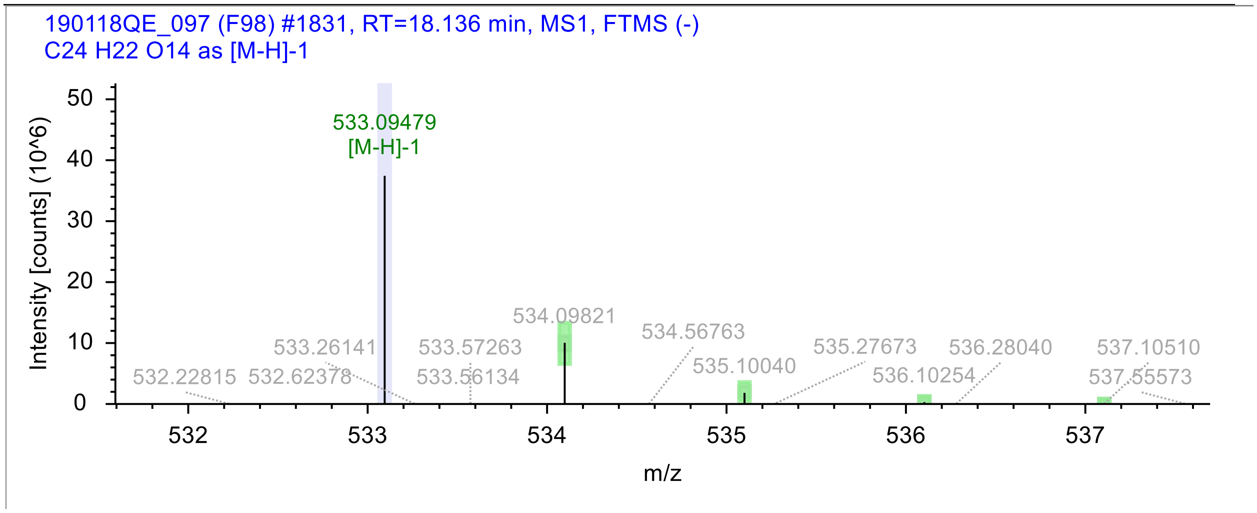

10/20 eV

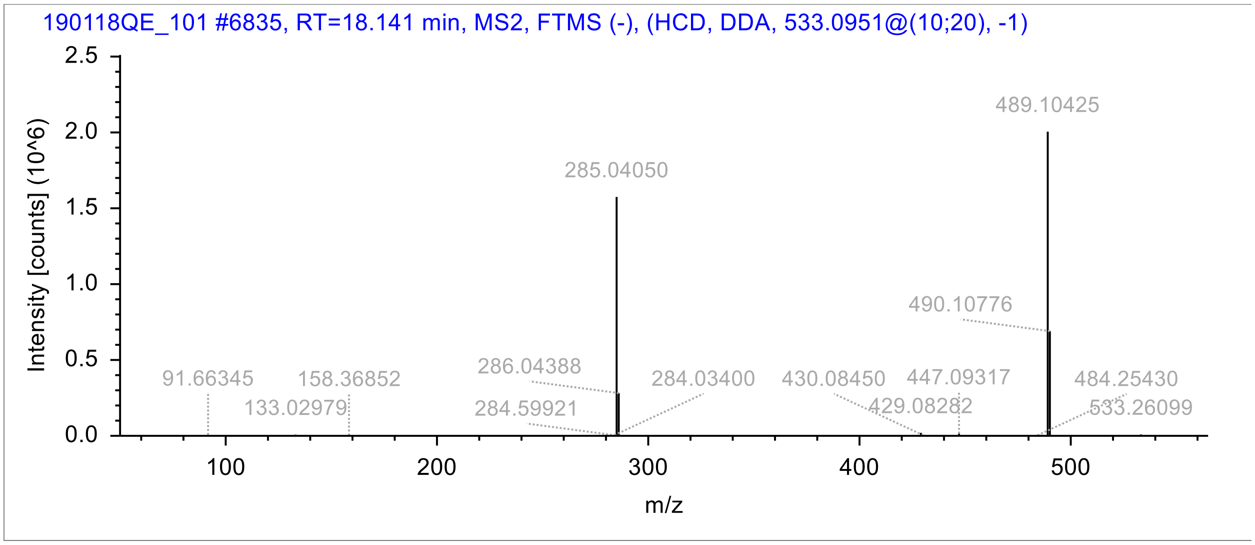

50/60 eV

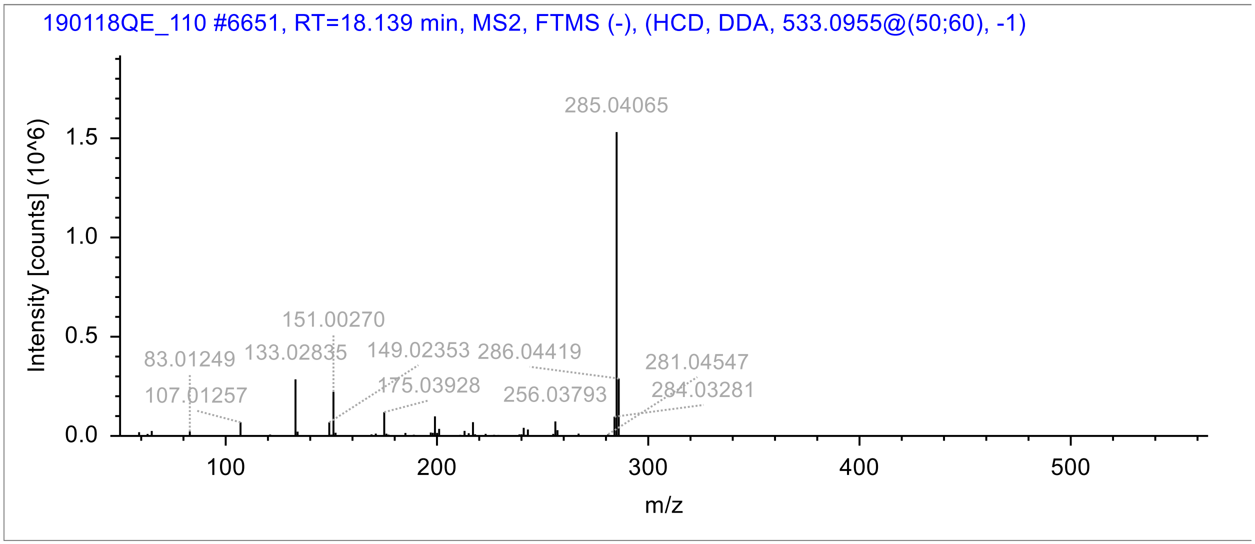

35. Luteolin malonyl hexoside	$C_{24}H_{22}O_{14}$	534.1019	18.58	1.75	2/3

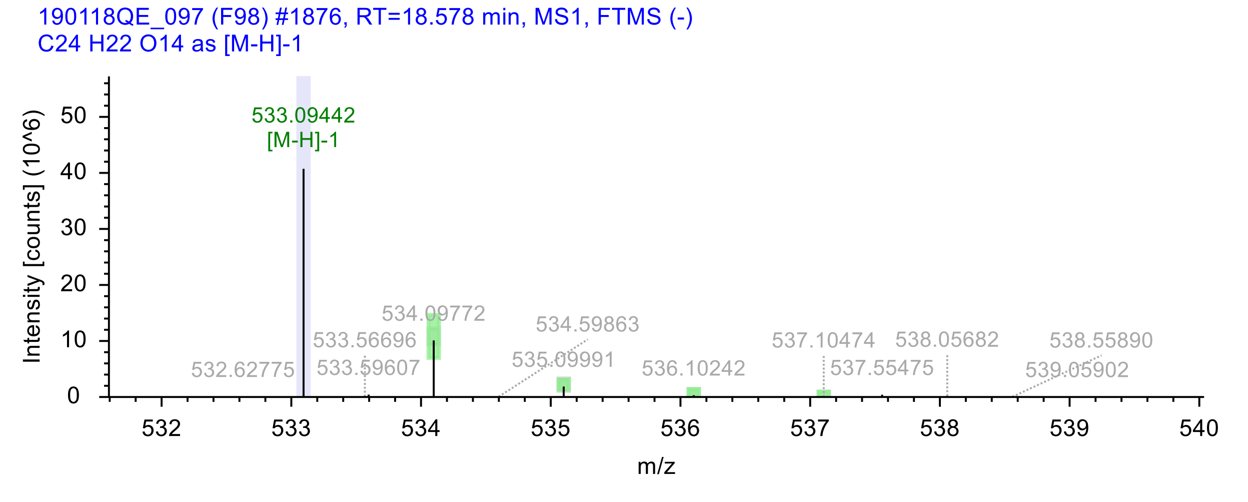

10/20 eV

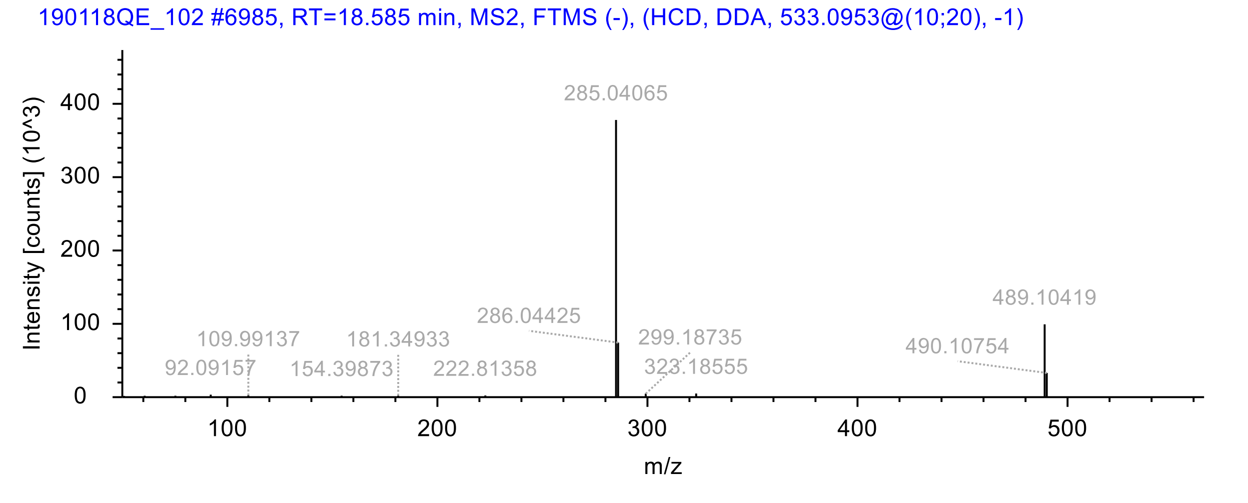

50/60 eV

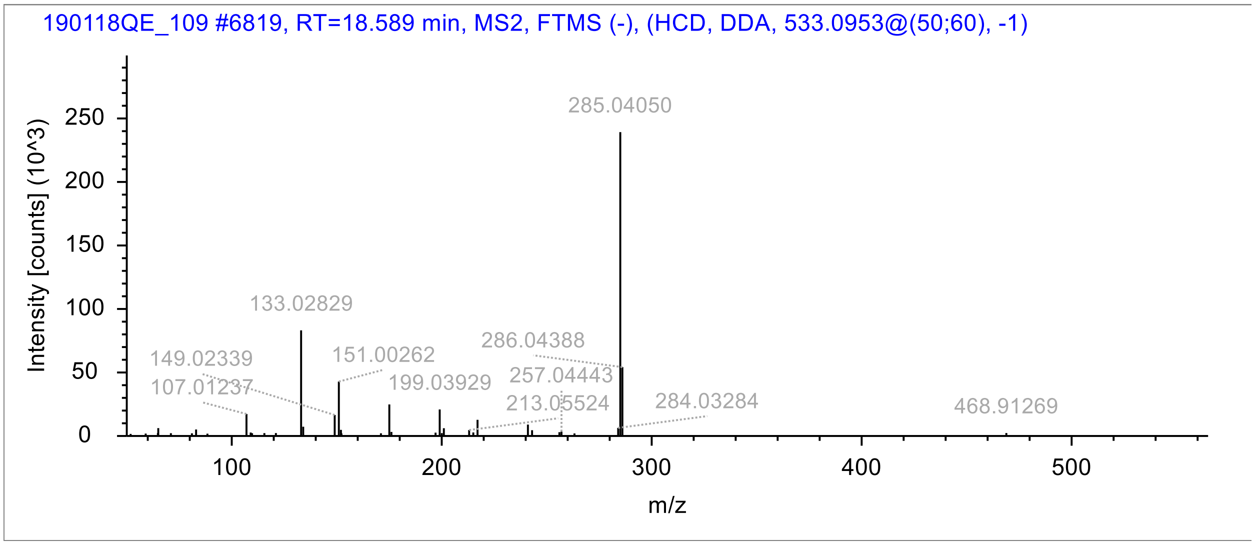

36. Tricetin malonyl hexoside	$C_{24}H_{22}O_{15}$	550.0970	19.07	1.99	2/3

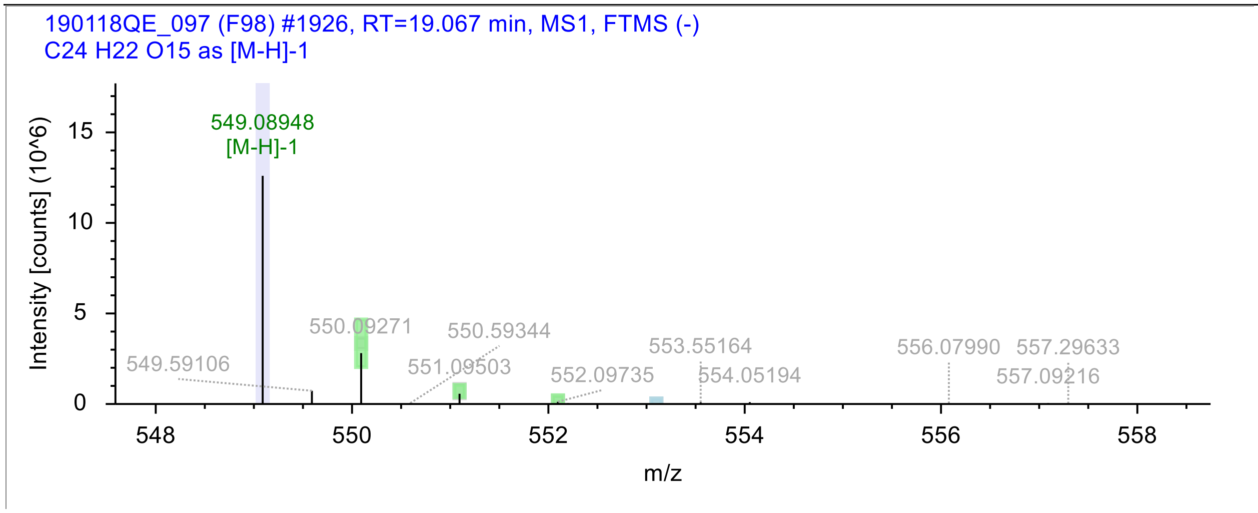

10/20 eV

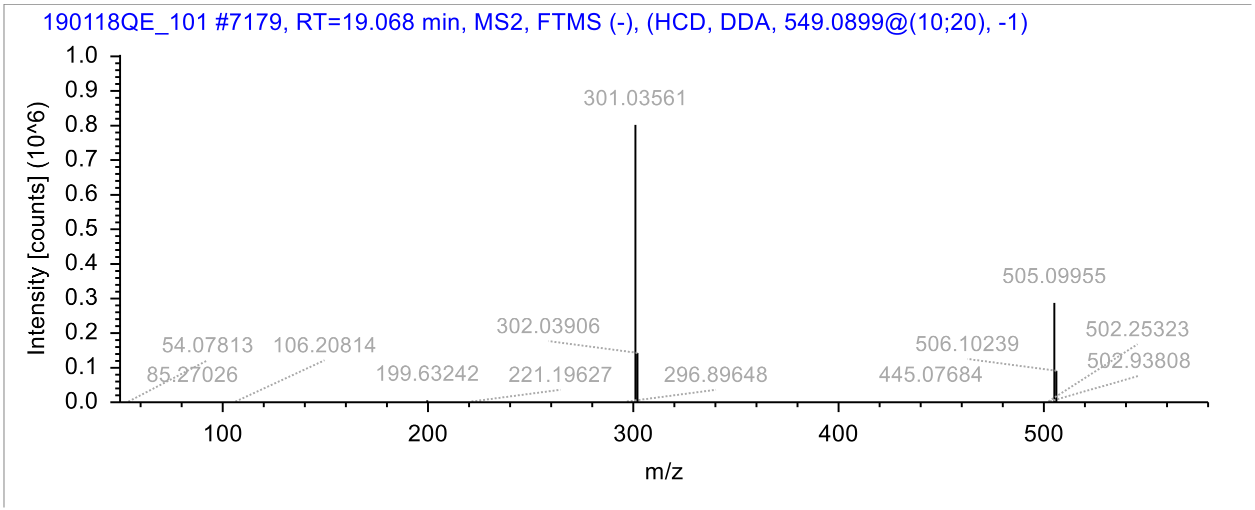

50/60 eV

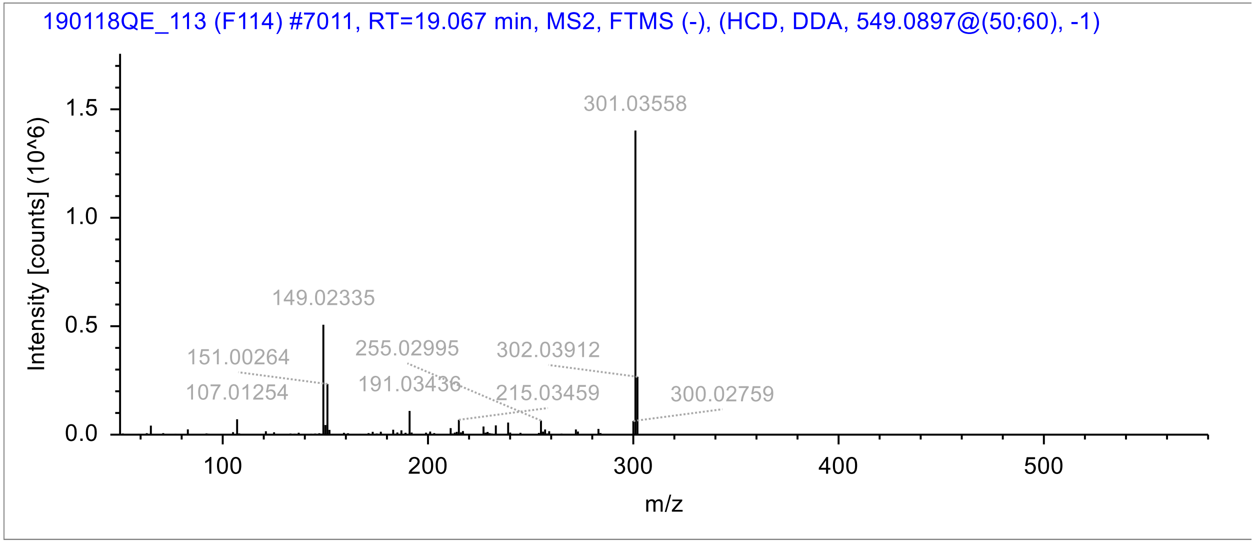

37. Luteolin	$C_{15}H_{10}O_6$	286.0478	19.47	0.33	1

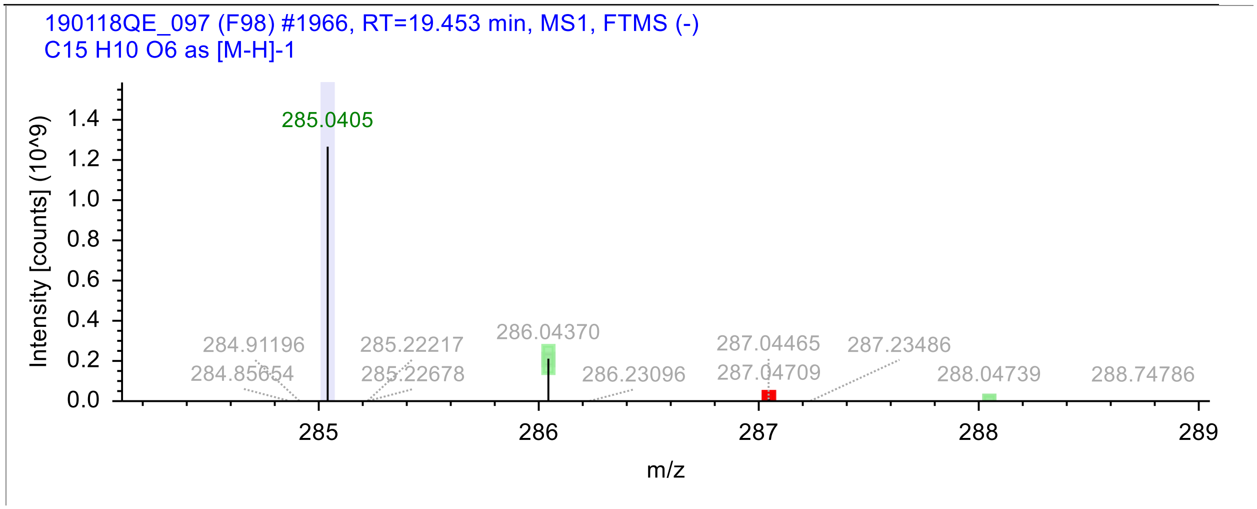

10/20 eV

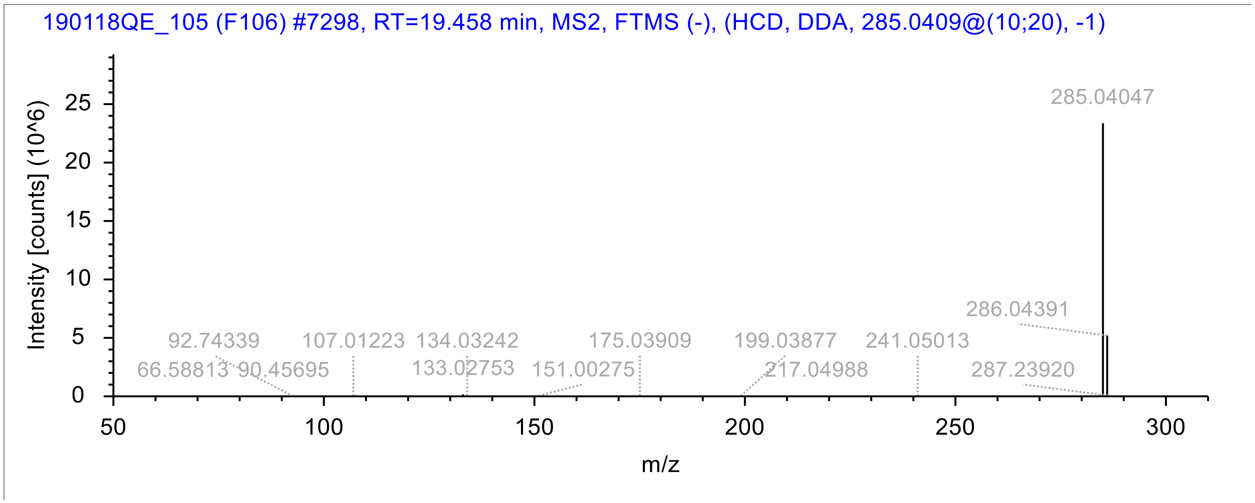

50/60 eV

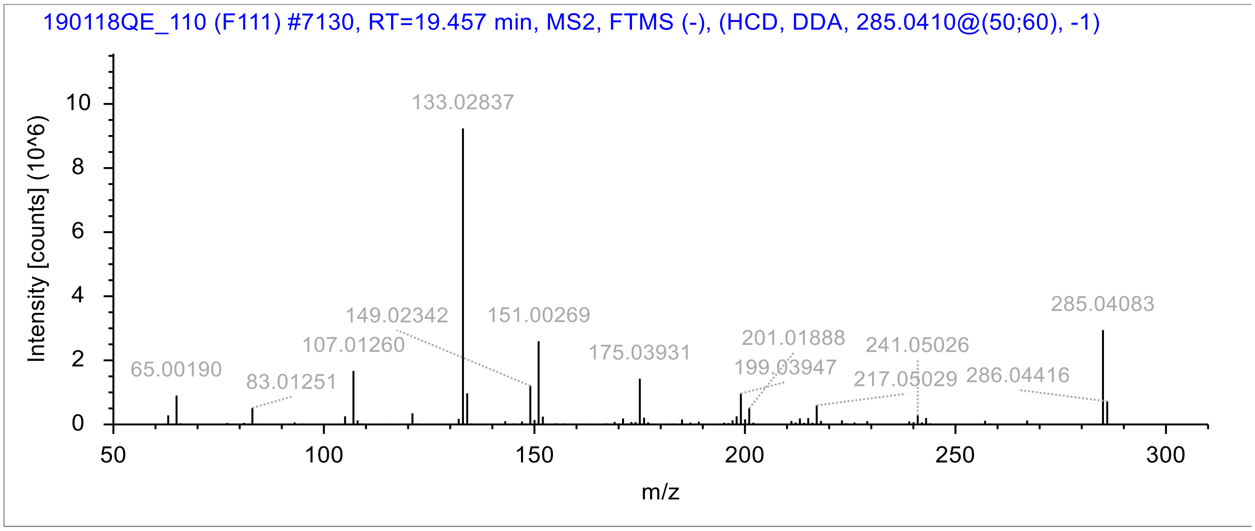

Figure S1. Full scan and MS/MS spectra of the putatively identified metabolites in **Table 2.**

Compounds written in red are upregulated in the volcano plots for green/patterned and black/black seed coats versus green/absent seed coats (23 compounds).

Summary S1. Parameters used in generating the analysis of patterned lentil seed coat samples using the Compound Discoverer software.

Workflow

Search name: PatternedSeedCoats_19Mar2022_CD3.2_NormNone_QC-RSD50_MaxCandidates50_ShiftRefFalse_16DB_MinPeak6M_30-40eV

Search description: Untargeted Metabolomics workflow: Find and identify the differences between samples.

- Performs retention time alignment, unknown compound detection, and compound grouping across all samples. Predicts elemental compositions for all compounds, fills gaps across all samples, and hides chemical background (using Blank samples). Identifies compounds using mzCloud (ddMS2) and ChemSpider (formula or exact mass). Also performs similarity search for all compounds with ddMS2 data using mzCloud. Applies mzLogic algorithm to rank order ChemSpider results. Maps compounds to biological pathways using Metabolika. Applies QC-based batch normalization if QC samples are available. Calculates differential analysis (t-test or ANOVA), determines p-values, adjusted p-values, ratios, fold change, CV, etc.).

Search date: 3/19/2022 9:02:04 PM

Created with Discoverer version: 3.2.0.421

[Input Files (6)]

-->Select Spectra (33)

[Select Spectra (33)]

-->Align Retention Times (26)

[Align Retention Times (26)]

-->Detect Compounds (9)

[Detect Compounds (9)]

-->Group Compounds (31)

[Group Compounds (31)]

-->Fill Gaps (32)

-->Search ChemSpider (23)

-->Map to Metabolika Pathways (34)

-->Search Mass Lists (36)

-->Predict Compositions (29)

-->Assign Compound Annotations (25)

-->Search mzCloud (22)

-->Search mzVault (37)

[Fill Gaps (32)]

-->Apply QC Correction (43)

[Apply QC Correction (43)]

-->Mark Background Compounds (40)

[Search ChemSpider (23)]

-->Apply mzLogic (35)

[Map to Metabolika Pathways (34)]

-->Apply mzLogic (35)

[Search Mass Lists (36)]

-->Apply mzLogic (35)

[Mark Background Compounds (40)]

[Apply mzLogic (35)]

[Predict Compositions (29)]

[Assign Compound Annotations (25)]

[Search mzCloud (22)]

[Search mzVault (37)]

[Differential Analysis (17)]

[Descriptive Statistics (38)]

Processing node 6: Input Files

Input Data:

- File Name(s) (Hidden):

E:\QE Data\2019Jan18\190118QE_001.raw

E:\QE Data\2019Jan18\190118QE_002.raw

E:\QE Data\2019Jan18\190118QE_004.raw

E:\QE Data\2019Jan18\190118QE_005.raw

E:\QE Data\2019Jan18\190118QE_006.raw

E:\QE Data\2019Jan18\190118QE_007.raw

E:\QE Data\2019Jan18\190118QE_008.raw

E:\QE Data\2019Jan18\190118QE_009.raw

E:\QE Data\2019Jan18\190118QE_010.raw

E:\QE Data\2019Jan18\190118QE_011.raw

E:\QE Data\2019Jan18\190118QE_012.raw

E:\QE Data\2019Jan18\190118QE_013.raw

E:\QE Data\2019Jan18\190118QE_014.raw

E:\QE Data\2019Jan18\190118QE_015.raw

E:\QE Data\2019Jan18\190118QE_016.raw

E:\QE Data\2019Jan18\190118QE_017.raw

E:\QE Data\2019Jan18\190118QE_019.raw

E:\QE Data\2019Jan18\190118QE_021.raw

E:\QE Data\2019Jan18\190118QE_022.raw

E:\QE Data\2019Jan18\190118QE_023.raw

E:\QE Data\2019Jan18\190118QE_024.raw

E:\QE Data\2019Jan18\190118QE_026.raw

E:\QE Data\2019Jan18\190118QE_027.raw

E:\QE Data\2019Jan18\190118QE_028.raw

E:\QE Data\2019Jan18\190118QE_029.raw

E:\QE Data\2019Jan18\190118QE_031.raw

E:\QE Data\2019Jan18\190118QE_033.raw

E:\QE Data\2019Jan18\190118QE_035.raw

E:\QE Data\2019Jan18\190118QE_037.raw

E:\QE Data\2019Jan18\190118QE_038.raw

E:\QE Data\2019Jan18\190118QE_039.raw

E:\QE Data\2019Jan18\190118QE_040.raw

E:\QE Data\2019Jan18\190118QE_041.raw

E:\QE Data\2019Jan18\190118QE_042.raw

E:\QE Data\2019Jan18\190118QE_043.raw

E:\QE Data\2019Jan18\190118QE_044.raw

E:\QE Data\2019Jan18\190118QE_045.raw

E:\QE Data\2019Jan18\190118QE_047.raw

E:\QE Data\2019Jan18\190118QE_049.raw

E:\QE Data\2019Jan18\190118QE_050.raw

E:\QE Data\2019Jan18\190118QE_051.raw

E:\QE Data\2019Jan18\190118QE_053.raw

E:\QE Data\2019Jan18\190118QE_054.raw

E:\QE Data\2019Jan18\190118QE_055.raw

E:\QE Data\2019Jan18\190118QE_056.raw

E:\QE Data\2019Jan18\190118QE_057.raw

E:\QE Data\2019Jan18\190118QE_058.raw

E:\QE Data\2019Jan18\190118QE_060.raw

E:\QE Data\2019Jan18\190118QE_061.raw

E:\QE Data\2019Jan18\190118QE_063.raw

E:\QE Data\2019Jan18\190118QE_065.raw

E:\QE Data\2019Jan18\190118QE_066.raw

E:\QE Data\2019Jan18\190118QE_067.raw

E:\QE Data\2019Jan18\190118QE_068.raw

E:\QE Data\2019Jan18\190118QE_069.raw

E:\QE Data\2019Jan18\190118QE_070.raw

E:\QE Data\2019Jan18\190118QE_071.raw

E:\QE Data\2019Jan18\190118QE_072.raw

E:\QE Data\2019Jan18\190118QE_074.raw

E:\QE Data\2019Jan18\190118QE_076.raw

E:\QE Data\2019Jan18\190118QE_077.raw

E:\QE Data\2019Jan18\190118QE_078.raw

E:\QE Data\2019Jan18\190118QE_079.raw

E:\QE Data\2019Jan18\190118QE_081.raw

E:\QE Data\2019Jan18\190118QE_082.raw

E:\QE Data\2019Jan18\190118QE_083.raw

E:\QE Data\2019Jan18\190118QE_084.raw

E:\QE Data\2019Jan18\190118QE_085.raw

E:\QE Data\2019Jan18\190118QE_086.raw

E:\QE Data\2019Jan18\190118QE_090.raw

E:\QE Data\2019Jan18\190118QE_092.raw

E:\QE Data\2019Jan18\190118QE_094.raw

E:\QE Data\2019Jan18\190118QE_095.raw

E:\QE Data\2019Jan18\190118QE_096.raw

E:\QE Data\2019Jan18\190118QE_097.raw

E:\QE Data\2019Jan18\190118QE_098.raw

E:\QE Data\2019Jan18\190118QE_100.raw

Processing node 33: Select Spectra

----

1. Spectrum Properties Filter:

- Lower RT Limit: 1.2

- Upper RT Limit: 25
- First Scan: 0
- Last Scan: 0
- Ignore Specified Scans: (not specified)
- Lowest Charge State: 0
- Highest Charge State: 3
- Min. Precursor Mass: 140 Da
- Max. Precursor Mass: 4000 Da
- Total Intensity Threshold: 0
- Minimum Peak Count: 1

2. Scan Event Filters:
- Mass Analyzer: (not specified)
- MS Order: Any
- Activation Type: (not specified)
- Min. Collision Energy: 0
- Max. Collision Energy: 1000
- Scan Type: Any
- Polarity Mode: Any

3. Peak Filters:
- S/N Threshold (FT-only): 1.5

4. Replacements for Unrecognized Properties:
- Unrecognized Charge Replacements: 1
- Unrecognized Mass Analyzer Replacements: ITMS
- Unrecognized MS Order Replacements: MS2
- Unrecognized Activation Type Replacements: CID
- Unrecognized Polarity Replacements: +
- Unrecognized MS Resolution@200 Replacements: 60000
- Unrecognized MSn Resolution@200 Replacements: 30000

5. General Settings:

- Precursor Selection: Use MS1 Precursor
- Use Isotope Pattern in Precursor Reevaluation: True
- Provide Profile Spectra: Automatic
- Store Chromatograms: False

Processing node 26: Align Retention Times

----

1. General Settings:

- Alignment Model: Adaptive curve
- Alignment Fallback: Use Linear Model
- Maximum Shift [min]: 2
- Shift Reference File: False
- Mass Tolerance: 5 ppm
- Remove Outlier: True

Processing node 9: Detect Compounds

----

1. General Settings:

- Mass Tolerance [ppm]: 5 ppm
- Intensity Tolerance [%]: 30
- S/N Threshold: 3
- Min. Peak Intensity: 6000000
- Ions:

    [2M+FA-H]-1

    [2M-H]-1

    [M+FA-H]-1

    [M-2H]-2

    [M-H]-1

    [M-H-H2O]-1

- Base Ions: [M-H]-1
- Min. Element Counts: C H O
- Max. Element Counts: C150 H300 N10 Na2 O150 P S2

2. Peak Detection:
- Filter Peaks: True
- Max. Peak Width [min]: 0.5
- Remove Singlets: True
- Min. # Scans per Peak: 10
- Min. # Isotopes: 1

3. Isotope Grouping:
- Min. Spectral Distance Score: 0
- Remove Potentially False Positive Isotopes: True

Processing node 31: Group Compounds

----

1. Compound Consolidation:
- Mass Tolerance: 5 ppm
- RT Tolerance [min]: 0.2

2. Fragment Data Selection:
- Preferred Ions: [M-2H]-2; [M-H]-1

Processing node 32: Fill Gaps

----

1. General Settings:
- Mass Tolerance: 5 ppm

- S/N Threshold: 1.5

- Use Real Peak Detection: True

Processing node 43: Apply QC Correction

----

1. General Settings:

- Regression Model: Linear

- Min. QC Coverage [%]: 75

- Max. QC Area RSD [%]: 30

- Max. Corrected QC Area RSD [%]: 25

- Max. # Files Between QC Files: 10

Processing node 40: Mark Background Compounds

----

1. General Settings:

- Max. Sample/Blank: 5

- Max. Blank/Sample: 0

- Hide Background: True

Processing node 23: Search ChemSpider

----

1. Search Settings:

- Database(s):

    BioCyc

    Carotenoids Database

    Cayman Chemical

    ChEBI

    ChEMBL

    FooDB

    Human Metabolome Database

    KEGG

MassBank

NIST

Phenol-Explorer

PlantCyc

Royal Society of Chemistry

Sigma-Aldrich

SMPDB Small Molecule Pathway Database

The Merck Index Online

- Search Mode: By Formula or Mass
- Mass Tolerance: 5 ppm
- Max. # of results per compound: 100
- Max. # of Predicted Compositions to be searched per Compound: 3
- Result Order (for Max. # of results per compound): Order By Reference Count (DESC)

2. Predicted Composition Annotation:

- Check All Predicted Compositions: False

Processing node 35: Apply mzLogic

----

1. Search Settings:

- FT Fragment Mass Tolerance: 10 ppm
- IT Fragment Mass Tolerance: 0.4 Da
- Max. # Compounds: 10
- Max. # mzCloud Similarity Results to consider per Compound: 10
- Match Factor Threshold: 30

Processing node 34: Map to Metabolika Pathways

----

1. Search Settings:

- Metabolika Pathways: (3R)-linalool biosynthesis.metabolika|2-nitrobenzoate degradation I.metabolika|2-oxobutanoate degradation I.metabolika|3-phenylpropanoate and 3-(3-hydroxyphenyl)propanoate degradation.metabolika|3-phenylpropanoate degradation.metabolika|Acetyl-

CoA fermentation to butanoate II.metabolika|Adenosylcobalamin biosynthesis I (anaerobic).metabolika|Adenosylcobalamin biosynthesis II (aerobic).metabolika|Allantoin degradation IV (anaerobic).metabolika|Allantoin degradation to glyoxylate I.metabolika|Allantoin degradation to glyoxylate II.metabolika|Allantoin degradation to glyoxylate III.metabolika|Ammonia assimilation cycle I.metabolika|Ammonia assimilation cycle III.metabolika|Ammonia oxidation IV (autotrophic ammonia oxidizers).metabolika|Anaerobic aromatic compound degradation (Thauera aromatica).metabolika|Anaerobic energy metabolism (invertebrates, mitochondrial).metabolika|Arachidonate biosynthesis III (6-desaturase, mammals).metabolika|Archaetidylinositol biosynthesis.metabolika|Archaetidylserine and archaetidylethanolamine biosynthesis.metabolika|Arginine, ornithine and proline interconversion.metabolika|Aromatic compounds degradation via ss-ketoadipate.metabolika|Aspartate superpathway.metabolika|B-carotene biosynthesis (engineered).metabolika|Bacillibactin biosynthesis.metabolika|Benzoate biosynthesis I (CoA-dependent, ss-oxidative).metabolika|Benzoate biosynthesis III (CoA-dependent, non-ss-oxidative).metabolika|Benzoate fermentation (to acetate and cyclohexane carboxylate).metabolika|Biotin biosynthesis I.metabolika|Biotin biosynthesis II.metabolika|Bitter acids biosynthesis.metabolika|Caffeine degradation IV (bacteria, via demethylation and oxidation).metabolika|Cardiolipin and phosphatidylethanolamine biosynthesis (Xanthomonas).metabolika|Catechol degradation I (meta-cleavage pathway).metabolika|Catechol degradation II (meta-cleavage pathway).metabolika|Catechol degradation III (ortho-cleavage pathway).metabolika|Cellulose and hemicellulose degradation (cellulolosome).metabolika|Chitin biosynthesis.metabolika|Cholesterol biosynthesis I.metabolika|Cholesterol biosynthesis II (via 24,25-dihydrolanosterol).metabolika|Cholesterol biosynthesis III (via desmosterol).metabolika|Choline degradation IV.metabolika|Choline-O-sulfate degradation.metabolika|Chondroitin sulfate biosynthesis.metabolika|Chorismate biosynthesis I.metabolika|Chorismate biosynthesis II (archaea).metabolika|Colanic acid building blocks biosynthesis.metabolika|Crotonate fermentation (to acetate and cyclohexane carboxylate).metabolika|Curcuminoid biosynthesis.metabolika|D-serine metabolism.metabolika|Dermatan sulfate biosynthesis.metabolika|Enterobacterial common antigen biosynthesis.metabolika|Enterobactin biosynthesis.metabolika|G-butyrobetaine degradation.metabolika|GABA shunt.metabolika|Gamma-glutamyl cycle.metabolika|Gluconeogenesis II (Methanobacterium thermoautotrophicum).metabolika|Glycerol and glycerophosphodiester degradation.metabolika|Glycerol degradation to butanol.metabolika|Glycine biosynthesis II.metabolika|Heparan sulfate biosynthesis.metabolika|Hexitol fermentation to lactate, formate, ethanol and acetate.metabolika|Homolactic fermentation.metabolika|Hyperxanthone E biosynthesis.metabolika|Icosapentaenoate biosynthesis III (fungi).metabolika|Icosapentaenoate biosynthesis IV (bacteria).metabolika|Isoprene biosynthesis I.metabolika|Kanamycin biosynthesis.metabolika|Kauralexin biosynthesis.metabolika|Kdo transfer to lipid IVA III (Chlamydia).metabolika|Ketogluconate metabolism.metabolika|L-alanine fermentation to propanoate and acetate.metabolika|L-arginine biosynthesis I (via L-ornithine).metabolika|L-arginine degradation V (arginine deiminase pathway).metabolika|L-ascorbate biosynthesis V.metabolika|L-cysteine biosynthesis IV (from L-methionine).metabolika|L-cysteine biosynthesis IV (fungi).metabolika|L-glutamate and L-glutamine biosynthesis.metabolika|L-glutamate degradation IX (via 4-aminobutanoate).metabolika|L-glutamate degradation VII (to butanoate).metabolika|L-glutamate degradation VIII (to propanoate).metabolika|L-homoserine and L-methionine biosynthesis.metabolika|L-methionine biosynthesis III.metabolika|L-methionine salvage cycle I (bacteria and plants).metabolika|L-methionine salvage cycle II (plants).metabolika|L-methionine salvage cycle III.metabolika|L-tryptophan degradation III (eukaryotic).metabolika|L-tryptophan degradation IX.metabolika|L-tryptophan degradation XI (mammalian, via kynurenine).metabolika|L-tryptophan degradation XII (Geobacillus).metabolika|L-tyrosine degradation IV (to 4-methylphenol).metabolika|Mandelate degradation to acetyl-CoA.metabolika|Meta cleavage pathway of aromatic compounds.metabolika|Methanobacterium thermoautotrophicum biosynthetic metabolism.metabolika|Methanol and methylamine oxidation to formaldehyde.metabolika|Methanol oxidation to carbon dioxide.metabolika|Methylglyoxal degradation

IV.metabolika|MRNA capping II.metabolika|Myo-, chiro- and scillo-inositol degradation.metabolika|N-acetylglucosamine degradation II.metabolika|NAD biosynthesis II (from tryptophan).metabolika|NAD salvage pathway III.metabolika|Naphthalene degradation to acetyl-CoA.metabolika|Nitrifier denitrification.metabolika|Novobiocin biosynthesis.metabolika|O-antigen building blocks biosynthesis (E. coli).metabolika|Oxygenic photosynthesis.metabolika|P-cumate degradation.metabolika|P-cymene degradation.metabolika|Pentose phosphate pathway.metabolika|Peptidoglycan biosynthesis I (meso-diaminopimelate containing).metabolika|Peptidoglycan biosynthesis II (staphylococci).metabolika|Peptidoglycan biosynthesis III (mycobacteria).metabolika|Peptidoglycan biosynthesis IV (Enterococcus faecium).metabolika|Peptidoglycan biosynthesis V (ss-lactam resistance).metabolika|Phosphatidylglycerol biosynthesis I (plastidic).metabolika|Phosphatidylglycerol biosynthesis II (non-plastidic).metabolika|Plant sterol biosynthesis.metabolika|Polyisoprenoid biosynthesis (E. coli).metabolika|Purine nucleotides degradation I (plants).metabolika|Purine nucleotides degradation II (aerobic).metabolika|Pyrimidine nucleobases salvage II.metabolika|Pyruvate fermentation to acetate and alanine.metabolika|Pyruvate fermentation to acetate and lactate I.metabolika|Pyruvate fermentation to acetate and lactate II.metabolika|Pyruvate fermentation to acetate I.metabolika|Pyruvate fermentation to acetate III.metabolika|Pyruvate fermentation to acetate IV.metabolika|Pyruvate fermentation to acetate V.metabolika|Pyruvate fermentation to acetate VI.metabolika|Pyruvate fermentation to acetate VII.metabolika|Reactive oxygen species degradation.metabolika|S-adenosyl-L-methionine cycle I.metabolika|Salicylate glucosides biosynthesis I.metabolika|Sphingolipid biosynthesis (mammals).metabolika|Sucrose biosynthesis I (from photosynthesis).metabolika|Sulfate reduction I (assimilatory).metabolika|Superpathway avenacin A biosynthesis.metabolika|Superpathway NADNADP - NADHNADPH interconversion (yeast).metabolika|Superpathway of (Kdo)2-lipid A biosynthesis.metabolika|Superpathway of (R,R)-butanediol biosynthesis.metabolika|Superpathway of 1D-myo-inositol hexakisphosphate biosynthesis (plants).metabolika|Superpathway of 2,3-butanediol biosynthesis.metabolika|Superpathway of 4-aminobutanoate degradation.metabolika|Superpathway of 4-hydroxybenzoate biosynthesis (yeast).metabolika|Superpathway of 5-aminoimidazole ribonucleotide biosynthesis.metabolika|Superpathway of acetate utilization and formation.metabolika|Superpathway of acetyl-CoA biosynthesis.metabolika|Superpathway of acrylonitrile degradation.metabolika|Superpathway of adenosine nucleotides de novo biosynthesis I.metabolika|Superpathway of adenosine nucleotides de novo biosynthesis II.metabolika|Superpathway of aerobic toluene degradation.metabolika|Superpathway of aflatoxin biosynthesis.metabolika|Superpathway of allantoin degradation in plants.metabolika|Superpathway of allantoin degradation in yeast.metabolika|Superpathway of Allium flavor precursors.metabolika|Superpathway of ammonia assimilation (plants).metabolika|Superpathway of anaerobic energy metabolism (invertebrates).metabolika|Superpathway of anaerobic sucrose degradation.metabolika|Superpathway of anthocyanin biosynthesis (from cyanidin and cyanidin 3-O-glucoside).metabolika|Superpathway of anthocyanin biosynthesis (from delphinidin 3-O-glucoside).metabolika|Superpathway of anthocyanin biosynthesis (from pelargonidin 3-O-glucoside).metabolika|Superpathway of arginine and polyamine biosynthesis.metabolika|Superpathway of aromatic amino acid biosynthesis.metabolika|Superpathway of aromatic compound degradation via 2-oxopent-4-enoate.metabolika|Superpathway of aromatic compound degradation via 3-oxoadipate.metabolika|Superpathway of atrazine degradation.metabolika|Superpathway of bacteriochlorophyll a biosynthesis.metabolika|Superpathway of benzoxazinoid glucosides biosynthesis.metabolika|Superpathway of betalain biosynthesis.metabolika|Superpathway of branched chain amino acid biosynthesis.metabolika|Superpathway of butirocin biosynthesis.metabolika|Superpathway of C1 compounds oxidation to CO2.metabolika|Superpathway of C28 brassinosteroid biosynthesis.metabolika|Superpathway of candicidin biosynthesis.metabolika|Superpathway of carotenoid biosynthesis.metabolika|Superpathway of CDP-glucose-derived O-antigen building blocks biosynthesis.metabolika|Superpathway of cholesterol biosynthesis.metabolika|Superpathway of cholesterol degradation I (cholesterol oxidase).metabolika|Superpathway of cholesterol degradation II (cholesterol dehydrogenase).metabolika|Superpathway of choline biosynthesis.metabolika|Superpathway of

chorismate metabolism.metabolika|Superpathway of CMP-sialic acids biosynthesis.metabolika|Superpathway of coenzyme A biosynthesis I.metabolika|Superpathway of coenzyme A biosynthesis II (plants).metabolika|Superpathway of coenzyme A biosynthesis III (mammals).metabolika|Superpathway of cytosolic glycolysis (plants), pyruvate dehydrogenase and TCA cycle.metabolika|Superpathway of D-glucarate and D-galactarate degradation.metabolika|Superpathway of D-myo-inositol (1,4,5)-trisphosphate metabolism.metabolika|Superpathway of demethylmenaquinol-6 biosynthesis I.metabolika|Superpathway of demethylmenaquinol-6 biosynthesis II.metabolika|Superpathway of demethylmenaquinol-8 biosynthesis.metabolika|Superpathway of demethylmenaquinol-9 biosynthesis.metabolika|Superpathway of dimethylsulfone degradation.metabolika|Superpathway of dimethylsulfoniopropanoate degradation.metabolika|Superpathway of diterpene resin acids biosynthesis.metabolika|Superpathway of dTDP-glucose-derived antibiotic building blocks biosynthesis.metabolika|Superpathway of dTDP-glucose-derived O-antigen building blocks biosynthesis.metabolika|Superpathway of ergosterol biosynthesis I.metabolika|Superpathway of ergosterol biosynthesis II.metabolika|Superpathway of ergotamine biosynthesis.metabolika|Superpathway of erythromycin biosynthesis (without sugar biosynthesis).metabolika|Superpathway of erythromycin biosynthesis.metabolika|Superpathway of fatty acid biosynthesis I (E. coli).metabolika|Superpathway of fatty acid biosynthesis II (plant).metabolika|Superpathway of fatty acid biosynthesis initiation (E. coli).metabolika|Superpathway of fatty acids biosynthesis (E. coli).metabolika|Superpathway of fermentation (Chlamydomonas reinhardtii).metabolika|Superpathway of flavones and derivatives biosynthesis .metabolika|Superpathway of formononetin derivative biosynthesis.metabolika|Superpathway of fucose and rhamnose degradation.metabolika|Superpathway of fumitremorgin biosynthesis.metabolika|Superpathway of GDP-mannose-derived O-antigen building blocks biosynthesis.metabolika|Superpathway of geranylgeranyl diphosphate biosynthesis II (via MEP).metabolika|Superpathway of geranylgeranyldiphosphate biosynthesis I (via mevalonate).metabolika|Superpathway of gibberellin biosynthesis.metabolika|Superpathway of gibberellin GA12 biosynthesis.metabolika|Superpathway of glucose and xylose degradation.metabolika|Superpathway of glycerol degradation to 1,3-propanediol.metabolika|Superpathway of glycol metabolism and degradation.metabolika|Superpathway of glycolysis and Entner-Doudoroff.metabolika|Superpathway of glycolysis, pyruvate dehydrogenase, TCA, and glyoxylate bypass.metabolika|Superpathway of glyoxylate bypass and TCA.metabolika|Superpathway of glyoxylate cycle and fatty acid degradation.metabolika|Superpathway of guanine and guanosine salvage.metabolika|Superpathway of guanosine nucleotides degradation (plants).metabolika|Superpathway of guanosine nucleotides de novo biosynthesis I.metabolika|Superpathway of guanosine nucleotides de novo biosynthesis II.metabolika|Superpathway of heme biosynthesis from glutamate.metabolika|Superpathway of heme biosynthesis from glycine.metabolika|Superpathway of heme biosynthesis from uroporphyrinogen-III.metabolika|Superpathway of hexitol degradation (bacteria).metabolika|Superpathway of hexuronide and hexuronate degradation.metabolika|Superpathway of histidine, purine, and pyrimidine biosynthesis.metabolika|Superpathway of hydrogen production.metabolika|Superpathway of hydrolyzable tannin biosynthesis.metabolika|Superpathway of hyoscyamine and scopolamine biosynthesis.metabolika|Superpathway of indole-3-acetate conjugate biosynthesis.metabolika|Superpathway of inositol phosphate compounds.metabolika|Superpathway of isoflavonoids (via naringenin).metabolika|Superpathway of jasmonoyl-amino acid conjugates biosynthesis.metabolika|Superpathway of L-alanine biosynthesis.metabolika|Superpathway of L-arginine and L-ornithine degradation.metabolika|Superpathway of L-arginine, putrescine, and 4-aminobutanoate degradation.metabolika|Superpathway of L-asparagine biosynthesis.metabolika|Superpathway of L-aspartate and L-asparagine biosynthesis.metabolika|Superpathway of L-citrulline metabolism.metabolika|Superpathway of L-cysteine biosynthesis (mammalian).metabolika|Superpathway of L-isoleucine biosynthesis I.metabolika|Superpathway of L-lysine degradation.metabolika|Superpathway of L-lysine, L-threonine and L-methionine biosynthesis I.metabolika|Superpathway of L-lysine, L-threonine and L-methionine biosynthesis

II.metabolika|Superpathway of L-methionine biosynthesis (by sulfhydrylation).metabolika|Superpathway of L-methionine biosynthesis (transsulfuration).metabolika|Superpathway of L-methionine salvage and degradation.metabolika|Superpathway of L-phenylalanine and L-tyrosine biosynthesis.metabolika|Superpathway of L-phenylalanine biosynthesis.metabolika|Superpathway of L-serine and glycine biosynthesis I.metabolika|Superpathway of L-threonine biosynthesis.metabolika|Superpathway of L-threonine metabolism.metabolika|Superpathway of L-tryptophan biosynthesis.metabolika|Superpathway of L-tyrosine biosynthesis.metabolika|Superpathway of linalool biosynthesis.metabolika|Superpathway of linamarin and lotaustralin biosynthesis.metabolika|Superpathway of lipopolysaccharide biosynthesis.metabolika|Superpathway of lipoxygenase.metabolika|Superpathway of megalomicin A biosynthesis.metabolika|Superpathway of melatonin degradation.metabolika|Superpathway of menaquinol-10 biosynthesis.metabolika|Superpathway of menaquinol-11 biosynthesis.metabolika|Superpathway of menaquinol-12 biosynthesis.metabolika|Superpathway of menaquinol-13 biosynthesis.metabolika|Superpathway of menaquinol-6 biosynthesis I.metabolika|Superpathway of menaquinol-7 biosynthesis.metabolika|Superpathway of menaquinol-8 biosynthesis I.metabolika|Superpathway of menaquinol-8 biosynthesis II.metabolika|Superpathway of menaquinol-9 biosynthesis.metabolika|Superpathway of methanogenesis.metabolika|Superpathway of methylglyoxal degradation.metabolika|Superpathway of microbial D-galacturonate and D-glucuronate degradation.metabolika|Superpathway of mycolyl-arabinogalactan-peptidoglycan complex biosynthesis.metabolika|Superpathway of NAD biosynthesis in eukaryotes.metabolika|Superpathway of neomycin biosynthesis.metabolika|Superpathway of nicotinate degradation.metabolika|Superpathway of nicotine biosynthesis.metabolika|Superpathway of oleoresin turpentine biosynthesis.metabolika|Superpathway of ornithine degradation.metabolika|Superpathway of penicillin, cephalosporin and cephamycin biosynthesis.metabolika|Superpathway of pentose and pentitol degradation.metabolika|Superpathway of phenylethylamine degradation.metabolika|Superpathway of phosphatidylcholine biosynthesis.metabolika|Superpathway of phospholipid biosynthesis I (bacteria).metabolika|Superpathway of phospholipid biosynthesis II (plants).metabolika|Superpathway of photosynthetic hydrogen production.metabolika|Superpathway of phylloquinol biosynthesis.metabolika|Superpathway of plastoquinol biosynthesis.metabolika|Superpathway of polyamine biosynthesis I.metabolika|Superpathway of polyamine biosynthesis II.metabolika|Superpathway of polyamine biosynthesis III.metabolika|Superpathway of pterocarpan biosynthesis (via daidzein).metabolika|Superpathway of pterocarpan biosynthesis (via formononetin).metabolika|Superpathway of purine deoxyribonucleosides degradation.metabolika|Superpathway of purine nucleotide salvage.metabolika|Superpathway of purine nucleotides de novo biosynthesis I.metabolika|Superpathway of purine nucleotides de novo biosynthesis II.metabolika|Superpathway of purines degradation in plants.metabolika|Superpathway of pyridoxal 5'-phosphate biosynthesis and salvage.metabolika|Superpathway of pyrimidine deoxyribonucleoside salvage.metabolika|Superpathway of pyrimidine deoxyribonucleosides degradation.metabolika|Superpathway of pyrimidine deoxyribonucleotides de novo biosynthesis (E. coli).metabolika|Superpathway of pyrimidine deoxyribonucleotides de novo biosynthesis.metabolika|Superpathway of pyrimidine nucleobases salvage.metabolika|Superpathway of pyrimidine ribonucleosides degradation.metabolika|Superpathway of pyrimidine ribonucleosides salvage.metabolika|Superpathway of pyrimidine ribonucleotides de novo biosynthesis.metabolika|Superpathway of quinolone and alkylquinolone biosynthesis.metabolika|Superpathway of rifamycin B biosynthesis.metabolika|Superpathway of roquefortine, meleagrin and neoxaline biosynthesis.metabolika|Superpathway of rosmarinic acid biosynthesis.metabolika|Superpathway of salicylate degradation.metabolika|Superpathway of scopolin and esculin biosynthesis.metabolika|Superpathway of seleno-compound metabolism.metabolika|Superpathway of ss-D-glucuronide and D-glucuronate degradation.metabolika|Superpathway of stearidonate biosynthesis (cyanobacteria).metabolika|Superpathway of steroid hormone biosynthesis.metabolika|Superpathway of

sulfate assimilation and cysteine biosynthesis.metabolika|Superpathway of sulfide oxidation (Acidithiobacillus ferrooxidans).metabolika|Superpathway of sulfide oxidation (phototrophic sulfur bacteria).metabolika|Superpathway of sulfide oxidation (Starkeya novella).metabolika|Superpathway of sulfolactate degradation.metabolika|Superpathway of sulfur amino acid biosynthesis (Saccharomyces cerevisiae).metabolika|Superpathway of sulfur metabolism (Desulfocapsa sulfoexigens).metabolika|Superpathway of sulfur oxidation (Acidianus ambivalens).metabolika|Superpathway of taurine degradation.metabolika|Superpathway of testosterone and androsterone degradation.metabolika|Superpathway of tetracycline and oxytetracycline biosynthesis.metabolika|Superpathway of tetrahydrofolate biosynthesis and salvage.metabolika|Superpathway of tetrahydrofolate biosynthesis.metabolika|Superpathway of tetrahydroxyxanthone biosynthesis.metabolika|Superpathway of tetrathionate reduction (Salmonella typhimurium).metabolika|Superpathway of the 3-hydroxypropanoate cycle.metabolika|Superpathway of thiamine diphosphate biosynthesis I.metabolika|Superpathway of thiamine diphosphate biosynthesis II.metabolika|Superpathway of thiamine diphosphate biosynthesis III (eukaryotes).metabolika|Superpathway of thiosulfate metabolism (Desulfovibrio sulfodismutans).metabolika|Superpathway of trichothecene biosynthesis.metabolika|Superpathway of trimethylamine degradation.metabolika|Superpathway of ubiquinol-6 biosynthesis (eukaryotic).metabolika|Superpathway of ubiquinol-8 biosynthesis (prokaryotic).metabolika|Superpathway of UDP-glucose-derived O-antigen building blocks biosynthesis.metabolika|Superpathway of UDP-N-acetylglucosamine-derived O-antigen building blocks biosynthesis.metabolika|Superpathway of unsaturated fatty acids biosynthesis (E. coli).metabolika|Superpathway of vanillin and vanillate degradation.metabolika|Superpathway of Clostridium acetobutylicum acidogenic and solventogenic fermentation.metabolika|Superpathway of Clostridium acetobutylicum acidogenic fermentation.metabolika|Superpathway of Clostridium acetobutylicum solventogenic fermentation.metabolika|Superpathway of N-acetylglucosamine, N-acetylmannosamine and N-acetylneuraminate degradation.metabolika|Superpathway of N-acetylneuraminate degradation.metabolika|Superpathway of S-adenosyl-L-methionine biosynthesis.metabolika|Superpathway polymethylated quercetinquercetagetin glucoside biosynthesis (Chrysosplenium).metabolika|Superpathways of coenzyme A biosynthesis I.metabolika|Superpathways of coenzyme A biosynthesis III (mammals).metabolika|Syringate degradation.metabolika|Taxadiene biosynthesis (engineered).metabolika|Thiamine salvage II.metabolika|Toluene degradation I (aerobic) (via o-cresol).metabolika|Toluene degradation II (aerobic) (via 4-methylcatechol).metabolika|Toluene degradation III (aerobic) (via p-cresol).metabolika|Toluene degradation IV (aerobic) (via catechol).metabolika|Toluene degradation V (aerobic) (via toluene-cis-diol).metabolika|Toluene degradation VI (anaerobic).metabolika|Trans-lycopene biosynthesis I (bacteria).metabolika|UDP-D-xylose biosynthesis.metabolika|UDP-galactofuranose biosynthesis.metabolika|UDP-sugars interconversion.metabolika|Ureide biosynthesis.metabolika|Vibriobactin biosynthesis.metabolika|Wybutosine biosynthesis.metabolika

- Search Mode: By Formula or Mass

2. By Mass Search Settings:

- Mass Tolerance: 5 ppm

3. By Formula Search Settings:

- Max. # of Predicted Compositions to be searched per Compound: 3

4. Display Settings:

- Max. # Pathways in 'Pathways' column: 20

Processing node 36: Search Mass Lists

----

1. Search Settings:

- Mass Lists: Arita Lab 6549 Flavonoid Structure Database.masslist|Natural Products Atlas 2020_06.massList|2020-10 COMPLETE HML Library_V12.massList|Prodelphinidin Compound Database_RT_2018Apr25.massList|Polyphenol Compound Database_RT_01FA_2020Jul_PulseSeedCoats.massList

- Mass Tolerance: 5 ppm

- Use Retention Time: True

- RT Tolerance [min]: 0.2

Processing node 29: Predict Compositions

----

1. Prediction Settings:

- Mass Tolerance: 5 ppm

- Min. Element Counts: C H

- Max. Element Counts: C120 H240 N4 O150 P S

- Min. RDBE: 0

- Max. RDBE: 40

- Min. H/C: 0.1

- Max. H/C: 4

- Max. # Candidates: 50

- Max. # Internal Candidates: 200

2. Pattern Matching:

- Intensity Tolerance [%]: 30

- Intensity Threshold [%]: 0.1

- S/N Threshold: 3

- Min. Spectral Fit [%]: 30

- Min. Pattern Cov. [%]: 90
- Use Dynamic Recalibration: True

3. Fragments Matching:
- Use Fragments Matching: False
- Mass Tolerance: 5 ppm
- S/N Threshold: 3

Processing node 25: Assign Compound Annotations

----

1. General Settings:
- Mass Tolerance: 5 ppm

2. Data Sources:
- Data Source #1: MassList Search
- Data Source #2: mzVault Search
- Data Source #3: mzCloud Search
- Data Source #4: Metabolika Search
- Data Source #5: Predicted Compositions
- Data Source #6: (not specified)
- Data Source #7: (not specified)

3. Scoring Rules:
- Use mzLogic: True
- Use Spectral Distance: True
- SFit Threshold: 20
- SFit Range: 20

Processing node 22: Search mzCloud

----

1. General Settings:

- Compound Classes: All
- Precursor Mass Tolerance: 10 ppm
- FT Fragment Mass Tolerance: 10 ppm
- IT Fragment Mass Tolerance: 0.4 Da
- Library: Autoprocessed; Reference
- Post Processing: Recalibrated
- Max. # Results: 10
- Annotate Matching Fragments: False

2. DDA Search:
- Identity Search: HighChem HighRes
- Match Activation Type: True
- Match Activation Energy: Match with Tolerance
- Activation Energy Tolerance: 40
- Apply Intensity Threshold: True
- Similarity Search: Confidence Reverse
- Match Factor Threshold: 50

3. DIA Search:
- Use DIA Scans for Search: False
- Max. Isolation Width [Da]: 500
- Match Activation Type: False
- Match Activation Energy: Any
- Activation Energy Tolerance: 100
- Apply Intensity Threshold: False
- Match Factor Threshold: 20

Processing node 37: Search mzVault

----

1. Search Settings:

- mzVault Library: Bamba lab 34 lipid mediators library stepped NCE 10 30 45.db|Bamba lab 598 polar metabolites stepped NCE 10 30 45.db|Polyphenol_CG_oligomers_2018May4.db|PP_2018May9.db|Negative ion mode_Jan2021.db

- Max. # Results: 10

- Match Factor Threshold: 50

- Search Algorithm: HighChem HighRes

- Match Analyzer Type: True

- IT Fragment Mass Tolerance: 0.4 Da

- FT Fragment Mass Tolerance: 10 ppm

- Use Retention Time: False

- Precursor Mass Tolerance: 10 ppm

- Apply Intensity Threshold: True

- Match Ionization Method: True

- Ion Activation Energy Tolerance: 50

- Match Ion Activation Energy: Match with Tolerance

- Match Ion Activation Type: True

- Compound Classes: All

- Remove Precursor Ion: True

- RT Tolerance [min]: 0.2

Processing node 17: Differential Analysis

----

1. General Settings:

- Log10 Transform Values: True

Processing node 38: Descriptive Statistics

No parameters

Filter

This file contains the following filters:

Row Filter for Compounds:

AND

|

+--BackgroundStatus is false

|

+--NormArea in any category

|

+--RT [min] is between 2.00 and 20.00

Study

=== Study: PatternedLentilSeedCoats_CD3.2_NoMottle_NoSpotted_NoGMarbled, 8/11/2021 10:39:17 PM ===

Directory: C:\Users\Public\Documents\Thermo\Compound Discoverer 3.2\Elessawy\PatternedSeedcoats_Mar2022

Factors:

Sample number

Options: 1, 4, 16, 93, 43, 258, 163, 174, 182, 211, 226, 310, 315, 44, 126, 210, 300, 136

Cotyledon color

Options: Yellow, Red

Field

Options: Sutherland, Rosthern, Pullman

Genotype

Options: CDC Asterix AGL, CDC Greenstar AGL, CDC Redwing AGL, ILL 5883 AGL, DPL 62 AGL, PI 178971 LSP AGL, PI 431739 LSP AGL, PI 289079 LSP AGL, PI 298122 LSP AGL, PI 299116 LSP AGL, PI 320954 LSP AGL, PI 374116 LSP AGL, PI 490289 LSP AGL, PI 533693 LSP AGL, Eston AGL, Indianhead AGL, PI 320953 LSP AGL, PI 472488 LSP AGL

Seed coat color

Options: Green, Dark green, Black

Seed coat pattern

Options: Absent, Dotted, Marbled, Black

Seed size

Options: M, XL, L, S

Samples and Files:

[S1] 190118QE_001 [Sample number=n/a, Cotyledon color=n/a, Field=n/a, Genotype=n/a, Seed coat color=n/a, Seed coat pattern=n/a, Seed size=n/a]

E:\QE Data\2019Jan18\190118QE_001.raw

[S2] 190118QE_002 [Sample number=n/a, Cotyledon color=n/a, Field=n/a, Genotype=n/a, Seed coat color=n/a, Seed coat pattern=n/a, Seed size=n/a]

E:\QE Data\2019Jan18\190118QE_002.raw

[S4] 190118QE_004 [Sample number=n/a, Cotyledon color=n/a, Field=n/a, Genotype=n/a, Seed coat color=n/a, Seed coat pattern=n/a, Seed size=n/a]

E:\QE Data\2019Jan18\190118QE_004.raw

[S5] 190118QE_005 [Sample number=n/a, Cotyledon color=n/a, Field=n/a, Genotype=n/a, Seed coat color=n/a, Seed coat pattern=n/a, Seed size=n/a]

E:\QE Data\2019Jan18\190118QE_005.raw

[S6] 190118QE_006 [Sample number=n/a, Cotyledon color=n/a, Field=n/a, Genotype=n/a, Seed coat color=n/a, Seed coat pattern=n/a, Seed size=n/a]

E:\QE Data\2019Jan18\190118QE_006.raw

[S7] 190118QE_007 [Sample number=n/a, Cotyledon color=n/a, Field=n/a, Genotype=n/a, Seed coat color=n/a, Seed coat pattern=n/a, Seed size=n/a]

E:\QE Data\2019Jan18\190118QE_007.raw

[S8] 190118QE_008 [Sample number=n/a, Cotyledon color=n/a, Field=n/a, Genotype=n/a, Seed coat color=n/a, Seed coat pattern=n/a, Seed size=n/a]

E:\QE Data\2019Jan18\190118QE_008.raw

[S9] 190118QE_009 [Sample number=n/a, Cotyledon color=n/a, Field=n/a, Genotype=n/a, Seed coat color=n/a, Seed coat pattern=n/a, Seed size=n/a]

E:\QE Data\2019Jan18\190118QE_009.raw

[S10] 190118QE_010 [Sample number=n/a, Cotyledon color=n/a, Field=n/a, Genotype=n/a, Seed coat color=n/a, Seed coat pattern=n/a, Seed size=n/a]

E:\QE Data\2019Jan18\190118QE_010.raw

[S11] 190118QE_011 [Sample number=n/a, Cotyledon color=n/a, Field=n/a, Genotype=n/a, Seed coat color=n/a, Seed coat pattern=n/a, Seed size=n/a]

E:\QE Data\2019Jan18\190118QE_011.raw

[S12] 190118QE_012 [Sample number=n/a, Cotyledon color=n/a, Field=n/a, Genotype=n/a, Seed coat color=n/a, Seed coat pattern=n/a, Seed size=n/a]

E:\QE Data\2019Jan18\190118QE_012.raw

[S13] 190118QE_013 [Sample number=n/a, Cotyledon color=n/a, Field=n/a, Genotype=n/a, Seed coat color=n/a, Seed coat pattern=n/a, Seed size=n/a]

E:\QE Data\2019Jan18\190118QE_013.raw

[S14] 190118QE_014 [Sample number=1, Cotyledon color=Yellow, Field=Pullman, Genotype=CDC Asterix AGL, Seed coat color=Green, Seed coat pattern=Absent, Seed size=M]

E:\QE Data\2019Jan18\190118QE_014.raw

[S15] 190118QE_015 [Sample number=4, Cotyledon color=Yellow, Field=Pullman, Genotype=CDC Greenstar AGL, Seed coat color=Green, Seed coat pattern=Absent, Seed size=XL]

E:\QE Data\2019Jan18\190118QE_015.raw

[S16] 190118QE_016 [Sample number=16, Cotyledon color=Red, Field=Pullman, Genotype=CDC Redwing AGL, Seed coat color=Green, Seed coat pattern=Absent, Seed size=M]

E:\QE Data\2019Jan18\190118QE_016.raw

[S17] 190118QE_017 [Sample number=43, Cotyledon color=Red, Field=Pullman, Genotype=DPL 62 AGL, Seed coat color=Green, Seed coat pattern=Dotted, Seed size=L]

E:\QE Data\2019Jan18\190118QE_017.raw

[S19] 190118QE_019 [Sample number=44, Cotyledon color=Yellow, Field=Pullman, Genotype=Eston AGL, Seed coat color=Green, Seed coat pattern=Absent, Seed size=M]

E:\QE Data\2019Jan18\190118QE_019.raw

[S21] 190118QE_021 [Sample number=n/a, Cotyledon color=n/a, Field=n/a, Genotype=n/a, Seed coat color=n/a, Seed coat pattern=n/a, Seed size=n/a]

E:\QE Data\2019Jan18\190118QE_021.raw

[S22] 190118QE_022 [Sample number=93, Cotyledon color=Red, Field=Pullman, Genotype=ILL 5883 AGL, Seed coat color=Green, Seed coat pattern=Absent, Seed size=L]

E:\QE Data\2019Jan18\190118QE_022.raw

[S23] 190118QE_023 [Sample number=126, Cotyledon color=Yellow, Field=Pullman, Genotype=Indianhead AGL, Seed coat color=Black, Seed coat pattern=Black, Seed size=S]

E:\QE Data\2019Jan18\190118QE_023.raw

[S24] 190118QE_024 [Sample number=136, Cotyledon color=Red, Field=Pullman, Genotype=PI 178971 LSP AGL, Seed coat color=Green, Seed coat pattern=Dotted, Seed size=S]

E:\QE Data\2019Jan18\190118QE_024.raw

[S26] 190118QE_026 [Sample number=163, Cotyledon color=Yellow, Field=Pullman, Genotype=PI 289079 LSP AGL, Seed coat color=Dark green, Seed coat pattern=Marbled, Seed size=L]

E:\QE Data\2019Jan18\190118QE_026.raw

[S27] 190118QE_027 [Sample number=174, Cotyledon color=Yellow, Field=Pullman, Genotype=PI 298122 LSP AGL, Seed coat color=Dark green, Seed coat pattern=Marbled, Seed size=M]

E:\QE Data\2019Jan18\190118QE_027.raw

[S28] 190118QE_028 [Sample number=182, Cotyledon color=Yellow, Field=Pullman, Genotype=PI 299116 LSP AGL, Seed coat color=Dark green, Seed coat pattern=Marbled, Seed size=S]

E:\QE Data\2019Jan18\190118QE_028.raw

[S29] 190118QE_029 [Sample number=n/a, Cotyledon color=n/a, Field=n/a, Genotype=n/a, Seed coat color=n/a, Seed coat pattern=n/a, Seed size=n/a]

E:\QE Data\2019Jan18\190118QE_029.raw

[S31] 190118QE_031 [Sample number=211, Cotyledon color=Yellow, Field=Pullman, Genotype=PI 320954 LSP AGL, Seed coat color=Dark green, Seed coat pattern=Marbled, Seed size=L]

E:\QE Data\2019Jan18\190118QE_031.raw

[S33] 190118QE_033 [Sample number=226, Cotyledon color=Yellow, Field=Pullman, Genotype=PI 374116 LSP AGL, Seed coat color=Dark green, Seed coat pattern=Marbled, Seed size=M]

E:\QE Data\2019Jan18\190118QE_033.raw

[S35] 190118QE_035 [Sample number=258, Cotyledon color=Yellow, Field=Pullman, Genotype=PI 431739 LSP AGL, Seed coat color=Green, Seed coat pattern=Dotted, Seed size=M]

E:\QE Data\2019Jan18\190118QE_035.raw

[S37] 190118QE_037 [Sample number=n/a, Cotyledon color=n/a, Field=n/a, Genotype=n/a, Seed coat color=n/a, Seed coat pattern=n/a, Seed size=n/a]

E:\QE Data\2019Jan18\190118QE_037.raw

[S38] 190118QE_038 [Sample number=300, Cotyledon color=Red, Field=Pullman, Genotype=PI 472488 LSP AGL, Seed coat color=Black, Seed coat pattern=Black, Seed size=S]

E:\QE Data\2019Jan18\190118QE_038.raw

[S39] 190118QE_039 [Sample number=310, Cotyledon color=Yellow, Field=Pullman, Genotype=PI 490289 LSP AGL, Seed coat color=Dark green, Seed coat pattern=Marbled, Seed size=L]

E:\QE Data\2019Jan18\190118QE_039.raw

[S40] 190118QE_040 [Sample number=315, Cotyledon color=Yellow, Field=Pullman, Genotype=PI 533693 LSP AGL, Seed coat color=Dark green, Seed coat pattern=Marbled, Seed size=M]

E:\QE Data\2019Jan18\190118QE_040.raw

[S41] 190118QE_041 [Sample number=1, Cotyledon color=Yellow, Field=Rosthern, Genotype=CDC Asterix AGL, Seed coat color=Green, Seed coat pattern=Absent, Seed size=M]

E:\QE Data\2019Jan18\190118QE_041.raw

[S42] 190118QE_042 [Sample number=4, Cotyledon color=Yellow, Field=Rosthern, Genotype=CDC Greenstar AGL, Seed coat color=Green, Seed coat pattern=Absent, Seed size=XL]

E:\QE Data\2019Jan18\190118QE_042.raw

[S43] 190118QE_043 [Sample number=16, Cotyledon color=Red, Field=Rosthern, Genotype=CDC Redwing AGL, Seed coat color=Green, Seed coat pattern=Absent, Seed size=M]

E:\QE Data\2019Jan18\190118QE_043.raw

[S44] 190118QE_044 [Sample number=43, Cotyledon color=Red, Field=Rosthern, Genotype=DPL 62 AGL, Seed coat color=Green, Seed coat pattern=Dotted, Seed size=L]

E:\QE Data\2019Jan18\190118QE_044.raw

[S45] 190118QE_045 [Sample number=n/a, Cotyledon color=n/a, Field=n/a, Genotype=n/a, Seed coat color=n/a, Seed coat pattern=n/a, Seed size=n/a]

E:\QE Data\2019Jan18\190118QE_045.raw

[S47] 190118QE_047 [Sample number=44, Cotyledon color=Yellow, Field=Rosthern, Genotype=Eston AGL, Seed coat color=Green, Seed coat pattern=Absent, Seed size=M]

E:\QE Data\2019Jan18\190118QE_047.raw

[S49] 190118QE_049 [Sample number=93, Cotyledon color=Red, Field=Rosthern, Genotype=ILL 5883 AGL, Seed coat color=Green, Seed coat pattern=Absent, Seed size=L]

E:\QE Data\2019Jan18\190118QE_049.raw

[S50] 190118QE_050 [Sample number=126, Cotyledon color=Yellow, Field=Rosthern, Genotype=Indianhead AGL, Seed coat color=Black, Seed coat pattern=Black, Seed size=S]

E:\QE Data\2019Jan18\190118QE_050.raw

[S51] 190118QE_051 [Sample number=136, Cotyledon color=Red, Field=Rosthern, Genotype=PI 178971 LSP AGL, Seed coat color=Green, Seed coat pattern=Dotted, Seed size=S]

E:\QE Data\2019Jan18\190118QE_051.raw

[S53] 190118QE_053 [Sample number=n/a, Cotyledon color=n/a, Field=n/a, Genotype=n/a, Seed coat color=n/a, Seed coat pattern=n/a, Seed size=n/a]

E:\QE Data\2019Jan18\190118QE_053.raw

[S54] 190118QE_054 [Sample number=163, Cotyledon color=Yellow, Field=Rosthern, Genotype=PI 289079 LSP AGL, Seed coat color=Dark green, Seed coat pattern=Marbled, Seed size=L]

E:\QE Data\2019Jan18\190118QE_054.raw

[S55] 190118QE_055 [Sample number=174, Cotyledon color=Yellow, Field=Rosthern, Genotype=PI 298122 LSP AGL, Seed coat color=Dark green, Seed coat pattern=Marbled, Seed size=M]

E:\QE Data\2019Jan18\190118QE_055.raw

[S56] 190118QE_056 [Sample number=182, Cotyledon color=Yellow, Field=Rosthern, Genotype=PI 299116 LSP AGL, Seed coat color=Dark green, Seed coat pattern=Marbled, Seed size=S]

E:\QE Data\2019Jan18\190118QE_056.raw

[S57] 190118QE_057 [Sample number=210, Cotyledon color=Red, Field=Rosthern, Genotype=PI 320953 LSP AGL, Seed coat color=Black, Seed coat pattern=Black, Seed size=S]

E:\QE Data\2019Jan18\190118QE_057.raw

[S58] 190118QE_058 [Sample number=211, Cotyledon color=Yellow, Field=Rosthern, Genotype=PI 320954 LSP AGL, Seed coat color=Dark green, Seed coat pattern=Marbled, Seed size=L]

E:\QE Data\2019Jan18\190118QE_058.raw

[S60] 190118QE_060 [Sample number=226, Cotyledon color=Yellow, Field=Rosthern, Genotype=PI 374116 LSP AGL, Seed coat color=Dark green, Seed coat pattern=Marbled, Seed size=M]

E:\QE Data\2019Jan18\190118QE_060.raw

[S61] 190118QE_061 [Sample number=n/a, Cotyledon color=n/a, Field=n/a, Genotype=n/a, Seed coat color=n/a, Seed coat pattern=n/a, Seed size=n/a]

E:\QE Data\2019Jan18\190118QE_061.raw

[S63] 190118QE_063 [Sample number=258, Cotyledon color=Yellow, Field=Rosthern, Genotype=PI 431739 LSP AGL, Seed coat color=Green, Seed coat pattern=Dotted, Seed size=M]

E:\QE Data\2019Jan18\190118QE_063.raw

[S65] 190118QE_065 [Sample number=300, Cotyledon color=Red, Field=Rosthern, Genotype=PI 472488 LSP AGL, Seed coat color=Black, Seed coat pattern=Black, Seed size=S]

E:\QE Data\2019Jan18\190118QE_065.raw

[S66] 190118QE_066 [Sample number=310, Cotyledon color=Yellow, Field=Rosthern, Genotype=PI 490289 LSP AGL, Seed coat color=Dark green, Seed coat pattern=Marbled, Seed size=L]

E:\QE Data\2019Jan18\190118QE_066.raw

[S67] 190118QE_067 [Sample number=315, Cotyledon color=Yellow, Field=Rosthern, Genotype=PI 533693 LSP AGL, Seed coat color=Dark green, Seed coat pattern=Marbled, Seed size=M]

E:\QE Data\2019Jan18\190118QE_067.raw

[S68] 190118QE_068 [Sample number=n/a, Cotyledon color=n/a, Field=n/a, Genotype=n/a, Seed coat color=n/a, Seed coat pattern=n/a, Seed size=n/a]

E:\QE Data\2019Jan18\190118QE_068.raw

[S69] 190118QE_069 [Sample number=1, Cotyledon color=Yellow, Field=Sutherland, Genotype=CDC Asterix AGL, Seed coat color=Green, Seed coat pattern=Absent, Seed size=M]

E:\QE Data\2019Jan18\190118QE_069.raw

[S70] 190118QE_070 [Sample number=4, Cotyledon color=Yellow, Field=Sutherland, Genotype=CDC Greenstar AGL, Seed coat color=Green, Seed coat pattern=Absent, Seed size=XL]

E:\QE Data\2019Jan18\190118QE_070.raw

[S71] 190118QE_071 [Sample number=16, Cotyledon color=Red, Field=Sutherland, Genotype=CDC Redwing AGL, Seed coat color=Green, Seed coat pattern=Absent, Seed size=M]

E:\QE Data\2019Jan18\190118QE_071.raw

[S72] 190118QE_072 [Sample number=43, Cotyledon color=Red, Field=Sutherland, Genotype=DPL 62 AGL, Seed coat color=Green, Seed coat pattern=Dotted, Seed size=L]

E:\QE Data\2019Jan18\190118QE_072.raw

[S74] 190118QE_074 [Sample number=44, Cotyledon color=Yellow, Field=Sutherland, Genotype=Eston AGL, Seed coat color=Green, Seed coat pattern=Absent, Seed size=M]

E:\QE Data\2019Jan18\190118QE_074.raw

[S76] 190118QE_076 [Sample number=n/a, Cotyledon color=n/a, Field=n/a, Genotype=n/a, Seed coat color=n/a, Seed coat pattern=n/a, Seed size=n/a]

E:\QE Data\2019Jan18\190118QE_076.raw

[S77] 190118QE_077 [Sample number=93, Cotyledon color=Red, Field=Sutherland, Genotype=ILL 5883 AGL, Seed coat color=Green, Seed coat pattern=Absent, Seed size=L]

E:\QE Data\2019Jan18\190118QE_077.raw

[S78] 190118QE_078 [Sample number=126, Cotyledon color=Yellow, Field=Sutherland, Genotype=Indianhead AGL, Seed coat color=Black, Seed coat pattern=Black, Seed size=S]

E:\QE Data\2019Jan18\190118QE_078.raw

[S79] 190118QE_079 [Sample number=136, Cotyledon color=Red, Field=Sutherland, Genotype=PI 178971 LSP AGL, Seed coat color=Green, Seed coat pattern=Dotted, Seed size=S]

E:\QE Data\2019Jan18\190118QE_079.raw

[S81] 190118QE_081 [Sample number=163, Cotyledon color=Yellow, Field=Sutherland, Genotype=PI 289079 LSP AGL, Seed coat color=Dark green, Seed coat pattern=Marbled, Seed size=L]

E:\QE Data\2019Jan18\190118QE_081.raw

[S82] 190118QE_082 [Sample number=174, Cotyledon color=Yellow, Field=Sutherland, Genotype=PI 298122 LSP AGL, Seed coat color=Dark green, Seed coat pattern=Marbled, Seed size=M]

E:\QE Data\2019Jan18\190118QE_082.raw

[S83] 190118QE_083 [Sample number=182, Cotyledon color=Yellow, Field=Sutherland, Genotype=PI 299116 LSP AGL, Seed coat color=Dark green, Seed coat pattern=Marbled, Seed size=S]

E:\QE Data\2019Jan18\190118QE_083.raw

[S84] 190118QE_084 [Sample number=n/a, Cotyledon color=n/a, Field=n/a, Genotype=n/a, Seed coat color=n/a, Seed coat pattern=n/a, Seed size=n/a]

E:\QE Data\2019Jan18\190118QE_084.raw

[S85] 190118QE_085 [Sample number=210, Cotyledon color=Red, Field=Sutherland, Genotype=PI 320953 LSP AGL, Seed coat color=Black, Seed coat pattern=Black, Seed size=S]

E:\QE Data\2019Jan18\190118QE_085.raw

[S86] 190118QE_086 [Sample number=211, Cotyledon color=Yellow, Field=Sutherland, Genotype=PI 320954 LSP AGL, Seed coat color=Dark green, Seed coat pattern=Marbled, Seed size=L]

E:\QE Data\2019Jan18\190118QE_086.raw

[S91] 190118QE_090 [Sample number=226, Cotyledon color=Yellow, Field=Sutherland, Genotype=PI 374116 LSP AGL, Seed coat color=Dark green, Seed coat pattern=Marbled, Seed size=M]

E:\QE Data\2019Jan18\190118QE_090.raw

[S93] 190118QE_092 [Sample number=258, Cotyledon color=Yellow, Field=Sutherland, Genotype=PI 431739 LSP AGL, Seed coat color=Green, Seed coat pattern=Dotted, Seed size=M]

E:\QE Data\2019Jan18\190118QE_092.raw

[S95] 190118QE_094 [Sample number=n/a, Cotyledon color=n/a, Field=n/a, Genotype=n/a, Seed coat color=n/a, Seed coat pattern=n/a, Seed size=n/a]

E:\QE Data\2019Jan18\190118QE_094.raw

[S96] 190118QE_095 [Sample number=300, Cotyledon color=Red, Field=Sutherland, Genotype=PI 472488 LSP AGL, Seed coat color=Black, Seed coat pattern=Black, Seed size=S]

E:\QE Data\2019Jan18\190118QE_095.raw

[S97] 190118QE_096 [Sample number=310, Cotyledon color=Yellow, Field=Sutherland, Genotype=PI 490289 LSP AGL, Seed coat color=Dark green, Seed coat pattern=Marbled, Seed size=L]

E:\QE Data\2019Jan18\190118QE_096.raw

[S98] 190118QE_097 [Sample number=315, Cotyledon color=Yellow, Field=Sutherland, Genotype=PI 533693 LSP AGL, Seed coat color=Dark green, Seed coat pattern=Marbled, Seed size=M]

E:\QE Data\2019Jan18\190118QE_097.raw

[S99] 190118QE_098 [Sample number=210, Cotyledon color=Red, Field=Pullman, Genotype=PI 320953 LSP AGL, Seed coat color=Black, Seed coat pattern=Black, Seed size=S]

E:\QE Data\2019Jan18\190118QE_098.raw

[S101] 190118QE_100 [Sample number=n/a, Cotyledon color=n/a, Field=n/a, Genotype=n/a, Seed coat color=n/a, Seed coat pattern=n/a, Seed size=n/a]

E:\QE Data\2019Jan18\190118QE_100.raw

Grouping and Ratios

Grouping:

Study variable(s) for grouping:

Seed coat color;Seed coat pattern

Sample Groups:

Black, Black

F23: Sample, 126, Yellow, Pullman, Indianhead AGL, Black, Black, S

F38: Sample, 300, Red, Pullman, PI 472488 LSP AGL, Black, Black, S

F50: Sample, 126, Yellow, Rosthern, Indianhead AGL, Black, Black, S

F57: Sample, 210, Red, Rosthern, PI 320953 LSP AGL, Black, Black, S

F65: Sample, 300, Red, Rosthern, PI 472488 LSP AGL, Black, Black, S

F78: Sample, 126, Yellow, Sutherland, Indianhead AGL, Black, Black, S

F85: Sample, 210, Red, Sutherland, PI 320953 LSP AGL, Black, Black, S

F96: Sample, 300, Red, Sutherland, PI 472488 LSP AGL, Black, Black, S

F99: Sample, 210, Red, Pullman, PI 320953 LSP AGL, Black, Black, S

Dark green, Marbled

F26: Sample, 163, Yellow, Pullman, PI 289079 LSP AGL, Dark green, Marbled, L

F27: Sample, 174, Yellow, Pullman, PI 298122 LSP AGL, Dark green, Marbled, M

F28: Sample, 182, Yellow, Pullman, PI 299116 LSP AGL, Dark green, Marbled, S

F31: Sample, 211, Yellow, Pullman, PI 320954 LSP AGL, Dark green, Marbled, L

F33: Sample, 226, Yellow, Pullman, PI 374116 LSP AGL, Dark green, Marbled, M

F39: Sample, 310, Yellow, Pullman, PI 490289 LSP AGL, Dark green, Marbled, L

F40: Sample, 315, Yellow, Pullman, PI 533693 LSP AGL, Dark green, Marbled, M

F54: Sample, 163, Yellow, Rosthern, PI 289079 LSP AGL, Dark green, Marbled, L

F55: Sample, 174, Yellow, Rosthern, PI 298122 LSP AGL, Dark green, Marbled, M

F56: Sample, 182, Yellow, Rosthern, PI 299116 LSP AGL, Dark green, Marbled, S

F58: Sample, 211, Yellow, Rosthern, PI 320954 LSP AGL, Dark green, Marbled, L

F60: Sample, 226, Yellow, Rosthern, PI 374116 LSP AGL, Dark green, Marbled, M

F66: Sample, 310, Yellow, Rosthern, PI 490289 LSP AGL, Dark green, Marbled, L

F67: Sample, 315, Yellow, Rosthern, PI 533693 LSP AGL, Dark green, Marbled, M

F81: Sample, 163, Yellow, Sutherland, PI 289079 LSP AGL, Dark green, Marbled, L

F82: Sample, 174, Yellow, Sutherland, PI 298122 LSP AGL, Dark green, Marbled, M

F83: Sample, 182, Yellow, Sutherland, PI 299116 LSP AGL, Dark green, Marbled, S

F86: Sample, 211, Yellow, Sutherland, PI 320954 LSP AGL, Dark green, Marbled, L

F91: Sample, 226, Yellow, Sutherland, PI 374116 LSP AGL, Dark green, Marbled, M

F97: Sample, 310, Yellow, Sutherland, PI 490289 LSP AGL, Dark green, Marbled, L

F98: Sample, 315, Yellow, Sutherland, PI 533693 LSP AGL, Dark green, Marbled, M

Green, Absent

F14: Sample, 1, Yellow, Pullman, CDC Asterix AGL, Green, Absent, M

F15: Sample, 4, Yellow, Pullman, CDC Greenstar AGL, Green, Absent, XL

F16: Sample, 16, Red, Pullman, CDC Redwing AGL, Green, Absent, M

F19: Sample, 44, Yellow, Pullman, Eston AGL, Green, Absent, M

F22: Sample, 93, Red, Pullman, ILL 5883 AGL, Green, Absent, L

F41: Sample, 1, Yellow, Rosthern, CDC Asterix AGL, Green, Absent, M

F42: Sample, 4, Yellow, Rosthern, CDC Greenstar AGL, Green, Absent, XL

F43: Sample, 16, Red, Rosthern, CDC Redwing AGL, Green, Absent, M

F47: Sample, 44, Yellow, Rosthern, Eston AGL, Green, Absent, M

F49: Sample, 93, Red, Rosthern, ILL 5883 AGL, Green, Absent, L

F69: Sample, 1, Yellow, Sutherland, CDC Asterix AGL, Green, Absent, M

F70: Sample, 4, Yellow, Sutherland, CDC Greenstar AGL, Green, Absent, XL

F71: Sample, 16, Red, Sutherland, CDC Redwing AGL, Green, Absent, M

F74: Sample, 44, Yellow, Sutherland, Eston AGL, Green, Absent, M

F77: Sample, 93, Red, Sutherland, ILL 5883 AGL, Green, Absent, L

Green, Dotted

F17: Sample, 43, Red, Pullman, DPL 62 AGL, Green, Dotted, L

F24: Sample, 136, Red, Pullman, PI 178971 LSP AGL, Green, Dotted, S

F35: Sample, 258, Yellow, Pullman, PI 431739 LSP AGL, Green, Dotted, M

F44: Sample, 43, Red, Rosthern, DPL 62 AGL, Green, Dotted, L

F51: Sample, 136, Red, Rosthern, PI 178971 LSP AGL, Green, Dotted, S

F63: Sample, 258, Yellow, Rosthern, PI 431739 LSP AGL, Green, Dotted, M

F72: Sample, 43, Red, Sutherland, DPL 62 AGL, Green, Dotted, L

F79: Sample, 136, Red, Sutherland, PI 178971 LSP AGL, Green, Dotted, S

F93: Sample, 258, Yellow, Sutherland, PI 431739 LSP AGL, Green, Dotted, M

n/a, n/a

F1: Blank, n/a, n/a, n/a, n/a, n/a, n/a, n/a

F2: Blank, n/a, n/a, n/a, n/a, n/a, n/a, n/a

F4: Blank, n/a, n/a, n/a, n/a, n/a, n/a, n/a

F5: Blank, n/a, n/a, n/a, n/a, n/a, n/a, n/a

F6: IdentificationOnly, n/a, n/a, n/a, n/a, n/a, n/a, n/a

F7: IdentificationOnly, n/a, n/a, n/a, n/a, n/a, n/a, n/a

F8: IdentificationOnly, n/a, n/a, n/a, n/a, n/a, n/a, n/a

F9: IdentificationOnly, n/a, n/a, n/a, n/a, n/a, n/a, n/a

F10: IdentificationOnly, n/a, n/a, n/a, n/a, n/a, n/a, n/a

F11: IdentificationOnly, n/a, n/a, n/a, n/a, n/a, n/a, n/a

F12: IdentificationOnly, n/a, n/a, n/a, n/a, n/a, n/a, n/a

F13: QualityControl, n/a, n/a, n/a, n/a, n/a, n/a, n/a

F21: QualityControl, n/a, n/a, n/a, n/a, n/a, n/a, n/a

F29: QualityControl, n/a, n/a, n/a, n/a, n/a, n/a, n/a

F37: QualityControl, n/a, n/a, n/a, n/a, n/a, n/a, n/a

F45: QualityControl, n/a, n/a, n/a, n/a, n/a, n/a, n/a

F53: QualityControl, n/a, n/a, n/a, n/a, n/a, n/a, n/a

F61: QualityControl, n/a, n/a, n/a, n/a, n/a, n/a, n/a

F68: QualityControl, n/a, n/a, n/a, n/a, n/a, n/a, n/a

F76: QualityControl, n/a, n/a, n/a, n/a, n/a, n/a, n/a

F84: QualityControl, n/a, n/a, n/a, n/a, n/a, n/a, n/a

F95: QualityControl, n/a, n/a, n/a, n/a, n/a, n/a, n/a

F101: QualityControl, n/a, n/a, n/a, n/a, n/a, n/a, n/a

Ratios:

(Black, Black) / (Dark green, Marbled)

(Black, Black) / (Green, Absent)

(Black, Black) / (Green, Dotted)

(Dark green, Marbled) / (Green, Absent)

(Dark green, Marbled) / (Green, Dotted)

(Green, Dotted) / (Green, Absent)
